# Investigating the Effect of Ligand Amount and Injected Therapeutic Activity: A Simulation Study for ^177^Lu-Labeled PSMA-Targeting Peptides

**DOI:** 10.1371/journal.pone.0162303

**Published:** 2016-09-09

**Authors:** Peter Kletting, Christiane Schuchardt, Harshad R. Kulkarni, Mostafa Shahinfar, Aviral Singh, Gerhard Glatting, Richard P. Baum, Ambros J. Beer

**Affiliations:** 1 Department of Nuclear Medicine, Ulm University, Ulm, Germany; 2 THERANOSTICS Center for Molecular Radiotherapy and Molecular Imaging (PET/CT), Zentralklinik Bad Berka, Bad Berka, Germany; 3 Medical Radiation Physics/Radiation Protection, Universitätsmedizin Mannheim, Medical Faculty Mannheim, Heidelberg University, Mannheim, Germany; Center for Cancer Research, UNITED STATES

## Abstract

In molecular radiotherapy with ^177^Lu-labeled prostate specific membrane antigen (PSMA) peptides, kidney and/or salivary glands doses limit the activity which can be administered. The aim of this work was to investigate the effect of the ligand amount and injected activity on the tumor-to-normal tissue biologically effective dose (BED) ratio for ^177^Lu-labeled PSMA peptides. For this retrospective study, a recently developed physiologically based pharmacokinetic model was adapted for PSMA targeting peptides. General physiological parameters were taken from the literature. Individual parameters were fitted to planar gamma camera measurements (^177^Lu-PSMA I&T) of five patients with metastasizing prostate cancer. Based on the estimated parameters, the pharmacokinetics of tumor, salivary glands, kidneys, total body and red marrow was simulated and time-integrated activity coefficients were calculated for different peptide amounts. Based on these simulations, the absorbed doses and BEDs for normal tissue and tumor were calculated for all activities leading to a maximal tolerable kidney BED of 10 Gy_2.5_/cycle, a maximal salivary gland absorbed dose of 7.5 Gy/cycle and a maximal red marrow BED of 0.25 Gy_15_/cycle. The fits yielded coefficients of determination > 0.85, acceptable relative standard errors and low parameter correlations. All estimated parameters were in a physiologically reasonable range. The amounts (for 2^5^−2^9^ nmol) and pertaining activities leading to a maximal tumor dose, considering the defined maximal tolerable doses to organs of risk, were calculated to be 272±253 nmol (452±420 μg) and 7.3±5.1 GBq. Using the actually injected amount (235±155 μg) and the same maximal tolerable doses, the potential improvement for the tumor BED was 1–3 fold. The results suggest that currently given amounts for therapy are in the appropriate order of magnitude for many lesions. However, for lesions with high binding site density or lower perfusion, optimizing the peptide amount and activity might improve the tumor-to-kidney and tumor-to-salivary glands BED ratio considerably.

## Introduction

Molecular radiotherapy using ^177^Lu-labeled PSMA specific peptides is a promising novel approach for the treatment of metastatic prostate cancer [1–3]. However, PSMA is also expressed with relevant density in the salivary glands [4] and kidneys [5], where specific binding in combination with (possible) unspecific uptake can lead to considerable absorbed doses [6, 7]. To increase the tumor-to-kidney absorbed dose or biologically effective dose (BED) ratio, amino acids are administered to block unspecific uptake at some institutions analogous to peptide receptor radionuclide therapy (PRRT), however evidence on the relevance of this approach for PSMA-targeted radionuclide therapy is still missing. In addition, research is directed to block PSMA specific uptake using 2-(phosphonomethyl)pentanedioic acid (PMPA) [8]. However, the effect of the peptide amount and the administered activity on the tumor, kidneys, salivary glands and red marrow absorbed dose and BED has not been investigated. For a systematic investigation, physiologically based pharmacokinetic (PBPK) modeling, that allows simulating the pharmacokinetics for different peptide amounts using general physiological and patient individual parameters estimated based on imaging data, represents a suitable method [9, 10]. To ensure the clinical relevance of these simulations, the pharmacokinetic model should be combined with absorbed dose and BED calculations and boundary conditions such as the maximal tolerable dose for critical organs. Recently, we have developed and demonstrated such a method for optimizing the peptide amount and activity for molecular radiotherapy with ^90^Y-DOTATATE [11] to improve the tumor-to-kidney BED ratio. A high tumor-to-kidney ratio for binding site density together with a low ratio for tumor-to-kidney perfusion resulted in a strong influence of the peptide amount on the BED ratio. These results were in agreement with other studies for similar substances [12, 13]. We conjectured an even stronger effect for PSMA targeting ligands, as high tumor binding site densities compared to normal tissue have been reported in vitro [14–16].

Therefore, the aim of this work was to investigate the effect of ligand amount and injected activity on the tumor-to-kidney and tumor-to-salivary gland BED ratio for therapy with ^177^Lu-labeled PSMA peptides. Maximal tolerable doses of the kidneys, salivary glands and red marrow were assumed. For that purpose a whole body PBPK model and a simulation algorithm were implemented and adapted to PSMA ligands [11]. Patient individual parameters were fitted to time-activity data of five patients with metastasizing prostate cancer treated with 5.6±0.3 GBq ^177^Lu-PSMA I&T. Based on the individually estimated parameters, the tumor, kidney, salivary gland and red marrow pharmacokinetics were simulated for large ranges of peptide amounts and activities. Absorbed doses and BEDs were calculated considering a maximal red marrow BED of 0.25 Gy_15_/cycle, a maximal kidney BED of 10 Gy_2.5_/cycle and/or a maximal salivary gland absorbed dose of 7.5 Gy/cycle. The maximal tolerable doses were derived from literature values assuming four cycles with equal biodistribution. The effect of the tumor perfusion on the predicted BED for different amounts was also investigated.

## Materials and Methods

### Patients/Data

Data of five patients (one cycle per patient) with metastasizing prostate cancer were included (Table 1). For patient 1 data of the second cycle, for patients 2–5 data of the first cycle were used. Before PSMA radioligand therapy, each patient was informed about the therapeutic procedure and possible adverse effects. ^177^Lu-PSMA was administered in compliance with the German Medicinal Products Act, AMG (§13, 2b), the 1964 Helsinki declaration, and in accordance with the responsible regulatory body (Government of Thuringia, Germany). The study was performed in accordance with German regulations (Federal Agency for Radiation Protection) concerning radiation safety and was approved by the local ethics committee (Bad Berka, Germany). Written informed consent was obtained from all patients.

**Table 1:** Patient basic information and measurements.

Patient	Age	BSA [m^2^]	Amount^a^ [nmol]	Activity[GBq]	TER[ml/min]	Measured volumes [ml]			
						Parotidglands^b^	Kidneys^b^	Tumor 1	Tumor 2
P1	76	2.0	148	6.0	198	54	321	0.5	1
P2	69	1.9	98	5.4	201	21	311	1	34
P3	78	1.8	89	5.4	136	17	394	2	13
P4	54	2.1	74	5.4	252	52	268	4	3
P5	53	2.0	302	5.6	176	29	296	1.5	1
Mean	66	2.0	142	5.6	193	35	318	6	
SD	12	0.1	94	0.3	42	17	47	10	

Abbreviation: BSA = Body surface area, TER = tubular extraction rate as determined with the ^99m^Tc Mag_3_ method.

^a^1.66 μg of the ligand corresponds to 1 nmol.

^b^The volumes of the parotid glands as well as the kidney volumes were added to one volume.

The patient selection criteria for therapy are described in [17]. PSMA expression of tumors and metastatic lesions was verified before therapy using ^68^Ga-PSMA PET/CT (^68^Ga-PSMA-HBED-CC). The administered therapeutic activity, number of cycles and interval between cycles were selected based on the uptake in tumor lesions on pre-therapeutic ^68^Ga-PSMA PET/CT, kidney function (serum creatinine, tubular extraction rate, TER determined by ^99^mTc-MAG3 scintigraphy and creatinine-clearance), hematological reserve, previous treatments and general status of the patient (KPS). The administered activity in this patient group was relatively homogeneous with 5.6±0.3 GBq ^177^Lu-PSMA.

**Fig 1:**
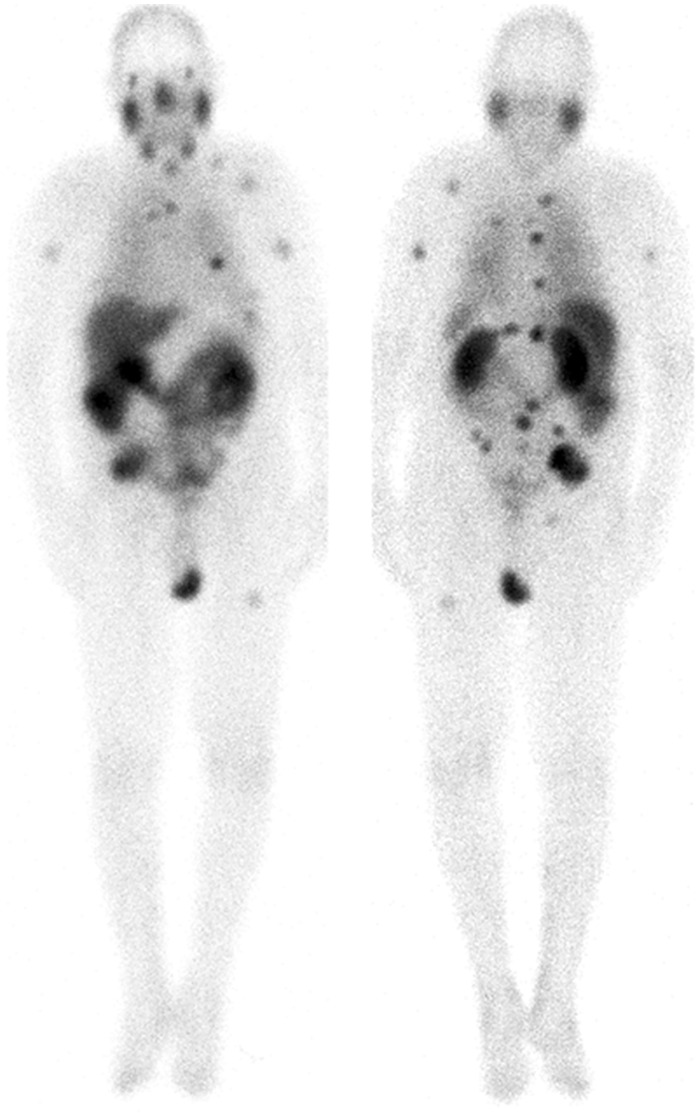
Typical biodistribution. Typical biodistribution 20 h after injection (anterior and posterior gamma camera images) of patient one. The salivary glands, kidneys and tumor lesions show highest uptake. Two bone lesions were analyzed.

For all patients γ-camera imaging was performed at least 4 times after beginning of the therapy infusion (Fig 1). P1: 0.5 h, 2 h, 1 d, 2 d, 3 d; P2: 0.5 h, 2 h, 1 d, 2 d, 3 d; P3: 0.5 h, 2 h, 1 d, 4 d; P4: 0.5 h, 2 h, 1 d, 2 d, 5 d; P5: 0.5 h, 2 h, 1 d, 2 d, 3 d. The kidney function, i.e. the tubular extraction rate (TER), was determined using the ^99m^Tc-Mag_3_ method [18].

#### Labeling and amino acid infusion

Lutetium-177 labeling of the PSMA I&T ligand was performed according to a previously published method [19]. For therapy 236±155 μg PSMA labeled with 5.6±0.3 GBq ^177^Lu were intravenously injected as a 10 min infusion. Lysine and arginine (1000 ml, 2.5% infusion) were co-administered over a period of 2 h, starting 0.5 h prior to the administration of ^177^Lu-PSMA.

#### Data acquisition and processing

Data acquisition and processing was based on MIRD pamphlets 16 [20] and 21 [21] as described in [17, 22]. In brief, planar whole-body scintigraphies (anterior and posterior) were acquired with a double-head γ-camera (MEDISO spirit DH-V, Medical Imaging Systems, Budapest, Hungary). Regions of interest (ROIs) were drawn manually over the source organs. First, ROIs were drawn by using the scan showing the best tumor to background ratio (mostly the scan acquired 20 or 44 h following an injection). Then, the ROIs were applied to the other 4 scans and the respective counts were obtained. The scintigraphies were analyzed using the HERMES system (Hermes Medical Solutions, Stockholm, Sweden). To quantify the ROI data, the geometric mean of anterior and posterior counts with background corrections was used for each organ and target lesion ROI. Based on the corrected counts, the time-dependent activities for the considered regions were calculated [22]. ROIs were always drawn by the same physicist (C.S.), in collaboration with a nuclear medicine physician, who decided which lesions were relevant for dosimetry. In this study, ROIs were drawn for two tumor lesions (bone and lymph nodes), parotid glands, kidneys, and total body.

The volume of the tumor lesions, left and right parotid glands and the kidneys were determined using CT [23]. Lesions showing high uptake and no superimposition with organs or other lesions were selected.

In the following calculations, the term salivary glands is synonymous to the total volume and activity of the right and left parotid glands. The parotid glands are assumed representative for all salivary glands. The relatively large volume makes them most suitable for activity determination from planar images.

### PBPK model

#### PBPK Model structure

The global structure of a recently developed whole-body PBPK model [11] was applied. The model includes all major physiological and physical mechanisms, i.e. blood flow, extravasation, specific binding, internalization, degradation and release, physical decay and clearance [11]. Labeled and unlabeled peptides are described separately (assuming equal physiological parameters) and coupled by the competition for binding to free binding sites and by physical decay. This model (structure and parameters) was adapted to PSMA ligands, i.e. the adrenals were replaced by the salivary glands and all parameter values describing target-ligand interaction were substituted with the PSMA I&T relevant values, as described in S1 File and below. Tumor, salivary glands, kidney, liver, spleen, gastrointestinal tract and prostate were assumed to be accessible PSMA positive tissues [5, 24]. Muscle, fat tissue, lungs, bone, red marrow, heart, brain and skin were explicitly modeled non-PSMA expressing normal tissues. All organs are connected via blood flow. Extravasation to the interstitial spaces is diffusion dominated for the small peptides. Therefore, transcapillary transport is described by the permeability surface area product and the vascular and interstitial volumes of the pertaining tissue. The complex internalization (recycling), metabolism and release process was described with one internalization and one release rate, representing effective values. It is also assumed that unspecific kidney uptake is predominately blocked by the administration of amino acids [11] although this is not proven for PSMA I&T.

#### PBPK model parameters and model fitting

SAAMII [25] (version 2.2, The Epsilon Group, Washington, USA) was employed for modeling and fitting using the settings as described earlier [9]. All parameters and the pertaining values are taken from literature and given in Table A in S1 File. Cell release rates λ_i,release_ for tumor, kidney and salivary glands were fitted. The binding site density of tumor lesions [R_TU,0_], kidneys [R_K,0_] and salivary glands [R_SAL,0_] were fitted, for all other tissue relative values according to [5, 24] were assumed. Internalization rates were fixed to λ_i_,_int_ = 0.001 min^-1^ for all organs and tumor [11]. Weineisen et al. report rapid internalization [26], however, preliminary fits with λ_i_,_int_ = 0.01 min^-1^ did not lead to adequate results, i.e. low coefficients of determination, high parameter standard deviations and low Akaike weights compared to fits using λ_i_,_int_ = 0.001 min^-1^. For the tumor, a perfusion rate f_TU_ of 0.5 ml·g^-1^·min^-1^ was assumed [27]. The adjustable parameters (Table 2, S1 and S2 Files) were fitted to the time activity data of each patient. The number of free parameters was 10 for each patient and the sample size 20 for patient 2 and 25 for all other patients. Note that relative fitting was used to estimate the variance for the data set of each organ. Thus the ratio of sample size to free parameters (+1 for the variance) was 1.3–1.7, which is acceptable.

**Table 2:** Fitted physiological/pathophysiological parameters.

Parameter	[R_K,0_]	[R_SAL,0_]	[R_TU,0_]	λ_K,release_^†^	λ_SAL,release_	λ_TU,release_^†^	f_SAL_
	[nmol·l^-1^]			[min^-1^·10^−4^]			[ml·g^-1^·min^-1^]
**Mean**	27	62	380	2.88	3.90	1.76	0.20
**SD**	12	31	620	0.55	0.63	0.58	0.19
**Median**	24	42	110	2.9	3.7	2.0	0.16
**Min**	14	38	19	2.3	3.3	0.88	0.074
**Max**	46	108	2412	3.7	4.8	2.4	0.53
**Literature**	-	-	266–1328^a^	0.5–2.3^b^	-	0–3^b^	0.34±0.09^c^

Abbreviation: [R_i,0_] binding site density of kidneys, salivary glands and tumor, λ_i,release_ release rate of kidneys, salivary glands and tumor, f_SAL_ serum flow per mass of salivary glands

^a^ Assuming 10^12^ cells/l and 180.000 [16] and 800.000 [14] copies of PSMA/cell from in vitro measurements

^b^ For ^111^In-DOTATATE [11]

^c^ [42] non-stimulated blood flow per mass

Although background correction using a ROI on the thigh and equation 6 from MIRD 16 [20] was applied, inaccuracies of the correction method substantially affect early measurements. This is due to different tissue thicknesses, different background activities or a considerable larger tumor ROI diameter (especially for small lesions) compared to the actual tumor diameter. The tumor volume was determined using CT. Therefore, as we were interested in the underlying physiology (not only in the time-integrated activity coefficient) like the binding site density, an additional correction was necessary. This was done by assigning a fraction (adjustable parameter) to the muscle compartments (S1 File, page 19). PBPK models allow such corrections by assigning the measurements to the compartments which contribute to the total measurement value.

#### PBPK Model validation and selection

Reported values for the PSMA ligand dissociations constants from in vitro cell studies (~ 8 nM, [26]) and BiaCore measurements (~ 1 nM, [28]) differ considerably. Therefore, two different dissociation rates k_off_, i.e. 0.046·min^-1^ (Model 1) and 0.46·min^-1^ (Model 2), were assumed. The association rate was fixed to k_on_ = 0.046 l·nmol^-1^·min^-1^ [28]. The models were compared using the Akaike information criterion (AIC) [29, 30]. The AIC represents an elegant method to select the model most supported by the data. It is suitable for the data at hand [29, 30] and requires solely the sums of squares and the numbers of measurements and adjustable parameters (+1 if the variance is also estimated). The fits were validated by visual inspection, coefficients of determination and correlation, physiological plausibility and standard deviations of the estimated parameters [31].

### Simulations

The simulations were conducted to investigate the effect of the peptide amount on the tumor-to-normal tissue absorbed dose or BED ratio. For the simulations, the PBPK model which was developed and used for fitting was implemented in MATLAB/Simulink version 2014b (The MathWorks, Inc., Natick, Massachusetts, United States). All calculation steps were conducted employing one Simulink model, i.e. the PBPK model was combined with additional mathematical operations required for BED calculations.

#### From pharmacokinetics to BED

The BED was calculated to take into account the dose rate and radiobiological parameters [32]. The pharmacokinetics of labeled and unlabeled peptide, the fraction of administered activity *a*_*i*_(*t*), the corresponding time-activity curve A_i,_(t) in each organ i and the absorbed dose rate ${\overset{•}{D}}_{i}(t)$ were simulated.

For salivary glands, tumor and kidney only the self-doses were considered according to

$${\overset{•}{D}}_{i}(t)=A_{i}(t)\cdot S_{i\leftarrow i}=A_{inj}\cdot a_{i}(t)\cdot S_{i\leftarrow i}$$ (1)

where A_inj_ is the injected activity and S_ii_ represents the self-absorbed dose rate per unit activity of organ i.

For red marrow the self-dose and the irradiation from the remainder were considered [33] according to

$${\overset{•}{D}}_{RM}{}_{\;}=A_{RM}(t)\cdot S_{RM\leftarrow RM}+A_{REM}(t)\cdot S_{RM\leftarrow REM}$$ (2)

where S_RMRM_ represents the self-absorbed dose rate per unit activity of red marrow and S_RMREM_ the absorbed dose rate per unit activity of remainder to red marrow. A_RM_(t) and A_REM_(t) are the time-activity curves of the red marrow and the remainder.

The absorbed dose was determined by numerical integration according to

$$D_{i}{\left(T\right)}_{\;}=\int_{0}^{T}\overset{•}{D_{i}}(t)dt$$ (3)

with T = 3·10^4^ min. The BED of all relevant organs was calculated using the absorbed dose and the Lea–Catcheside factor G_i,_ (Eq. 17, S1 File) [32] of each individual dose rate according to

$$BED_{i}{{}_{}}_{\;}=D_{i}\cdot (1+\frac{G_{i}}{\alpha_{i}/\beta_{i}}\cdot D_{i})$$ (4)

#### Maximal tolerable doses

For PRRT with ^90^Y a BED-effect relationship for the kidney could be established [34]. Although several dosimetry studies with two ^177^Lu-PSMA compounds have been conducted, for this therapy maximal tolerable doses are still unknown and the optimal number of cycles has not been identified [35]. Here, we assumed 4 therapy cycles having equal biodistributions. We further assumed a maximal cumulative kidney BED of 40 Gy_2.5_ [36] based on data from PRRT with ^90^Y. A maximal cumulative salivary absorbed dose of 30 Gy from experiences in radiation therapy, which was used in a recent initial study for PSMA-617 [6], was also employed here. Usually, for the red marrow 2 Gy are assumed, however, due to possible irradiation from bone metastases (which were here included in the remainder), the value was reduced to 1 Gy_15_. Therefore, the maximal kidney BED for one cycle was set to 10 Gy_2.5_. The absorbed dose for salivary gland was set to 7.5 Gy and for red marrow BED to 0.25 Gy_15_. For salivary glands no reliable value for a maximal tolerable BED could be found in the literature, therefore, instead of BED the absorbed dose was used as boundary condition. Nevertheless, the BEDs of the salivary glands were calculated as all needed information is available.

#### Absorbed dose rates per unit activity (S-values)

S-values [37] and radiobiological parameters α/β and μ [32, 38–41] were taken from the literature (S1 File). The red marrow S-values were determined individually for each patient, i.e. the red marrow to red marrow S-value was scaled using body weight, the remainder to red marrow S-value was corrected according to Eqs 6, 13 and 22 in Hindorf et al. [33]. For tumor and salivary gland, spherical shapes were assumed [37]. For each individual volume of the salivary glands and tumor, the S-values were either taken directly from OLINDA [37] or derived by logarithmic interpolation. For metastases the same radiobiological parameters as for primary prostate cancer were used [40]. As there is an open discussion regarding the relevance of the salivary glands as a dose limiting organ, both scenarios were investigated, i.e. kidneys *or* salivary glands dose limiting and kidney *and* salivary glands dose limiting.

#### Activities to administer, absorbed doses and BEDs

In the first step all allowed combinations are identified, i.e. iso-dose curves for kidneys and salivary glands were generated. Simulations with smaller and larger than usually applied therapeutic amounts were used to demonstrate the effect for a wide range [11]. Activities to administer were calculated for amounts of 2−2^13^ nmol (in steps of factor 2) and the constraints (for one cycle): kidney BED of 10 Gy_2.5_ (case 1) *or* salivary glands absorbed dose of 7.5 Gy (case 2).

For kidneys and each amount j (case 1):

$$\begin{array}{l} A_{inj\;1,j}=\frac{-{\tilde{a}}_{K,j}(T)+\sqrt{{\tilde{a}}_{K,j}{\left(T\right)}_{}^{2}+\frac{8\int_{0}^{T}a_{K,j}(t)dt\cdot \int_{0}^{t}a_{K,j}(\omega )\cdot e^{-\mu_{K}(t-\omega )}d\omega \cdot BED_{K,fixed}}{\alpha_{K}/\beta_{K}}}}{\frac{4\cdot S_{K\leftarrow K}\int_{0}^{T}a_{K,j}(t)dt\cdot \int_{0}^{t}a_{K,j}(\omega )\cdot e^{-\mu_{K}(t-\omega )}d\omega }{\alpha_{K}/\beta_{K}}} \end{array}$$ (5)

Eq 5 is derived in S1 File pages 9–10. A_inj1,j_ is the activity to administer leading to a kidney BED of 10 Gy_2.5_, ${\tilde{a}}_{K,j}(T)\;$ is the time-integrated activity coefficient, *a*_*K*,*j*_(*t*) the fraction of administered activity in the kidneys for each amount j and *BED*_*K*,*fixed*_ the maximal tolerable biologically effective dose (10 Gy_2.5_) and *S*_*K*←*K*_ the self-absorbed dose rate per unit activity of the kidneys.

For salivary glands (Eq.21, S1 File) and each amount (case 2):

$$A_{inj\;2,j}{=}_{\;}\frac{D_{SAL,fixed}}{{\tilde{a}}_{SAL,j}(T)\cdot S_{SAL\leftarrow SAL}}$$ (6)

where ${\tilde{a}}_{SAL,j}(T)\;$ is the time-integrated activity coefficient for each amount j, *D*_*SAL*,*fixed*_ the maximal tolerable absorbed dose and *S*_*SAL*←*SAL*_ the self-absorbed dose rate per unit activity of the salivary glands.

The absorbed doses and BEDs for all organs were then calculated based on Eqs 1–4 above for the amounts j and maximum activities to administer.

#### Optimal tumor dose

In a subsequent step, all amounts and pertaining activities, which led to red marrow BED > 0.25 Gy_15,_ were excluded as the red marrow is considered dose limiting. Thus, only for amounts and pertaining activities, which led to a red marrow BED < 0.25 Gy_15_, the optimal tumor dose considering the kidney (case 1) *or* the salivary glands (case 2) as dose limiting organ was identified.

#### Comparing optimal and actually used amounts

To consider the kidneys *and* the salivary glands as dose limiting organ and to account for radiochemical limitations (maximal possible specific activity), a new set of combinations was assembled using the activities from case 1 and case 2 for each amount from 2^5^−2^9^ nmol. All amounts and pertaining activities, which led to red marrow BED > 0.25 Gy_15,_ were excluded. From the remaining combinations, the optimal tumor dose was identified and compared to the “actual” tumor dose. The actual tumor dose was determined using the actual amount and an activity that would have led to a salivary gland absorbed dose of 7.5 Gy and kidney BED < 10 Gy_2.5_ or kidney BED of 10 Gy_2.5_ and absorbed dose < 7.5 Gy.

#### Tumor perfusion sensitivity analysis

A sensitivity analysis regarding the influence of the assumption for tumor perfusion was conducted. Perfusion is an important parameter for small molecules. It had to be fixed in the fitting process due to high correlation with the binding site density. Four different values for the perfusion rates 0.01, 0.1, 0.5 and 1 ml/g/min were investigated. The models were fitted to the data of each individual patient and compared using the Akaike weights based on the AIC [29, 30]. Subsequently, simulations as described above were conducted for models with Akaike weight >1%.

## Results

### Fitting and validation

**Fig 2:**
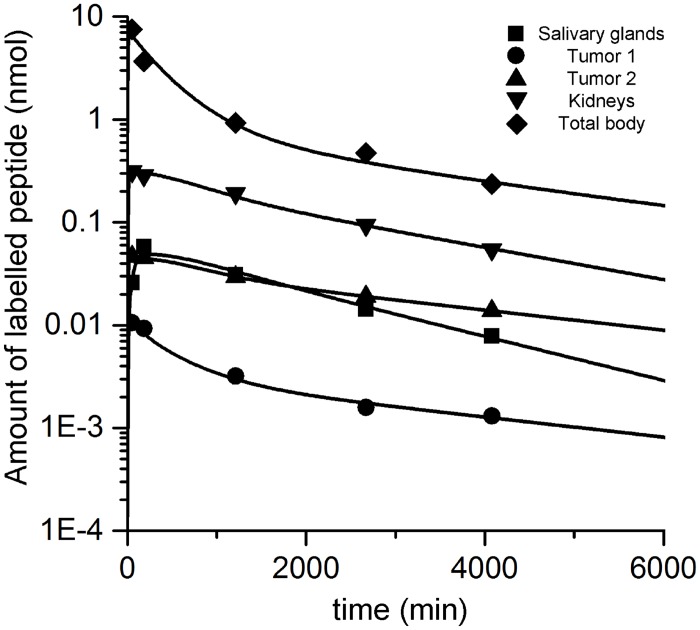
Typical fit. Typical fit (patient 2) of normal tissue and tumor.

Visual inspection showed good fits. The coefficient of determination R^2^ was > 0.85 for all curves. A typical fit is depicted in Fig 2. All fits yielded coefficients of variation (relative standard errors) < 50% for any estimated parameter, except for the estimated tumor ROI background correction fraction c_2_ (60%) for P5 (S2 File). Elements of the correlation matrix were < 0.8 except for the tumor release rate and binding site density of P1 (0.85), P2 (0.86) and P6 (0.84) and the salivary glands release rate and binding site density for P2 (0.82) [31]. Model 1 was more supported by the data: for all patients the Akaike weights were > 0.9. Thus, only the estimated parameters of model 1 were further used. The values of the estimated physiological parameters (Table 2) compare favorably to literature values, i.e., they are in a physiologically reasonable range [11, 14, 16, 42]. The results of each individual patient are presented in S2. The estimated fractional standard deviations for the tumor, salivary glands and the kidney data sets for P1-5 were 3–25%, 4–15% and 5–27%, respectively.

### Simulations

**Fig 3:**
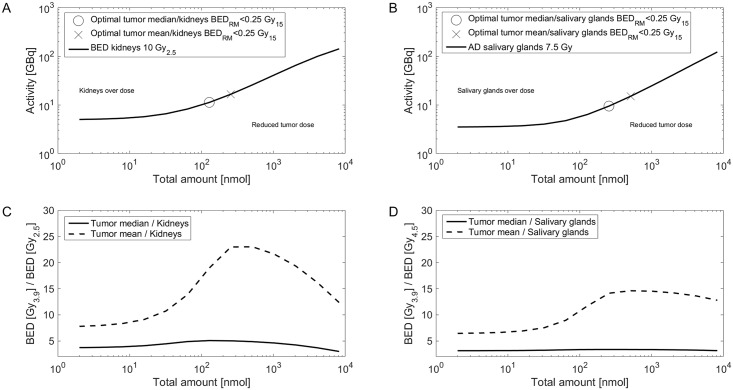
Optimal BED ratios. In panel A and B, the activity and peptide amount combinations that lead to a kidney BED of 10 Gy_2.5_ (A) or a salivary absorbed dose of 7.5 Gy (B) for red marrow BED < 0.25 Gy_15_ are depicted for the median patient. All combinations along the kidney 10 Gy_2.5_ line (C) or salivary glands 7.5 Gy (D) are used to calculate the tumor BEDs for mean and median tumor parameters. The optimal combinations for a maximal tumor-to-kidney or tumor-to-salivary gland BED ratio were added to panel A and B, respectively. The simulations of each single patient are shown in S2.

**Fig 4:**
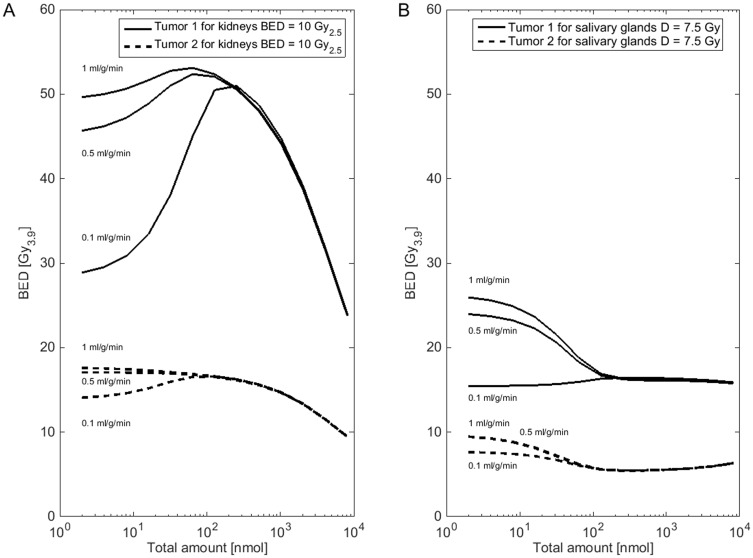
Blood flow sensitivity analysis. In panel A and B, the tumor BED for activity and peptide amount combinations (2−2^13^ nmol and red marrow BED < 0.25 Gy_15_) that lead to a kidney BED of 10 Gy_2.5_ (A) or salivary gland BED of 7.5 Gy (B) is shown for different assumptions for the perfusion rate. The estimated parameters of patient 2 (bone tumor 1, lymph node tumor 2) were used. The results of all patients can be found in S3 File.

Simulations for the median normal tissue parameters and mean/median tumor parameters (Table 2) are depicted in Fig 3. The graphs for each individual patient are presented in S2. The optimal amount and pertaining activities for kidney BED of 10 Gy_2.5_ *or* salivary absorbed dose of 7.5 Gy (for red marrow BED < 0.25 Gy_15_) and the corresponding tumor absorbed doses and BEDs are provided in Table 3. Considering solely the kidneys (case 1) or salivary glands (case 2) as dose limiting organ, the median optimal amount and pertaining activity was 192 nmol (range: 2–512 nmol) and 9.3 GBq (range: 7.9–22.5 GBq) and 257 nmol (range: 2–2048 nmol) and 4.9 GBq (range: 2.4–16.5 GBq), respectively. The *maximal possible* tumor-to-kidneys BED ratio depends predominately on the ratio of binding site density (for a given organ volume). In addition, higher interstitial and serum volumes and unspecific uptake in the kidneys reduce the *maximal possible* tumor-to-kidneys BED ratio. The *actual* BED ratio (achieved in actual clinical scenario) predominately depends on the ratio of perfusion (see Fig 4), binding site density and the actually administered amount. With higher amounts also unspecific uptake (although assumed to be low) becomes more important. In addition, for higher amounts serum and interstitial free peptide are more relevant. For lower amounts the perfusion is more important. Therefore, the *optimal amount* depends on the perfusion, binding site density, serum volume and interstitial volume ratios and on unspecific kidney uptake. The same holds true for salivary glands besides the unspecific uptake.

**Table 3:** Optimal amounts (for 2−2^13^ nmol), pertaining activities, absorbed doses and biologically effective doses (BED).

		Individual optimal amount^a^ and individual activity for 10 Gy_2.5_ kidney BED^b^ and RM BED < 0.25 Gy_15_						Individual optimal amount and individual activity for 7.5 Gy salivary gland D^b^ and RM BED < 0.25 Gy_15_					
		Injection		Tumor		Salivary glands		Injection		Tumor		Kidneys	
Patient	Tumor lesion	Activity[GBq]	Amount [nmol]	D[Gy]	BED[Gy_3.9_]	D[Gy]	BED[Gy_4.5_]	Activity[GBq]	Amount [nmol]	D[Gy]	BED[Gy_3.9_]	D[Gy]	BED [Gy_2.5_]
P1	1	22.5	512	156	234	10	11	16.5	512	114	156	5.7	6.9
	2	22.5	512	390^c^	850^c^	10	11	16.5	512	285	530	5.7	6.9
P2	1	13.9	64	46	52	20	24	4.4	2	23	24	4.3	4.9
	2	8.0	2	16	17	14	16	4.4	2	9.0	9.2	4.3	4.9
P3	1	14.1	256	171	223	40	53	4.3	512	33	35	1.6	1.6
	2	9.4	128	46	50	40	54	12.3	2048	8.6	8.7	1.8	1.9
P4	1	7.9	64	24	26	8.9	9.7	4.9	2	23	24	8.3	11
	2	7.9	64	14	14	8.9	9.7	4.9	2	13	14	8.3	11
P5	1	9.3	256	29	32	14	15	2.4	2	17	18	6.0	7.4
	2	9.3	256	42	47	14	15	8.2	512	23	25	4.4	5.1
Mean		12.5	211	93	154	18	22	7.9	411	55	84	5	6
SD		5.8	183	118	258	12	17	5.3	629	87	163	2	3
Median		9.3	192	44	48	14	15	4.9	257	23	24	5	6
Min		7.9	2	14	14	8.9	9.7	2.4	2	9	9	1.6	1.6
Max		22.5	512	390	850	40	54	16.5	2048	285	530	8	11

Abbreviations: D absorbed dose, BED biologically effective dose

^a^1.66 μg of the ligand corresponds to 1 nmol. For these calculations, radiochemical limitations are not considered.

^b^ Kidney BED of 10 Gy_2.5_ corresponds to a D of 7.9±0.2 Gy and salivary gland D of 7.5 Gy to a BED of 8.1±0.1 Gy_4.5_

^c^The simulated absorbed dose is high due to high tumor binding site concentration (2412 ± 802 nmol/l) and large injected activity. Therefore, the BED calculated using the linear quadratic model becomes extremely large. The linear quadratic model is probably not applicable for such large doses.

In Table 4, for amounts of 32–512 nmol the ratio of the optimal to actual tumor BED, considering maximal tolerable absorbed or effective doses for kidneys *and* salivary glands and red marrow is presented. The potential improvement varied substantially (factor of 1.0–3.0) between patients and tumor lesions.

**Table 4:** Ratio of BED for actually and optimal amounts (for 32–512 nmol) and activity.

		Actual		Optimal^a^		D_TUoptimal_/D_TUactual_ [unity]	BED_TUoptimal_/BED_TUactual_ [unity]
Patient	Tumor	Activity [GBq]	Amount [nmol]	Activity [GBq]	Amount [nmol]		
P1	1	6.0	148	16.5	512	1.3	1.4
	2			16.5	512	2.2	3.0
P2	1	5.4	98	4.7	32	1.2	1.2
	2			4.7	32	1.2	1.2
P3	1	5.4	89	4.3	512	1.2	1.2
	2			4.3	512	1.0	1.0
P4	1	5.4	74	5.6	32	1.1	1.1
	2			5.6	32	1.1	1.1
P5	1	5.6	302	2.5	32	1.0	1.0
	2			8.2	512	1.0	1.0

Abbreviations: D absorbed dose, BED biologically effective dose

^a^Considering kidney BED ≤ 10 Gy, salivary glands AD ≤ 7.5 Gy and RM BED < 0.25 Gy. Amounts of peptide are limited to 32–512 nmol

#### Tumor perfusion sensitivity analysis

For all patients, the assumption of tumor perfusion rate 0.01 ml·g^-1^·min^-1^ was not supported by the data (Akaike weights < 1%). In 3 patients, perfusion rates for 0.1, 0.5 and 1 ml·g^-1^·min^-1^ yielded acceptable results (Table 2, S3 File). For all patients, the average Akaike weights for the assumptions of 0.1, 0.5 and 1 were 19%, 33% and 47%, respectively. Using the weights, an average rate of 0.66 ml·g^-1^·min^-1^ was calculated. This value is close to the value [27] that was taken from the literature (S1 File) for primary tumors. These assumptions led to the same time–integrated-activity-coefficient, i.e., when fitting lower fixed perfusion rates were compensated by higher binding site densities. Fig 4 shows for patient 2 how the chosen perfusion rate affects the predicted tumor BED for other amounts and activities. For amounts, as they were actually used, the tumor BED is less sensitive to the chosen perfusion rate than for smaller amounts. The differences of the predicted tumor BED for the optimal amount (for 32–512 nmol) and activity, considering kidney *and* salivary glands *and* red marrow as dose limiting, ranged from 0.6–20%.

## Discussion

To investigate the effect of peptide and activity amount on the tumor-to-kidney and tumor-to-salivary glands BED ratio in ^177^Lu-labeled PSMA-targeted therapy, we applied a recently developed (and herein modified) modeling approach [11] that allows optimizing the PSMA ligand amount and activity taking into account maximum tolerable absorbed doses for the salivary glands and biologically effective doses for the kidneys and red marrow.

The PBPK model described the data well regarding visual inspection, coefficients of determination and variation and correlation of parameters. In general, the estimated parameters were in a physiologically reasonable range. The estimated ratio of PSMA expression of tumor-to-kidney or tumor-to-salivary gland is lower than reported in the literature [5, 43]. However, the mean value (~230.000 per cell) and the range (~12.000 to ~1.5∙10^6^ per cell) of estimated PSMA expression rates of the investigated tumors compare favorably with in vitro experiments for various cell lines and different PSMA specific ligands [14–16] assuming 10^12^ cells/l tumor (Table 2). Therefore, the higher than expected estimated PSMA expression rates might also include/mimic unspecific binding, which was neglected (in the model) in the salivary glands and was assumed low in the kidneys [9]. The kidney pharmacokinetic model structure and parameters for unspecific uptake were adopted from [11] assuming similar effective blocking of unspecific uptake using amino acids. The importance of non-PSMA specific uptake in the kidneys and salivary glands has to be elucidated in further work.

The sensitivity analysis of the tumor perfusion rate showed that the choice of the fixed values, if preselected with the AICc, had no great effect on the tumor BED, if the optimal amount is large. However, the extrapolation to smaller amounts led to considerably different results (Fig 4). In the light of the attempt of some working groups to use PET for pre-therapeutic dosimetry, extrapolation from measurements using small amounts of peptide to therapy with substantially higher amounts should be further investigated [44].

The performed simulations showed that for a fixed ratio of tumor-to-kidney blood flow rates (here ~ 1/5) the ratio of tumor-to-kidneys binding site density (here ~ 1.4–77) is the most important quantity that determines the location of the optimum (optimal amount) if the kidney is considered the limiting organ. In addition, the binding site density ratio is also the most important quantity that determines the *maximal possible* BED ratio. Unspecific kidney uptake (although assumed to be low) and the tumor-to-kidney ratio of interstitial and serum volume fractions become more relevant with larger administered amounts. The *actual* BED ratio is therefore predominantly determined by the actual number of bound ligand per organ mass. However, high ligand density necessitates both, a high binding site density and high binding site occupancy, i.e. high degree of saturation. The kidney binding sites are saturated with considerably lower amounts compared to most tumors due to the high kidney perfusion (~2.5 ml·g^-1^·min^-1^) and medium (mean 27 nmol·L^-1^) binding site density. Therefore, for increasing amounts, the fraction bound or internalized peptide of the total applied peptide amount (and therefore the fraction of activity, as labeled and unlabeled are assumed to behave equally) decreases stronger in the kidney than in the tumor. Thus, compared to the kidney, the time-integrated activity coefficient of the tumor declines slower or even stays constant with increasing amounts depending on tumor perfusion and binding site density [9]. Higher amounts of activity are allowed for lower time-integrated activity coefficients in the kidney. This leads to an improved BED ratio for higher administered peptide amounts. For the investigated patients, the perfusion rates and binding site densities of the salivary glands and the tumor were more similar than for the kidneys and tumor. Therefore, the tumor-to-salivary glands BED ratio for was more similar for all amounts than for the kidney-to-tumor BED ratio.

In general, the simulations showed that the higher the ratio of tumor-to-kidney binding site density, the larger the effect of the amount of peptide on the BED ratio. In many cases, amounts, which are normally used for PET imaging (~ 2−2^3^ nmol), led to considerably different tumor-to-kidney ratios than amounts used for therapy (~ 2^6^−2^8^ nmol) (Fig 3). However, for the investigated patients the therapeutic window was not specifically narrow and the actually injected amount close to the optimum (Table 4). Therefore, the potential improvement (actual versus optimal) was small (factor 1–1.2) in 4 out of 5 patients. However, for lesions with high binding site density (e.g. 2412 ± 802 nmol/l in patient 1) larger amounts and activities would have been beneficial according to the presented simulations (tumor BED shows a 3 fold increase). The sensitivity analysis for the tumor perfusion rate also showed that larger amounts and activities are an option to increase the ratio for less well perfused tumors with average binding site densities. More data are required to identify whether a standard amount is sufficient for most patients or whether an individualization of the optimal amount is necessary. In both cases, however, the pertaining activity has to be determined depending on the number of intended cycles and the limit to the kidney and salivary glands.

Although we believe, due to the given physiological model structure and *a priori* knowledge, the estimated parameter values and simulations are a good approximation of the real physiological processes, pharmacokinetic data derived from 3D imaging techniques are required to further validate the method and to improve the predication accuracy. For example, a more accurate determination of organ and tumor perfusion necessitates early 3D measurements. For the release rates, 2D measurements might be sufficient.

Even in case of perfect biokinetic data (no error, infinite number of measurements) and a perfect model (extrapolation from fitted curves to simulated TIACs correct), there are potential sources for errors. Inaccurate estimation of tumor and organ volume might lead to an error in the determined absorbed dose. The radiobiological parameters or the structure of BED model itself might not apply for high doses (Table 3, patient 1). In addition, it is not entirely clear which tolerable doses can be applied for this specific application. The absorbed dose instead of the BED for salivary glands was used as to our knowledge no general used BED tolerable dose is available for molecular radiotherapy. Using constrains for kidney (BED of 10 Gy_2.5_) and for salivary glands (absorbed dose of 7.5 Gy), the salivary gland was the dose limiting organ in all patients for amounts of 32–512 nmol.

For the presented data, if higher tolerable absorbed doses to the salivary glands are assumed, optimizing the amount and activity becomes more important. The influence of a different number of cycles or different maximal tolerable doses on the tumor-to-kidney or salivary gland ratio and on the optimal amount is object to further investigations.

## Conclusions

Based on PBPK model simulations, the effect of the peptide amount on the tumor-to-kidney BED ratio and the tumor-to-salivary glands BED ratio predominantly depends on the binding site density ratio and the ratio of perfusions (blood flow per mass). With increasing amounts of peptide the influence of the perfusion decreases and the serum volume and interstitial volume ratios become more important. For the kidneys also the unspecific kidney uptake is increasingly important for larger amounts. For therapy, the currently given amounts of approximately 100–300 nmol yield favorable BED ratios for many lesions. However, there is potential in individually optimizing the amount of peptide and pertaining activity for tumors with high PSMA binding site densities relative to kidney and salivary glands. In addition, the BED of less well perfused tumors (<0.1 ml∙g^-1^∙min^-1^) with average binding site densities could be increased by using larger amounts and pertaining activities. The validation of the presented model and method is ongoing.

## Supporting Information

S1 File Equations, parameters and compartments. All differential equations and the compartmental model structure with the pertaining parameters and parameter values as well as the equations describing BED calculations.
(DOC)

S2 File Fitting results and simulations. Estimated parameters, fitted curves as well as the pertaining BED simulations for all patients.
(PPTX)

S3 File Tumor perfusion analysis. BED simulations for all patients with varying blood flows to the tumor.
(PPTX)
