# Supplementary material for: Investigating the Effect of Ligand Amount and Injected Therapeutic Activity: A Simulation Study for 177Lu-Labeled PSMA-Targeting Peptides
Source: PLoS One. 2016 Sep 9;11(9):e0162303. doi: 10.1371/journal.pone.0162303 (PMC5017739; doi:10.1371/journal.pone.0162303)
Supplement: S1 File — All differential equations and the compartmental model structure with the pertaining parameters and parameter values as well as the equations describing BED calculations. (DOC) [file pone.0162303.s001.doc]

**S1:** **Equations, parameters and compartments**

**PBPK Model equations**

The following equations describe the transport of labeled (indexed with *) and unlabeled peptide via blood flow, extravasation, binding, internalization, degradation and release, excretion and radioactive decay. For therapy the peptide was intravenously injected as a 10 min infusion. In model 1 and model 2 the dissociation rate is different, the model structure and all other fixed parameters are identical.

The variables are defined in Table A.

**Bound and internalized peptide:**

Tumor, salivary glands, kidneys, liver, spleen, GI and prostate:

Constraint for total PSMA binding sites R_0,i_

$R_{0, i}=R_{i}+RP_{\text{ }i}+RP_{\text{ }i}^{\text{*}}$ (1)

Internalized peptide

(2)

Bound peptide on cell surface

$\begin{matrix} \frac{d}{dt}RP_{\text{ }i}=k_{on}\cdot P_{\text{ }i,\mathrm{int}}\cdot\frac{R_{i}}{V_{i,\mathrm{int}}}-(k_{off}+\lambda_{\mathrm{int},i})\cdot RP_{\text{ }i}+\lambda_{phy}\cdot RP_{\text{ }i}^{\text{*}}\text{ } \\ \frac{d}{dt}RP_{\text{ }i}^{*}=k_{on}\cdot P_{\text{ }i,\mathrm{int}}^{*}\cdot\frac{R_{i}}{V_{i,\mathrm{int}}}-(k_{off}+\lambda_{\mathrm{int},i})\cdot RP_{\text{ }i}^{*}-\lambda_{phy}\cdot RP_{\text{ }i}^{\text{*}} \end{matrix}$ (3)

**Free peptide, vascular:**

Transcapillary extravasation is described by the permeability surface product (PS_i_) and the vascular (V_i,v_) and interstitial volumes (V_i,int_) of the pertaining tissue. Convection from the vascular to the interstitial space is neglected as the used peptide represents a rather small molecule .

All tissues except kidneys and lungs

(4)

For brain PS = 0

Lungs

$\begin{matrix} \frac{d}{dt}P_{\text{ }L\text{U},v}=PS_{LU}\left(\frac{P_{\text{ }L\text{U},\mathrm{int}}}{V_{LU,\mathrm{int}}}-\frac{P_{\text{ }L\text{U},v}}{V_{LU,v}}\right)+F\left(\frac{P_{\text{ }V\text{EN}}}{V_{VEN}}-\frac{P_{\text{ }L\text{U},v}}{V_{LU,v}}\right)+\lambda_{phy}\cdot P_{\text{ }L\text{U},v}^{\text{*}} \\ \frac{d}{dt}P_{\text{ }L\text{U},v}^{*}= PS_{LU}\left(\frac{P_{\text{ }L\text{U},\mathrm{int}}^{*}}{V_{LU,\mathrm{int}}}-\frac{P_{\text{ }L\text{U},v}^{*}}{V_{LU,v}}\right)+F\left(\frac{P_{\text{ }V\text{EN}}}{V_{VEN}}-\frac{P_{\text{ }L\text{U},v}^{*}}{V_{LU,v}}\right)-\lambda_{phy}\cdot P_{\text{ }L\text{U},v}^{\text{*}}\text{ } \end{matrix}$ (5)

Kidneys

(6)

Veins

(7)

Arteries

$\begin{matrix} \frac{d}{dt}P_{\text{ }A\text{RT}}=-\sum\frac{F_{i}}{V_{ART}}\cdot P_{\text{ }i\text{,v}}+\frac{F}{V_{LU,v}}\cdot P_{\text{ }L\text{U,v}}+\lambda_{phy}\cdot P_{ART}^{*} \\ \frac{d}{dt}P_{ART}^{*}=-\sum\frac{F_{i}}{V_{ART}}\cdot P_{\text{ }i\text{,v}}+\frac{F}{V_{LU,v}}\cdot P_{\text{ }L\text{U,v}}-\lambda_{phy}\cdot P_{ART}^{*} \end{matrix}$ (8)

**Free peptide, interstitial spaces:**

Kidneys:

$\begin{matrix} \frac{d}{dt}P_{\text{ }K,\mathrm{int}}=-k_{on}\cdot P_{\text{ }K,\mathrm{int}}\cdot\frac{R_{K}}{V_{K,\mathrm{int}}}+k_{off}\cdot RP_{K}+F_{fil}\left(\frac{P_{\text{ }K,v}}{V_{K,v}}-\frac{P_{\text{ }K,\mathrm{int}}}{V_{K,\mathrm{int}}}\right)+\lambda_{phy}\cdot P_{\text{ }K,\mathrm{int}}^{\text{*}} \\ \frac{d}{dt}P_{\text{ }K,\mathrm{int}}^{\text{*}}=-k_{on}\cdot P_{\text{ }K,\mathrm{int}}^{\text{*}}\cdot\frac{R_{K}}{V_{K,\mathrm{int}}}+k_{off}\cdot RP_{K}^{*}+F_{fil}\left(\frac{P_{\text{ }K,v}^{\text{*}}}{V_{K,v}}-\frac{P_{\text{ }K,\mathrm{int}}^{\text{*}}}{V_{K,\mathrm{int}}}\right)-\lambda_{phy}\cdot P_{\text{ }K,\mathrm{int}}^{\text{*}} \end{matrix}$ (9)

Muscle, red marrow, skin, lungs, adipose tissue, heart, bone, rest and brain (PS = 0):

$\begin{matrix} \frac{d}{dt}P_{\text{ }i,\mathrm{int}}=PS_{i}\left(\frac{P_{\text{ }i,v}}{V_{i,v}}-\frac{P_{\text{ }i,\mathrm{int}}}{V_{i,\mathrm{int}}}\right)+\lambda_{phy}\cdot P_{\text{ }i,\mathrm{int}}^{\text{*}} \\ \frac{d}{dt}P_{\text{ }i,\mathrm{int}}^{\text{*}}=PS_{i}\left(\frac{P_{\text{ }i,v}^{\text{*}}}{V_{i,v}}-\frac{P_{\text{ }i,\mathrm{int}}^{\text{*}}}{V_{i,\mathrm{int}}}\right)-\lambda_{phy}\cdot P_{\text{ }i,\mathrm{int}}^{\text{*}}\text{ } \end{matrix}$ (10)

Tumor, salivary glands, kidneys, liver, spleen, GI and prostate:

$\begin{matrix} \frac{d}{dt}P_{\text{ }i,\mathrm{int}}=-k_{on}\cdot P_{\text{ }i,\mathrm{int}}\cdot\frac{R_{i}}{V_{i,\mathrm{int}}}+k_{off}\cdot RP_{i}+PS_{i}\left(\frac{P_{\text{ }i,v}}{V_{i,v}}-\frac{P_{\text{ }i,\mathrm{int}}}{V_{i,\mathrm{int}}}\right)+\lambda_{phy}\cdot P_{\text{ }i,\mathrm{int}}^{\text{*}} \\ \frac{d}{dt}P_{\text{ }i,\mathrm{int}}^{\text{*}}=-k_{on}\cdot P_{\text{ }i,\mathrm{int}}^{\text{*}}\cdot\frac{R_{i}}{V_{i,\mathrm{int}}}+k_{off}\cdot RP_{\text{ }i}^{*}+PS_{i}\left(\frac{P_{\text{ }i,v}^{\text{*}}}{V_{i,v}}-\frac{P_{\text{ }i,\mathrm{int}}^{\text{*}}}{V_{i,\mathrm{int}}}\right)-\lambda_{phy}\cdot P_{\text{ }i,\mathrm{int}}^{\text{*}} \end{matrix}$ (11)

**Further equations:**

Peptide in kidney cells (unspecific)

(12)

Bound to protein

$\begin{matrix} \frac{d}{dt}PRP_{\text{ }}=k_{PR}\cdot P_{\text{ }V\text{EN}}+\lambda_{phy}\cdot PRP^{*} \\ \frac{d}{dt}PRP^{*}=k_{PR}\cdot P_{VEN}^{*}-\lambda_{phy}\cdot PRP^{*} \end{matrix}$ (13)

**TABLE A Parameter definition**

| **Variable** | | **Value** | **Unit** | **Source** |
| --- | --- | --- | --- | --- |
| k_on_ | association rate | 0.046 | l·nmol^-1^·min^-1^ | a |
| K_D_ | dissociation constant | 1 or 8 | nmol·l^-1^ | a |
| k_off_ | dissociation rate | K_D_· k_on_ | min^-1^ |  |
| λ_phy_ | physical decay ^177^Lu | 7.15·10^-5^ | min^-1^ |  |
| BW | body weight | measured | kg |  |
| BH | body height | measured | cm |  |
| H | hematocrit | measured | unity |  |
| F | flow total serum | V_P_ ·1.23/min^b^ | l·min^-1^ |  |
| V_P_ | volume of total body serum | 2.8·(1- H)·BSA·(l·m^-2^) | l |  |
| BSA | body surface area | 0.007184·BH^0.725^·BW^0.425^ | m^2^ |  |
| ρ | assumed density for all organs and tumor | 1 ml ≙ 1 g |  |  |
|  |  |  |  |  |
| **Tumor** | | | | |
| V_TU,total_ | total volume of tumor 1 and 2 | measured | l |  |
| V_TU, int_ | interstitial space of tumor | v_TU,int_·V_TU,total_ | l |  |
| V_TU, v_ | vascular space of tumor | v_TU,v_·V_TU,total_ | l |  |
| V_TU,Rest, int_ | interstitial space of tumor remainder | v_TU,int_·R_TU,Rest, 0_/[R_TU,Rest, 0_] | l |  |
| V_TU,Rest, v_ | vascular space of tumor remainder | v_TU,v_·R_TU,Rest, 0_/[R_TU,Rest, 0_] | l |  |
| v_TU,int_ | interstitial space fraction of total tumor | 0.38 | unity |  |
| v_TU,v_ | vascular (serum) fraction of total tumor | 0.05·(1-H) | unity |  |
| F_TU_ | serum flow tumor | f_TU_·V_TU,total_ | l·min^-1^ |  |
| F_TU,Rest_ | serum flow tumor remainder | f_TU_·R_TU,Rest, 0_/[R_TU,Rest, 0_] | ml·min^-1^ |  |
| f_TU_ | serum flow density tumor | 0.5 | ml·min^-1^·g^-1^ |  |
| PS_TU_ | permeability surface area product tumor | k_TU_·V_TU,total_ | ml·min^-1^ |  |
| PS_TU,Rest_ | permeability surface area product tumor remainder | k_TU_·R_TU,Rest, 0_/[R_TU,Rest, 0_] | ml·min^-1^ |  |
| k_TU_ | permeability surface area product per unit mass (scaled for molecule size of PSMA I&T) | 0.6 (maximal value from (6)) | ml·min^-1^·g^-1^ |  |
| [R_TU,0_] | PSMA binding site density | fitted | nmol·l^-1^ |  |
| [R_TU,Rest, 0_] | PSMA binding site density tumor remainder | 266 | nmol·l^-1^ |  |
| R_TU,0_ | PSMA binding sites number | [R_TU,0_]·V_TU,total_ | nmol |  |
| R_TU,Rest, 0_ | PSMA binding sites number tumor remainder ^c^ | P1-5: 13 ; 2.6 ;13 ; 2.6 ;13 | nmol |  |
| λ_TU,int_ | internalisation rate tumor | 0.001 | min^-1^ |  |
| λ_TU,release_ | release rate tumor | fitted | min^-1^ |  |
| λ_TU,Rest,int_ | internalisation rate tumor remainder | 0.001 | min^-1^ |  |
| λ_TU,Rest,release_ | release rate tumor remainder | (λ_TU,1,release_ + λ_TU,2,release_)/2 | min^-1^ |  |
|  |  |  |  |  |
| **Liver , spleen and kidneys** | | | | |
| V_L,total_ | volume total liver | 1.91 | l |  |
| V_S,total_ | volume total spleen | 0.183 | l |  |
| V_K,total_ | volume total kidneys | measured | l |  |
| V_i,v_ | vascular (serum) volume organ liver, spleen, kidneys | V_i,total_·v_i,v_ | l |  |
| V_i,int_ | interstitial volume liver, spleen, kidneys | V_i,total_·v_i,int_ | l |  |
| V_K, intra_ | volume intracellular kidneys | (V_K,total_ - V_K,int_ - V_K,v_)·2/3^d^ | l |  |
| v_L,v_ | vascular (serum) fraction liver | 0.085 | unity |  |
| v_S,v_ | vascular (serum) fraction spleen | 0.12 | unity |  |
| v_K,v_ | vascular (serum) fraction kidneys | 0.055 | unity |  |
| v_L,int_ | interstitial fraction liver | 0.2 | unity |  |
| v_S,int_ | interstitial fraction spleen | 0.2 | unity |  |
| v_K,int_ | interstitial fraction kidneys | 0.15 | unity |  |
| F_L_ | serum flow liver arterial | 0.065·F | l·min^-1^ |  |
| F_S_ | serum flow spleen | 0.03·F | l·min^-1^ |  |
| F_K_ | serum flow kidneys | 0.19·F | l·min^-1^ |  |
| φ | ratio of sieving coefficients | Θ_PSMA I&T_ / Θ_Cr-51-EDTA_= 0.66 | unity |  |
| GFR | glomerular filtration rate with ^51^Cr-EDTA | TER_measured_/3·20 l /15 l | l·min^-1^ |  |
| TER | tubular extraction rate ^99m^Tc-MAG3 | measured | l·min^-1^ |  |
| F_fil_ | filtration | GFR·φ ^e^ | l·min^-1^ |  |
| F_ex_ | excretion | F_fil_·f_ex_ | l·min^-1^ |  |
| f_ex_ | excretion fraction | 0.96 | unity |  |
| k_L_ | permeability surface area product per unit mass for liver | k_MUS_·100 | ml·min^-1^·g^-1^ |  |
| k_S_ | permeability surface area product per unit  mass for spleen | k_L_ (due to similar capillary structure) | ml·min^-1^·g^-1^ |  |
| [R_L,0_] | binding site density liver | [R_PRO,0_]·0.05 | nmol·l^-1^ |  |
| [R_S,0_] | binding site density spleen | [R_PRO,0_]·0.02 | nmol·l^-1^ |  |
| [R_K,0_] | binding site density kidneys | fitted | nmol·l^-1^ |  |
| λ_L, int_ | internalization rate PSMA liver | λ_TU, int_ | min^-1^ | f |
| λ_S, int_ | internalization rate PSMA spleen | λ_TU, int_ | min^-1^ | f |
| λ_K, int_ | internalization rate PSMA kidneys | λ_TU, int_ | min^-1^ | f |
| λ_L,release_ | release rate liver | λ_K,release_ | min^-1^ |  |
| λ_S,release_ | release rate spleen | λ_K,release_ | min^-1^ |  |
| λ_K,release_ | release rate kidneys | fitted | min^-1^ |  |
|  |  |  |  |  |
| **Other organs** | | | | |
|  |  |  |  |  |
| V_PRO,total_ | volume total prostate | 0.016·BW/71 | l |  |
| V_LU,total_ | volume total lungs | 1·BW/71 | l |  |
| V_SAL,total_ | volume total salivary glands  = left plus right parotid gland | measured | l |  |
| V_MUS,total_ | volume total muscles | 30.078·BW/71 | l |  |
| V_GI,total_ | volume total GI + pancreas | (0.385+0.548+0.104+0.15)·BW/71 | l |  |
| V_SKIN,total_ | volume total skin | 3.408·BW/71 | l |  |
| V_ADI,total_ | volume total adipose tissue | 13.465·BW/71 | l |  |
| V_RM,total_ | volume total red marrow | 1.1·BW/71 | l |  |
| V_BONE,total_ | volume total bone without red marrow | 10.165·BW/71 - V_RM,total_ | l |  |
| V_HRT,total_ | volume total heart | 0.341·BW/71 | l |  |
| V_BR,total_ | volume total brain | 1.45·BW/71 | l |  |
| V_BW_ | volume of total body based on BW | 1 ml ≙ 1 g | l |  |
| V_REST,total_ | volume of rest body  i = all organs except tumor | $V_{BW}-\sum_{i} V_{i,total}$ | l |  |
| V_PRO,v_ | vascular volume prostate | 0.004 ·(1-H)·V_PRO,total_ | l |  |
| V_LU,v_ | vascular (serum) volume lungs | 0.105·V_P_ | l |  |
| V_SAL,v_ | vascular (serum) volume salivary glands | 0.03·(1-H)·V_SAL,total_ | l |  |
| V_MUS,v_ | vascular (serum) volume muscles | 0.14·V_P_ | l |  |
| V_GI,v_ | vascular (serum) volume GI+ pancreas | 0.076·V_P_ | l |  |
| V_SKIN,v_ | vascular(serum) volume skin | 0.03·V_P_ | l |  |
| V_ADI,v_ | vascular(serum) volume adipose tissue | 0.05·V_P_ | l |  |
| V_RM,v_ | vascular(serum) volume red marrow | 0.04·V_P_ | l |  |
| V_BONE,v_ | vascular volume bone without red marrow | 0.07·V_P_ -V_RM_ | l |  |
| V_HRT,v_ | vascular (serum) volume heart (supply) | 0.01·V_P_ | l |  |
| V_BR,v_ | vascular(serum) volume brain | 0.012·V_P_ | l |  |
| V_REST,v_ | serum volume rest i = all organs except tumor | $V_{P}-\sum_{i} V_{i,v}$ | l |  |
| V_ART_ | arterial serum plus ½ serum content of heart | 0.06·V_P_ + 0.045·V_P_ | l |  |
| V_VENES_ | venous serum plus ½ serum content of heart | 0.18·V_P_ + 0.045·V_P_ | l |  |
| V_PRO,int_ | interstitial fraction prostate | 0.25·V_PRO,total_ | l |  |
| V_LU,int_ | interstitial fraction lungs | V_LU,v_·α_LU_ | l |  |
| V_SAL,int_ | interstitial fraction salivary glands | 0.23·V_SAL,total_ | l |  |
| V_MUS,int_ | interstitial fraction muscles | V_MUS,v_·α_MUS_ | l |  |
| V_GI,int_ | interstitial fraction GI+ pancreas | V_GI,v_·α_GI_ | l |  |
| V_SKIN,int_ | interstitial fraction skin | V_SKIN,v_·α_SKIN_ | l |  |
| V_ADI,int_ | interstitial fraction adipose tissue | V_ADI,v_·α_ADI_ | l |  |
| V_RM,int_ | interstitial fraction red marrow | V_RM,v_·α_RM_ | l |  |
| V_BONE,int_ | interstitial fraction bone without red marrow | V_BONE,v_·α_BONE_ | l |  |
| V_HRT,int_ | interstitial fraction heart | V_HRT,v_·α_HRT_ | l |  |
| V_REST,int_ | volume of rest body | V_REST,v_·α_REST_ | l |  |
| α_MUS_ | ratio of interstitial to vascular volume average man | V_MUS,int_/V_MUS,v_ = 5.9 | unity |  |
| α_GI_ | ratio of interstitial to vascular volume average man | V_GI,int_/ V_GI,v_ = 8.8 | unity |  |
| α_SKIN_ | ratio of interstitial to vascular volume average man | V_SKIN,int_/ V_SKIN,v_ = 8.9 | unity |  |
| α_ADI_ | ratio of interstitial to vascular volume average man | V_ADI,int_/ V_ADI,v_ = 15. 5 | unity |  |
| α_RM_ | ratio of interstitial to vascular volume average man | V_RM,int_/ V_RM,v_ = 3.7 | unity |  |
| α_HRT_ | ratio of interstitial to vascular volume average man | V_HRT,int_/ V_HRT,v_ = 3.7 | unity |  |
| α_LU_ | ratio of interstitial to vascular volume average man | V_LU,int_/ V_LU,v_ = 5.5 | unity |  |
| α_BONE_ | ratio of interstitial to vascular volume average man | V_BONE,int_/ V_BONE,v_ = 8.4 | unity |  |
| α_REST_ | ratio of interstitial to vascular volume average man | V_REST,int_/ V_REST,v_ = 4.1 | unity |  |
|  |  |  |  |  |
| f_PRO_ | serum flow density prostate | 0.18·(1-H) | ml·min^-1^·g^-1^ |  |
| F_PRO_ | total serum flow to prostate | f_PRO_·V_PRO,total_ | ml·min^-1^ |  |
| f_SAL_ | serum flow density salivary glands | Fitted | ml·min^-1^·g^-1^ |  |
| F_SAL_ | total serum flow to salivary glands | f_SAL_·V_SAL,total_ | ml·min^-1^ |  |
| F_LU_ | total serum flow lungs | F | ml·min^-1^ |  |
| F_MUS_ | total serum flow to muscle | 0.17·F | ml·min^-1^ |  |
| F_GI_ | total serum flow to GI+ pancreas | 0.16·F | ml·min^-1^ |  |
| F_SKIN_ | total serum flow to skin | 0.05·F | ml·min^-1^ |  |
| F_ADI_ | total serum flow to adipose | 0.05·F | ml·min^-1^ |  |
| F_RM_ | total serum flow to red marrow (RM) | 0.03·F | ml·min^-1^ |  |
| F_BONE_ | total serum flow to bone (without RM) | 0.05·F | ml·min^-1^ |  |
| F_HRT_ | total serum flow to heart | 0.04·F | ml·min^-1^ |  |
| F_BR_ | total serum flow to brain | 0.12·F | ml·min^-1^ |  |
| F_REST_ | i = all organs except tumor | $F-\sum_{i} F_{i}$ | ml·min^-1^ |  |
| F_TOTAL_ |  | $F+F_{TU,1}+F_{TU,2}+F_{TU,REST}$ | ml·min^-1^ |  |
| PS_i_ | permeability surface area product | k_i_·V_i, total_ | ml·min^-1^ |  |
| k_PRO_ | permeability surface area product per unit mass (scaled for molecule size of PSMA I&T) for prostate | 0.1 | ml·min^-1^·g^-1^ |  |
| k_LU_ | permeability surface area product per unit mass for lungs | k_MUS_·100 | ml·min^-1^·g^-1^ |  |
| k_SAL_ | permeability surface area product per unit mass for salivary glands | k_MUS_·100 | ml·min^-1^·g^-1^ | for submand. glands |
| k_MUS_ | permeability surface area product per unit mass for muscle | 0.02 | ml·min^-1^·g^-1^ |  |
| k_GI_ | permeability surface area product per unit mass for GI and pancreas | 0.02  (assumed to similar to muscle) | ml·min^-1^·g^-1^ |  |
| k_SKIN_ | permeability surface area product per unit mass for skin | 0.02  (assumed to similar to muscle) | ml·min^-1^·g^-1^ |  |
| k_ADI_ | permeability surface area product per unit mass for adipose | 0.02  (assumed to similar to muscle) | ml·min^-1^·g^-1^ |  |
| k_RM_ | permeability surface area product per unit mass for red marrow | k_L_(assumed to similar to liver) | ml·min^-1^·g^-1^ |  |
| k_HRT_ | permeability surface area product per unit mass for heart | 0.02  (assumed to similar to muscle) | ml·min^-1^·g^-1^ |  |
| k_BONE_ | permeability surface area product per unit mass for bone | 0.02  (assumed to similar to muscle) | ml·min^-1^·g^-1^ |  |
| k_REST_ | permeability surface area product per unit mass for rest | 0.02  (assumed to similar to muscle) | ml·min^-1^·g^-1^ |  |
| [R_PRO,0_] | binding site density prostate | [R_TU,Rest,0_] ·0.1 | nmol l^-1^ |  |
| [R_SAL,0_] | binding site density salivary glands | fitted | nmol l^-1^ |  |
| [R_GI,0_] | binding site density GI + pancreas | [R_PRO,0_]·0.06 | nmol l^-1^ |  |
| λ_NT,int_ | internalization rate for normal tissue | λ_TU,int_ | min^-1^ | f |
| λ_NT,release_ | degradation and release normal tissue (except salivary glands) | λ_K,release_ | min^-1^ |  |
| λ_SAL,release_ | degradation and release salivary glands | fitted | min^-1^ |  |
| R | binding sites free |  | nmol |  |
| R_i_,_0_ | binding sites total number of organ i | [R_i_,_0_]·V_i,total_ | nmol |  |
| [R_i_,_0_] | binding site density of organ i |  | nmol l^-1^ |  |
| RP_i_ | peptide bound |  | nmol |  |
| PRP | peptide bound to serum protein |  | nmol |  |
| k_PR_ | binding rate peptide to serum | 4.7·10^-4^ | min^-1^ |  |
| P_intern_ | peptide internalized |  | nmol |  |
| P_i,v_ | peptide free vascular |  | nmol |  |
| P_i,int_ | peptide free interstitial |  | nmol |  |
| P_K,intra_ | peptide interacellular kidneys |  | nmol |  |
| P_inj_ | injected amount of unlabeled peptide | P1-5: 139; 91; 81; 67;294 | nmol |  |
| P*_inj_ | injected amount of labeled peptide | P1-5: 8.4;7.5; 7.5;7.5;7.8 | nmol |  |

^a^Mean values from all measured (Biacore) ligands.

^b^For the average normal adult (blood) F = 6500 ml/min and V = 5300 ml. Therefore, a factor of 1.23 was assigned to account for the changes in total serum flow due to volume changes.

^c^Using the assumption of 266 nmol·l^-1^ binding site density , 10^12^ cells per liter and 10 ml or 50 ml addition tumor volume.

^d^It is assumed that 2/3 of the total intracellular volume of the kidneys is represented by the proximal tubular cells

^e^Scaling of GFR due to different molecular sizes

^f^Antunes et al. do not report values for ^177^Lu. However, for the investigated ligands labeled with ^111^In, kidney and tumor cells showed similar internalization in many cases.

**Absorbed dose (D) and biologically effective dose (BED):**

To calculate the absorbed dose (only self-dose was considered, except for the red marrow) and the BED of the kidneys, tumor and salivary glands, the following equations and parameter values (Table B) were used:

$\mathop{D}\limits^{\bullet} _{i}(t)=A_{i}(t)\cdot S_{i\leftarrow i}=A_{inj}\cdot a_{i}(t)\cdot S_{i\leftarrow i}$ (14)

$D_{i}(T)_{\text{ }}=\int_{0}^{T} \mathop{D_{i}}\limits^{\bullet} (t)dt=A_{inj}\cdot\tilde{a}_{i}(T)\cdot S_{i\leftarrow i}$ (15)

The BED is defined as

(16)

The factor G_i_ (Lea–Catcheside factor) is defined as

$G_{i}(T)_{\text{ }}=\frac{2}{D_{i}^{2}}\cdot\int_{0}^{T} \mathop{D_{i}}\limits^{\bullet} (t)dt\cdot\int_{0}^{t} \mathop{D_{i}}\limits^{\bullet} (\omega)\cdot e^{-\mu_{i}(t-\omega)}d\omega$ (17)

Thus, after inserting Eq. (17) in (16) one obtains

(18)

The activity to administer yielding a fixed kidney BED_K, fixed_ is calculated

according to:

(19)

Solving Eq (19) for the administered activity yields

The activity to administer yielding a fixed salivary glands absorbed dose is calculated using equation (15):

(21)
To calculate the BED of the red marrow, equation (22) was used instead of Eq. (14):

(22)

**Table B**

| **Variable** |  | **Value** | **Unit** | **Source** |
| --- | --- | --- | --- | --- |
| S_K🡨K_ | Dose factor kidneys to kidneys phantom | 4.82·10^-6^ | Gy·min^-1^·MBq^-1^ |  |
| S_SAL🡨SAL_ | Dose factor kidneys to kidneys phantom | Table C | Gy·min^-1^·MBq^-1^ |  |
| S_TU🡨TU_ | Dose factor tumor to tumor phantom | Table C | Gy·min^-1^·MBq^-1^ |  |
| S_RM🡨RM_ | Dose factor red marrow to red marrow phantom (are scaled using BW) | 7.14·10^-7^ | Gy·min^-1^·MBq^-1^ |  |
| S_RM🡨REM_ | Dose factor remainder to red marrow  corrected according to Hindorf et al. | 4.83·10^-9^ | Gy·min^-1^·MBq^-1^ |  |
| *α/β_K_* | radiobiological parameters kidneys | 2.5 | Gy |  |
| *µ_K_* | repair rate kidneys | ln(2)/60/2.8 | min^-1^ |  |
| *α_SAL_/ β_SAL_* | radiobiological parameters salivary glands | 0.053/0.0118 | Gy |  |
| *µ_SAL_* | repair rate liver | 0.0077 | min^-1^ |  |
| *α/β_TU_* | radiobiological parameters tumor | 3.9 | Gy |  |
| *µ_TU_* | repair rate tumor | ln(2)/60/0.5 | min^-1^ |  |
| *α/β_RM_* | radiobiological parameters red marrow | 15 | Gy |  |
| *µ_RM_* | repair rate red marrow | ln(2)/60/1.5 | min^-1^ |  |
| *A_i_* | activity of organ i |  | MBq |  |
| *A_REM_* | activity of remainder (tumor is part of the remainder) | A_Total_ -A_SAL_-A_K_-A_RM_ | MBq |  |
| *A_inj_* | injected activity |  | MBq |  |
| *A_inj, BED K,fixed_* | activity to inject for BED_K_ = 10 Gy |  | MBq |  |
| *ã_i_* | time-integrated activity coefficient of organ i |  | h |  |
| *a_i_* | fraction of administered activity of organ i |  | unity |  |
| *D_i_* | dose to organ i |  | Gy |  |
| *Ḋ_i_* | dose rate to organ i |  | Gy·min^-1^ |  |
| *T* | Integration time | 30000 | min |  |
| *G_i_* | Lea–Catcheside factor of organ i |  | unity |  |
| *BED_i_* | biologically effective dose to organ i |  | Gy |  |

**Table C S-Values for tumor and salivary glands**

| **Volume [ml]** | **S-values [Gy∙min^-1^∙MBq^-1^]** |
| --- | --- |
| 0.5 | 2.77·10^-3^ |
| 1 | 1.40·10^-3^ |
| 1.5 | 9.00·10^-4^ |
| 2 | 7.02·10^-4^ |
| 3 | 4.50·10^-4^ |
| 4 | 3.52·10^-4^ |
| 13 | 1.10·10^-4^ |
| 17 | 8.30·10^-5^ |
| 21 | 6.90·10^-5^ |
| 29 | 4.80·10^-5^ |
| 34 | 4.30·10^-5^ |
| 52 | 2.67·10^-5^ |
| 54 | 2.65·10^-5^ |

**References**

1. Rippe B, Haraldsson B. Fluid and protein fluxes across small and large pores in the microvasculature. Application of two-pore equations. acta Physiol Scand. 1987;131(3):411-28.

2. Winter G, Drescher A, Baur B, Solbach C, Reske SN, Beer AJ. Comparative analysis of chelator-modified peptides for imaging of prostate carcinoma. Annual Congress EANM. 2014(P200).

3. Weineisen M, Schottelius M, Simecek J, Baum RP, Yildiz A, Beykan S, et al. 68Ga- and 177Lu-Labeled PSMA I&T: Optimization of a PSMA-Targeted Theranostic Concept and First Proof-of-Concept Human Studies. J Nucl Med. 2015;56(8):1169-76.

4. Leggett RW, Williams LR. A proposed blood circulation model for reference man. Health Phys. 1995;69(2):187-201.

5. Buchmann I, Kull T, Glatting G, Bunjes D, Hale G, Kotzerke J, et al. A comparison of the biodistribution and biokinetics of 99mTc-anti-CD66 mAb BW 250/183 and 99mTc-anti-CD45 mAb YTH 24.5 with regard to suitability for myeloablative radioimmunotherapy. Eur J Nucl Med Mol Imaging. 2003;30(5):667-73.

6. Buckley DL, Roberts C, Parker GJ, Logue JP, Hutchinson CE. Prostate cancer: evaluation of vascular characteristics with dynamic contrast-enhanced T1-weighted MR imaging--initial experience. Radiology. 2004;233(3):709-15.

7. Luczynska E, Heinze-Paluchowska S, Blecharz P, Jereczek-Fossa B, Petralia G, Bellomi M, et al. Correlation between CT perfusion and clinico-pathological features in prostate cancer: a prospective study. Med Sci Monit. 2015;21:153-62.

8. Franiel T, Lüdemann L, Rudolph B, Rehbein H, Stephan C, Taupitz M, et al. Prostate MR imaging: tissue characterization with pharmacokinetic volume and blood flow parameters and correlation with histologic parameters. Radiology. 2009;252(1):101-8.

9. Wang X, Ma D, Olson WC, Heston WD. In vitro and in vivo responses of advanced prostate tumors to PSMA ADC, an auristatin-conjugated antibody to prostate-specific membrane antigen. Mol Cancer Ther. 2011;10(9):1728-39.

10. Kletting P, Kull T, Maass C, Malik N, Luster M, Beer A, et al. Optimized peptide amount and activity for Y-90-labeled DOTATATE therapy. J Nucl Med. 2015.

11. Stabin MG, Sparks RB, Crowe E. OLINDA/EXM: The Second-Generation Personal Computer Software for Internal Dose Assessment in Nuclear Medicine. J Nucl Med. 2005;46(6):1023-7.

12. Shah DK, Betts AM. Towards a platform PBPK model to characterize the plasma and tissue disposition of monoclonal antibodies in preclinical species and human. J Pharmacokinet Pharmacodyn. 2012;39(1):67-86.

13. Schmidt MM, Wittrup KD. A modeling analysis of the effects of molecular size and binding affinity on tumor targeting. Mol Cancer Ther. 2009;8(10):2861-71.

14. Fresco GF, DiGiorgio F, Curti GL. Simultaneous estimation of glomerular filtration rate and renal plasma flow. J Nucl Med. 1995;36(9):1701-6.

15. Kletting P, Muller B, Erentok B, Schmaljohann J, Behrendt FF, Reske SN, et al. Differences in predicted and actually absorbed doses in peptide receptor radionuclide therapy. Med Phys. 2012;39(9):5708-17.

16. Groothuis DR. The blood-brain and blood-tumor barriers: a review of strategies for increasing drug delivery. Neuro-oncology. 2000;2(1):45-59.

17. O'Keefe DS, Bacich DJ, Heston WD. Comparative analysis of prostate-specific membrane antigen (PSMA) versus a prostate-specific membrane antigen-like gene. Prostate. 2004;58(2):200-10.

18. Antunes P, Ginj M, Zhang H, Waser B, Baum RP, Reubi JC, et al. Are radiogallium-labelled DOTA-conjugated somatostatin analogues superior to those labelled with other radiometals? Eur J Nucl Med Mol Imaging. 2007;34(7):982-93.

19. Velikyan I, Sundin A, Eriksson B, Lundqvist H, Sorensen J, Bergstrom M, et al. In vivo binding of [68Ga]-DOTATOC to somatostatin receptors in neuroendocrine tumours--impact of peptide mass. Nucl Med Biol. 2010;37(3):265-75.

20. Snyder WS, Cook MJ, Nasset ES, Karhausen RS, Howells GP. Report of the Task Group on Reference Man. ICRP publication 23. Oxford: Elsevier; 1975.

21. Berggreen E, Wiig H. Lowering of interstitial fluid pressure in rat submandibular gland: a novel mechanism in saliva secretion. American journal of physiology Heart and circulatory physiology. 2006;290(4):H1460-8.

22. Clough G, Smaje LH. Exchange area and surface properties of the microvasculature of the rabbit submandibular gland following duct ligation. The Journal of physiology. 1984;354:445-56.

23. Ben Jemaa A, Bouraoui Y, Sallami S, Banasr A, Ben Rais N, Ouertani L, et al. Co-expression and impact of prostate specific membrane antigen and prostate specific antigen in prostatic pathologies. J Exp Clin Cancer Res. 2010;29:171.

24. Hobbs RF, Sgouros G. Calculation of the biological effective dose for piecewise defined dose-rate fits. Med Phys. 2009;36(3):904-7.

25. Stabin MG, Siegel JA. Physical models and dose factors for use in internal dose assessment. Health Phys. 2003;85(3):294-310.

26. Hindorf C, Glatting G, Chiesa C, Lindén O, Flux G. EANM Dosimetry Committee guidelines for bone marrow and whole-body dosimetry. Eur J Nucl Med Mol Imaging. 2010;37(6):1238-50.

27. Cremonesi M, Botta F, Di Dia A, Ferrari M, Bodei L, De Cicco C, et al. Dosimetry for treatment with radiolabelled somatostatin analogues. A review. Q J Nucl Med Mol Imaging. 2010;54(1):37-51.

28. Hobbs RF, Jentzen W, Bockisch A, Sgouros G. Monte Carlo-based 3-dimensional dosimetry of salivary glands in radioiodine treatment of differentiated thyroid cancer estimated using 124I PET. Q J Nucl Med Mol Imaging. 2013;57(1):79-91.

29. Kal HB, Van Gellekom MP. How low is the alpha/beta ratio for prostate cancer? Int J Radiat Oncol Biol Phys. 2003;57(4):1116-21.

30. Dale RG. The application of the linear-quadratic dose-effect equation to fractionated and protracted radiotherapy. Br J Radiol. 1985;58(690):515-28.

31. Konijnenberg M. From imaging to dosimetry and biological effects. Q J Nucl Med Mol Imaging. 2011;55(1):44-56.

**PBPK Model compartments**


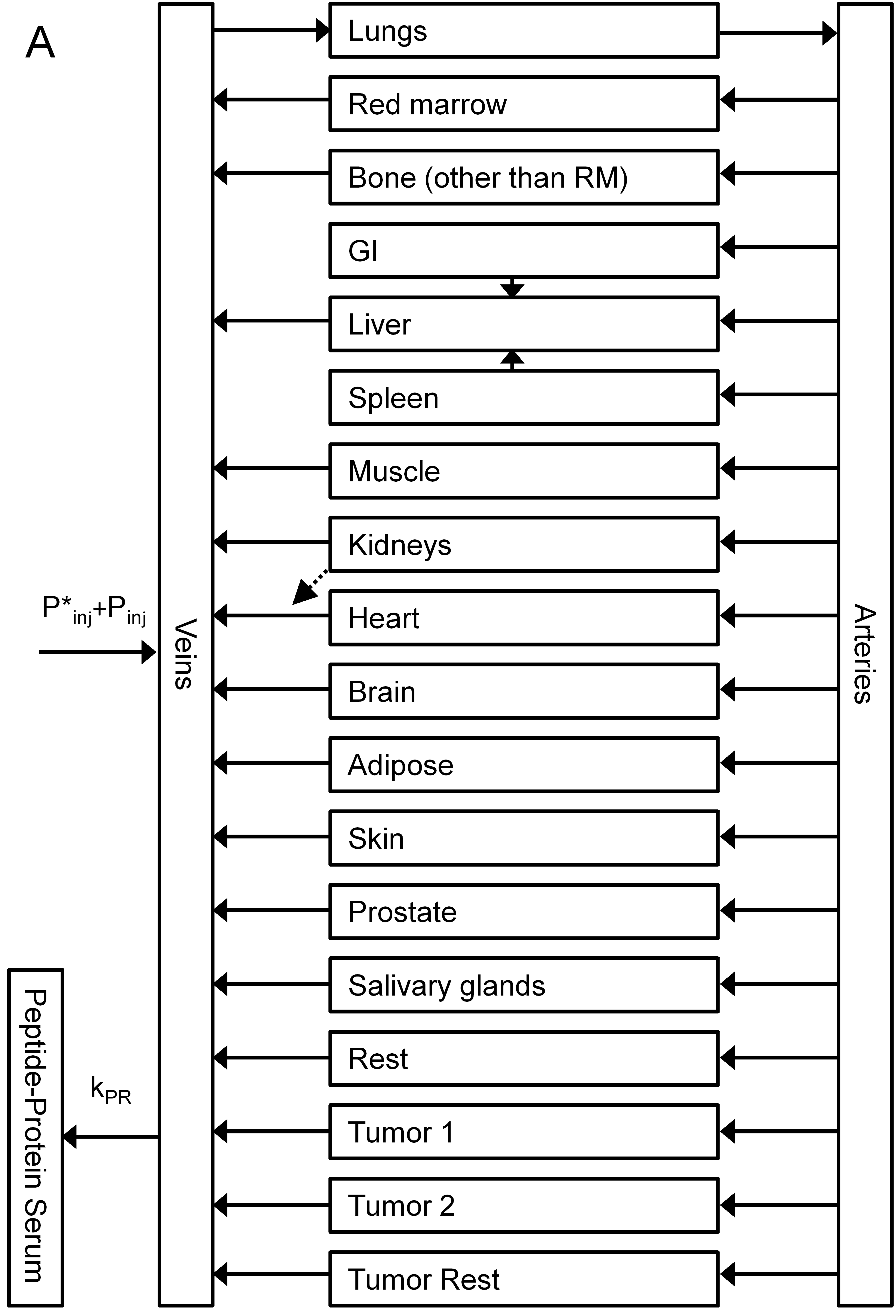


**A Main model structure**: All organs are represented by a rectangular compartment and connected via the serum flow. Each organ within this model, except arteries, veins, brain and protein serum, is divided into sub-compartments. The substance is cleared via the kidney. The compartment “Peptide-Protein serum” contains peptide bound to serum protein. As the fraction of bound peptide to proteins is small compared to the total amount and to reduce complexity, only the „veins“ were connected to this compartment. The corresponding fraction for each specific organ is considered in the fitting process by assigning the data to the specific compartments.


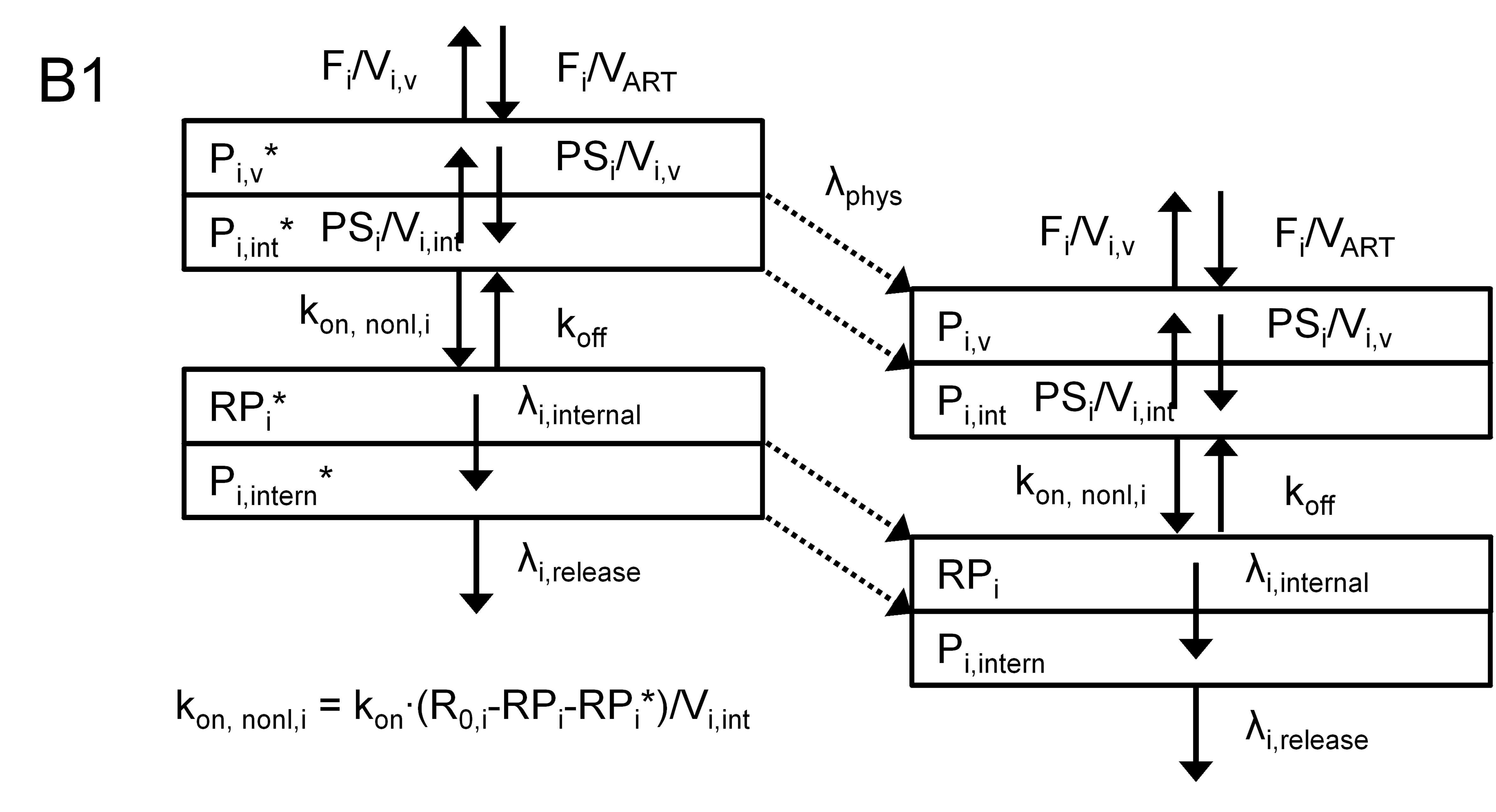


**B1 GI, spleen, prostate, salivary glands and tumor :**The entire model consists of three systems, one for labeled (with *) and one for unlabeled peptide. The systems are connected by the competition for free binding sites (k_on,nonl,i_ = k_on_∙(R_0,i_-RP_i_-RP_i_*)/V_i,int_ ) and by physical decay (λ_phys_). All physiological parameters are assumed to be equal for the labeled and unlabeled substance.

k_off_ is the dissociation rate, the transport of peptide via serum flow to organ i is described by F_i_/V_ART_ (where F_i_ is serum flow and V_ART_ is serum volume of the arteries), F_i_ /V_i,v_ describes the transport of peptide via serum flow out of organ and (where F_i_ is serum flow and V_i,v_ is serum volume of the respective organ, RP_i_ is PSMA specific bound peptide to the cell surface, P_i,v_ and P_i,int_ is free peptide of the vascular (V_i,v_) and interstitial space (V_i,int_) , respectively. PS_i_ is the permeability surface area product and λ _i, internal_ is the internalisation rate of bound peptide and λ _i, release_ the release rate of ^177^Lu from the cell.


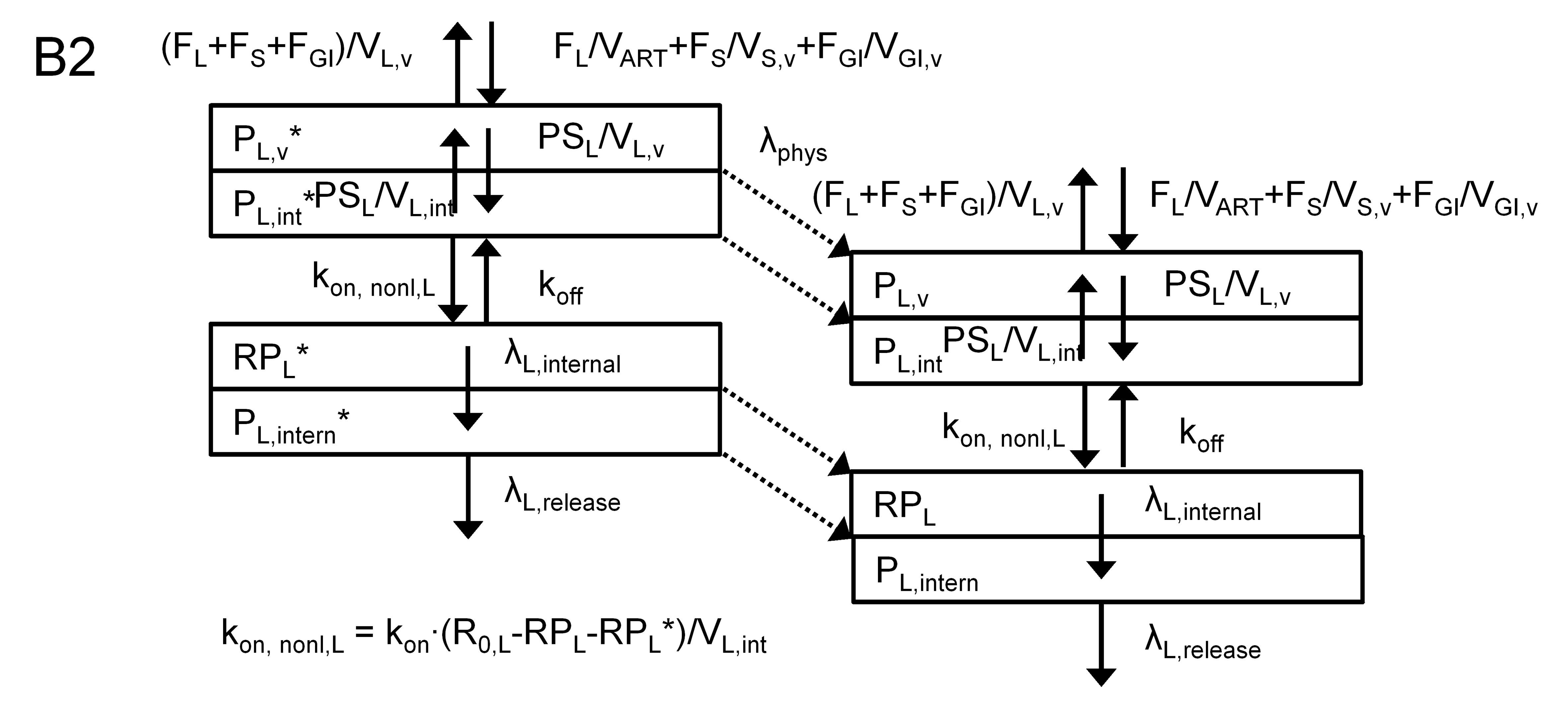


**B2 Liver:** For the liver the model description of B1 applies but the serum flow is composed of liver arterial, GI and spleen flow.


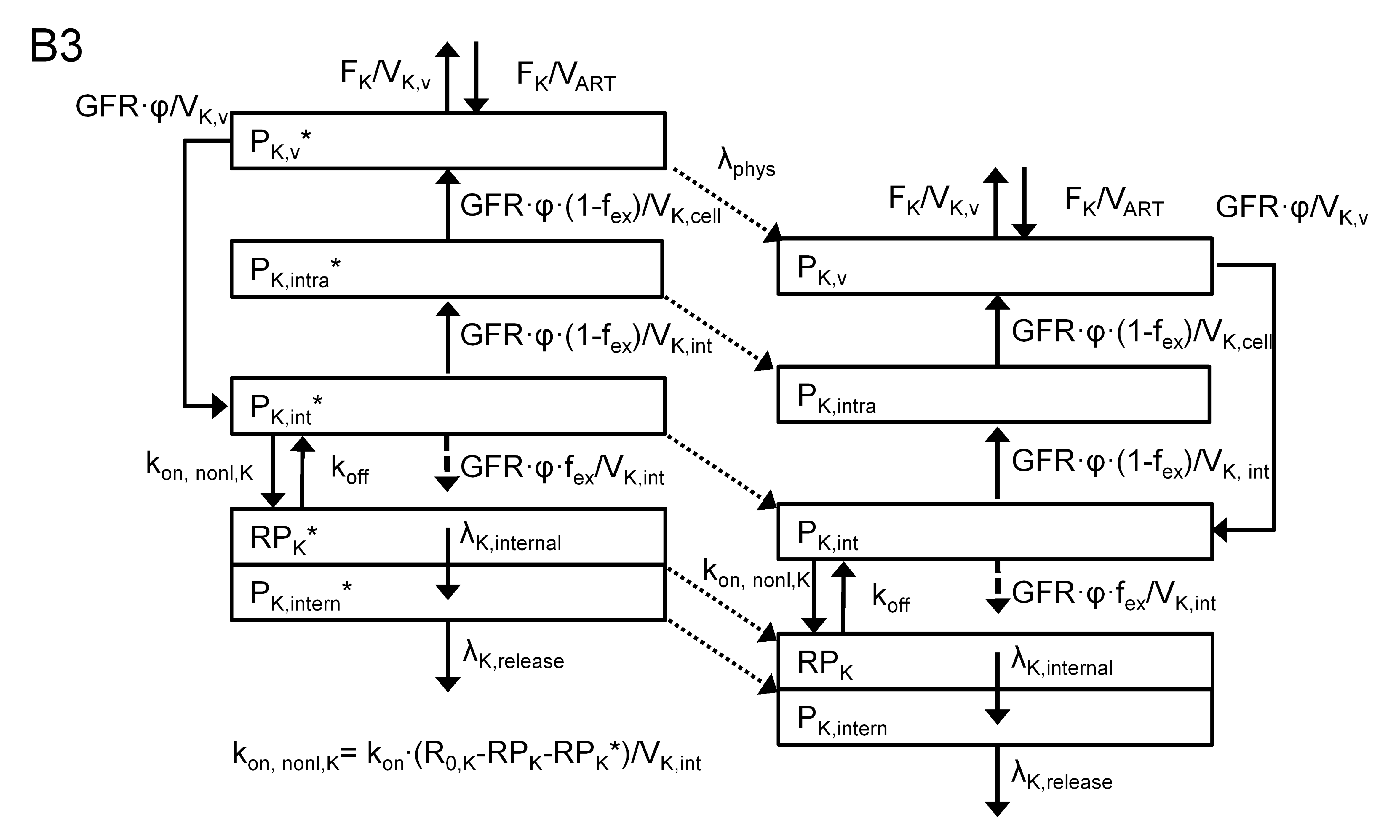


**B3 Kidneys:** The peptide is transported via serum flow to the vascular compartment then filtrated into the interstitial part. Due to the administration of amino acids the largest fraction (f_ex_ = 0.96) of peptide is excreted. All unspecific uptake mechanisms are modelled with flow GFR·φ·(1-f_ex_) in and out of kidney cells. GFR was measured with Cr-51-EDTA.


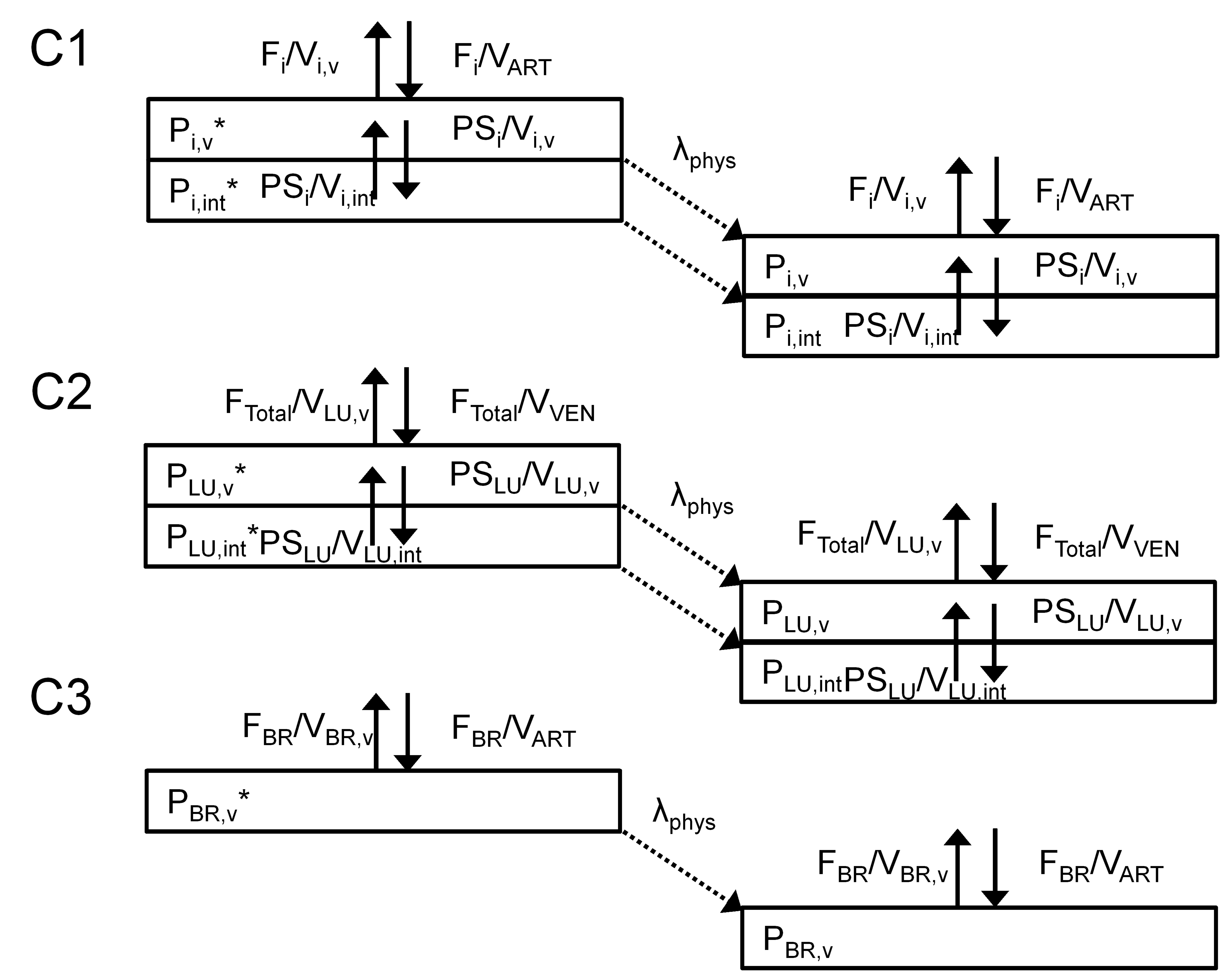


**C PSMA negative tissue and brain:** For adipose, bone (other than red marrow), skin, heart (C1) and lung (C2) the model on the organ level simplifies to the transport of peptide via serum flow and transcapillary extravasation. For brain (C3) the model reduces to serum flow.


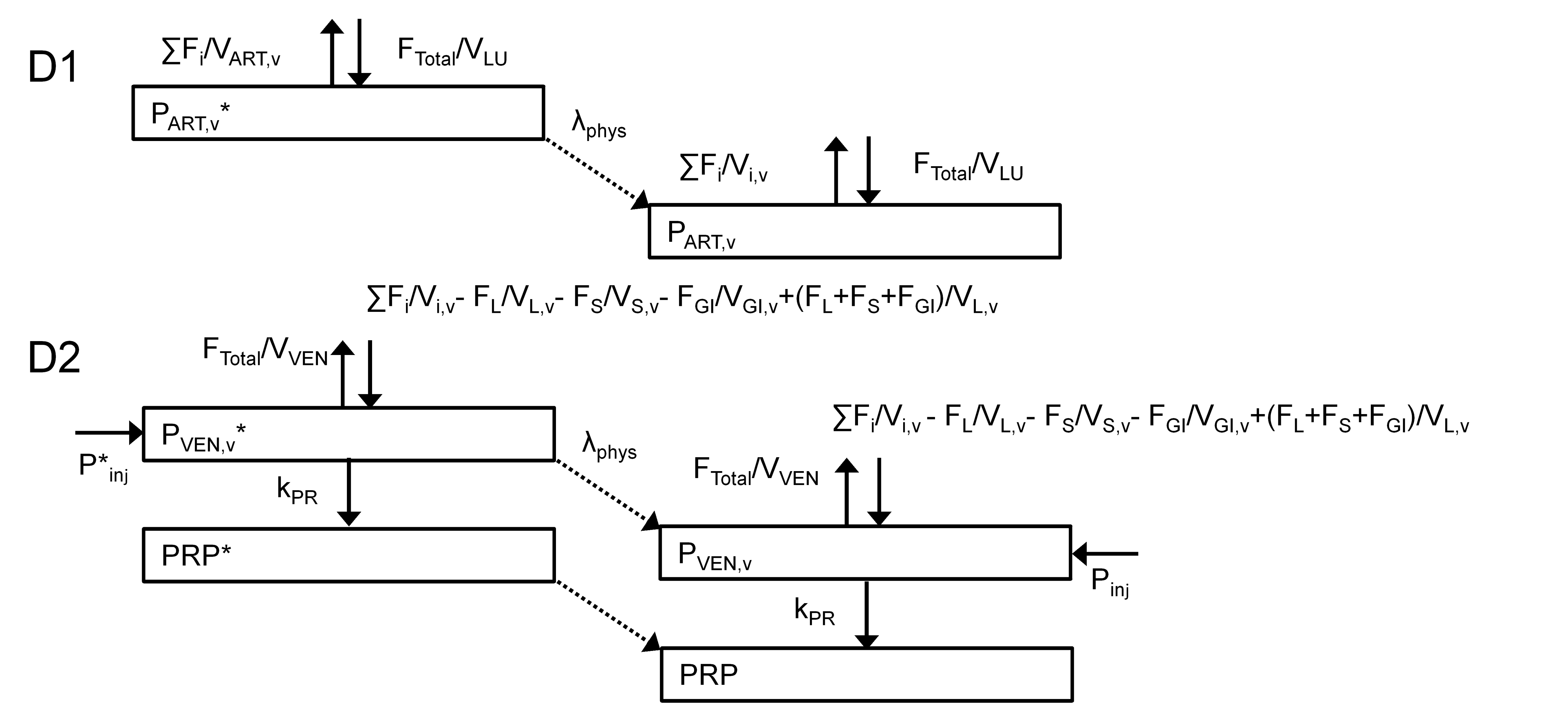


**D Arteries and veins**: As the fraction of bound peptide to proteins (PRP) is small compared to the total amount and to reduce complexity, only the „veins (D2)“ were connected to PRP. The corresponding fraction for each specific organ is considered in the fitting process by assigning the data to the specific compartments.

**Assigning data to compartments**

Salivary glands

data_SAL_=P^*^_SAL_,_v_+P^*^_SAL_,_int_+RP^*^_SAL_+P^*^_SAL_,_intern_+PRP^*^·V_SAL,v_/(V_P_+V_TU,1,v_+V_TU,2,v_+V_TU,Rest,v_)

Tumor

Data_TU,1_=P^*^_TU,1,v_+P^*^_TU,1,int_+RP^*^_TU,1_+P^*^_TU,1,intern_+PRP^*^·V_TU,1,v_/(V_P_+V_TU,1,v_+V_TU,2,v_+V_TU,Rest,v_)

+x_tu,1_· (P^*^_MUS,v_+P^*^_MUS,int_+PRP^*^·V_MUS,v_/(V_P_+V_TU,1,v_+V_TU,2,v_+V_TU,Rest,v_))

Data_TU,2_=P^*^_TU,2,v_+P^*^_TU,2,int_+RP^*^_TU,2_+P^*^_TU,2,intern_+PRP^*^·V_TU,2,v_/(V_P_+V_TU,1,v_+V_TU,2,v_+V_TU,Rest,v_)

+x_tu,2_·(P^*^_MUS,v_+P^*^_MUS,int_+PRP^*^·V_MUS,v_/(V_P_+V_TU,1,v_+V_TU,2,v_+V_TU,Rest,v_))

Where x_tu,1_ and x_tu,2_ are fitted.

Kidneys

Data_K_=P^*^_K_,_v_+P^*^_K_,_int_+RP^*^_K_+P^*^_K_,_intern_+P^*^_K_,_intra_+PRP^*^·V_K,v_/(V_P_+V_TU,1,v_+V_TU,2,v_+V_TU,Rest,v_)

Total body

All hot (^*^) compartments
