# Supplementary material for: Investigating the Effect of Ligand Amount and Injected Therapeutic Activity: A Simulation Study for 177Lu-Labeled PSMA-Targeting Peptides
Source: PLoS One. 2016 Sep 9;11(9):e0162303. doi: 10.1371/journal.pone.0162303 (PMC5017739; doi:10.1371/journal.pone.0162303)
Supplement: S2 File — Estimated parameters, fitted curves as well as the pertaining BED simulations for all patients. (PPTX) [file pone.0162303.s002.pptx]

## Slide 1
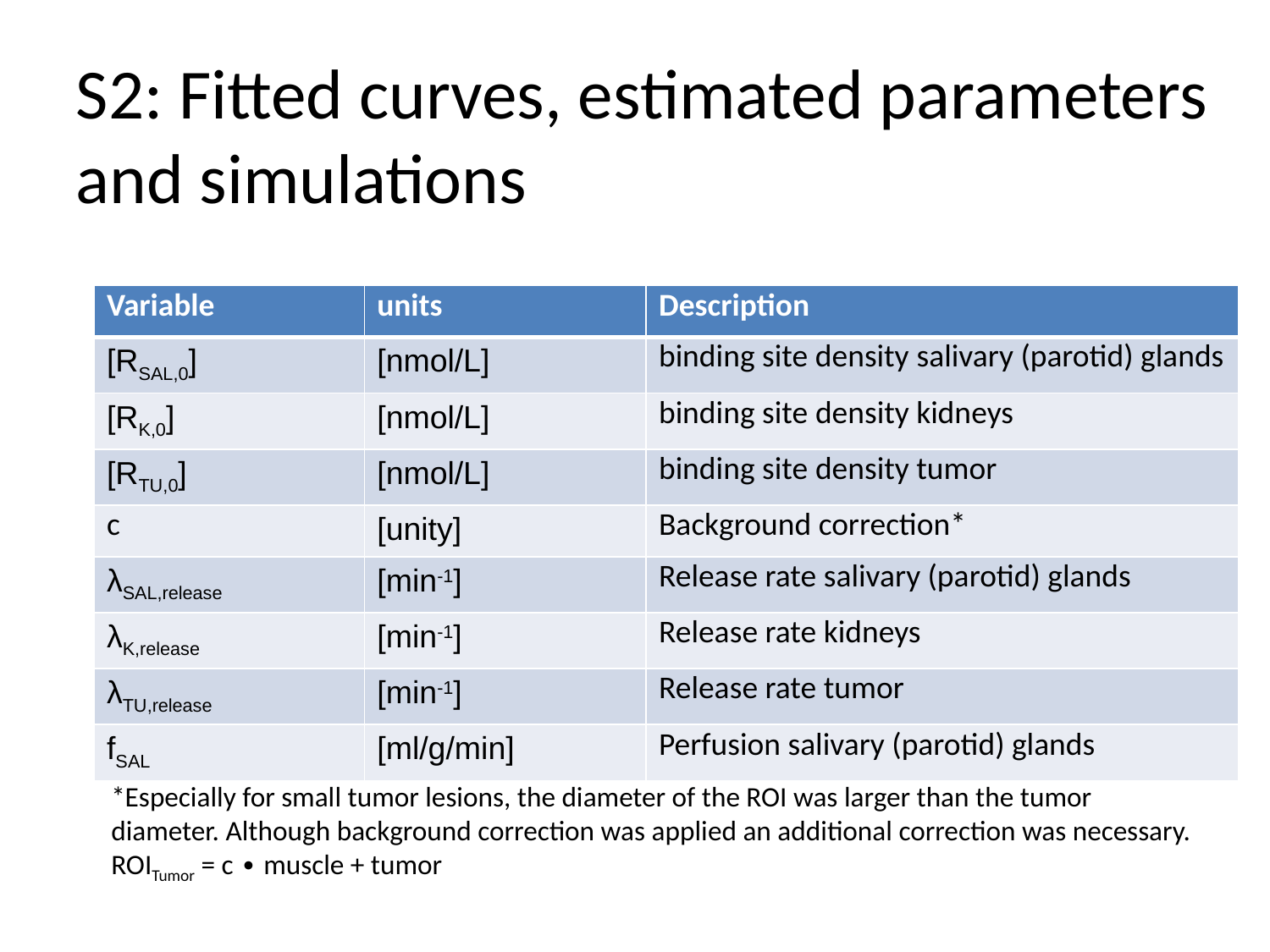

S2: Fitted curves, estimated parameters and simulations
| Variable | units | Description |
| --- | --- | --- |
| [RSAL,0] | [nmol/L] | binding site density salivary (parotid) glands |
| [RK,0] | [nmol/L] | binding site density kidneys |
| [RTU,0] | [nmol/L] | binding site density tumor |
| c | [unity] | Background correction\* |
| λSAL,release | [min-1] | Release rate salivary (parotid) glands |
| λK,release | [min-1] | Release rate kidneys |
| λTU,release | [min-1] | Release rate tumor |
| fSAL | [ml/g/min] | Perfusion salivary (parotid) glands |
*Especially for small tumor lesions, the diameter of the ROI was larger than the tumor
diameter. Although background correction was applied an additional correction was necessary.
ROITumor = c ∙ muscle + tumor

## Slide 2
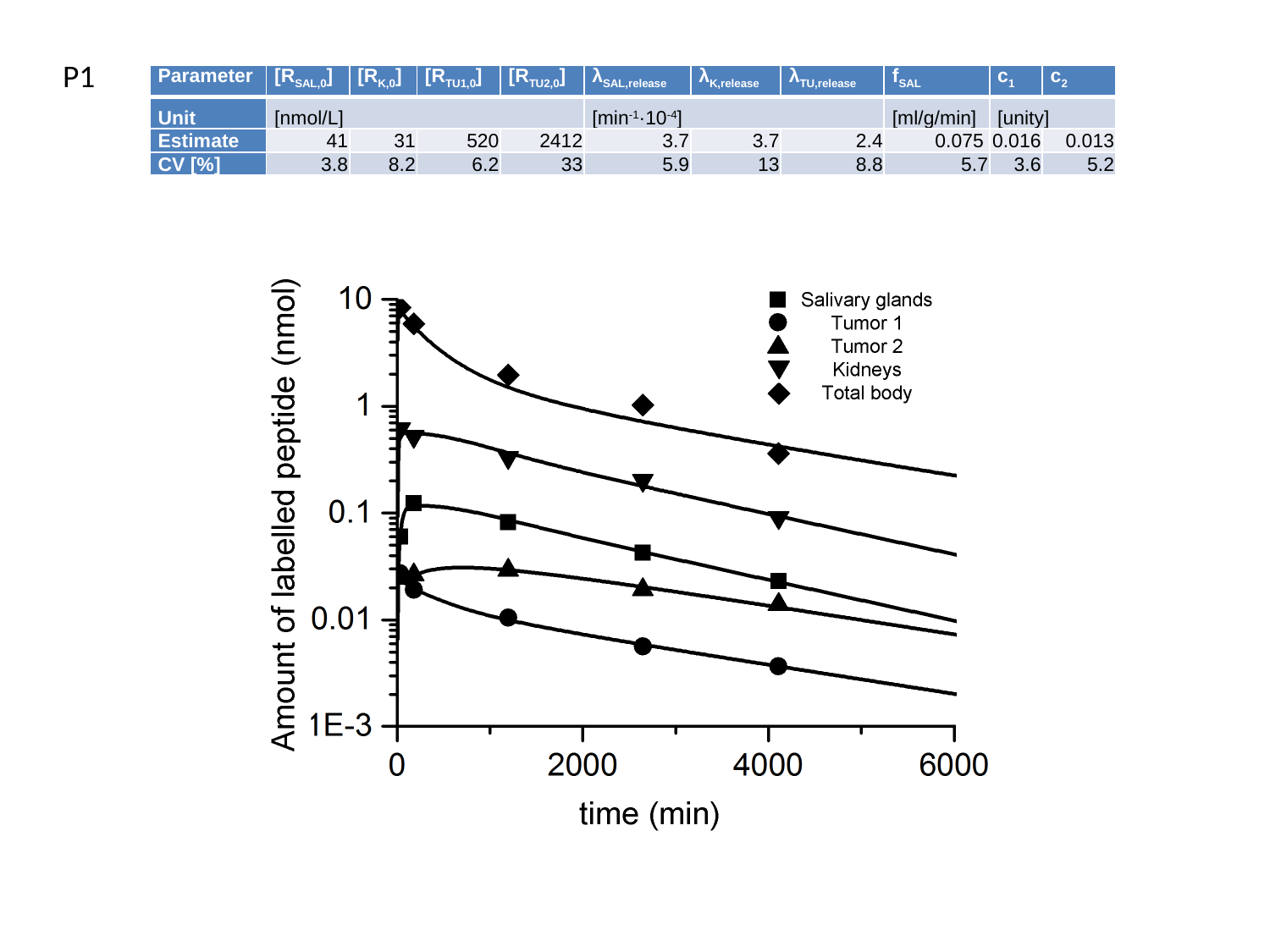

P1
| Parameter | [RSAL,0] | [RK,0] | [RTU1,0] | [RTU2,0] | λSAL,release | λK,release | λTU,release | fSAL | c1 | c2 |
| --- | --- | --- | --- | --- | --- | --- | --- | --- | --- | --- |
| Unit | [nmol/L] | | | | [min-1·10-4] | | | [ml/g/min] | [unity] | |
| Estimate | 41 | 31 | 520 | 2412 | 3.7 | 3.7 | 2.4 | 0.075 | 0.016 | 0.013 |
| CV [%] | 3.8 | 8.2 | 6.2 | 33 | 5.9 | 13 | 8.8 | 5.7 | 3.6 | 5.2 |

## Slide 3
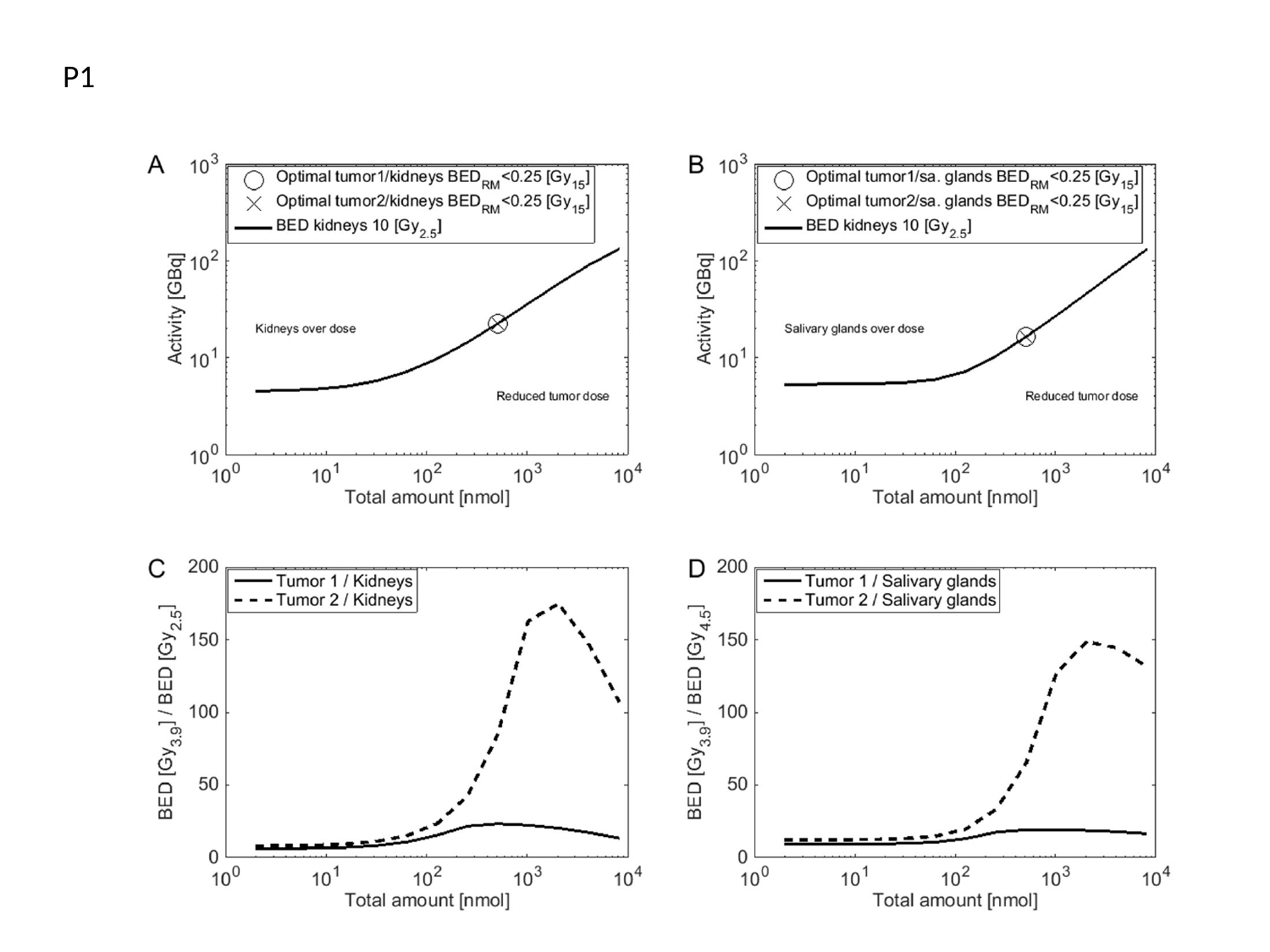

P1

## Slide 4
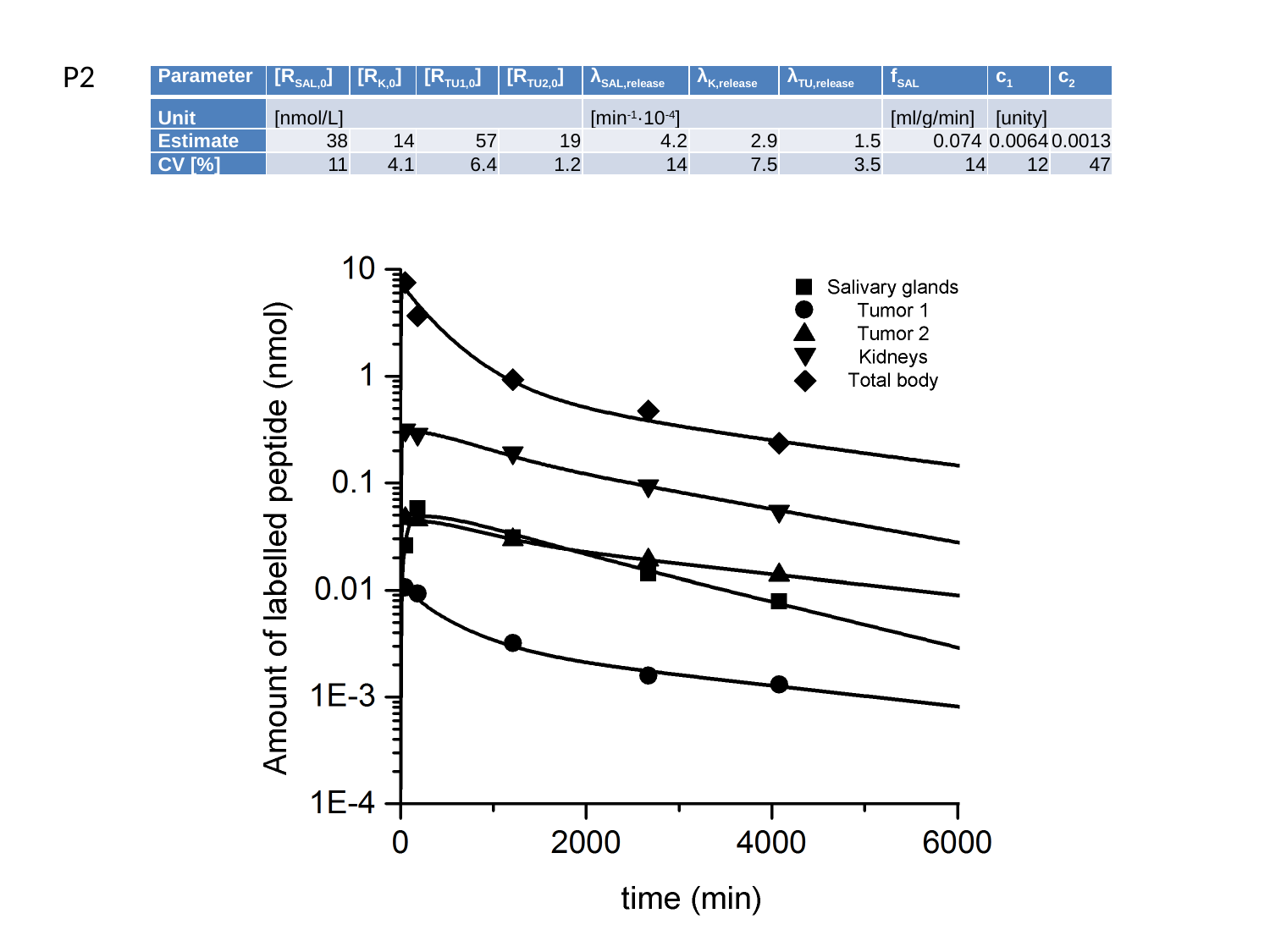

P2
| Parameter | [RSAL,0] | [RK,0] | [RTU1,0] | [RTU2,0] | λSAL,release | λK,release | λTU,release | fSAL | c1 | c2 |
| --- | --- | --- | --- | --- | --- | --- | --- | --- | --- | --- |
| Unit | [nmol/L] | | | | [min-1·10-4] | | | [ml/g/min] | [unity] | |
| Estimate | 38 | 14 | 57 | 19 | 4.2 | 2.9 | 1.5 | 0.074 | 0.0064 | 0.0013 |
| CV [%] | 11 | 4.1 | 6.4 | 1.2 | 14 | 7.5 | 3.5 | 14 | 12 | 47 |

## Slide 5
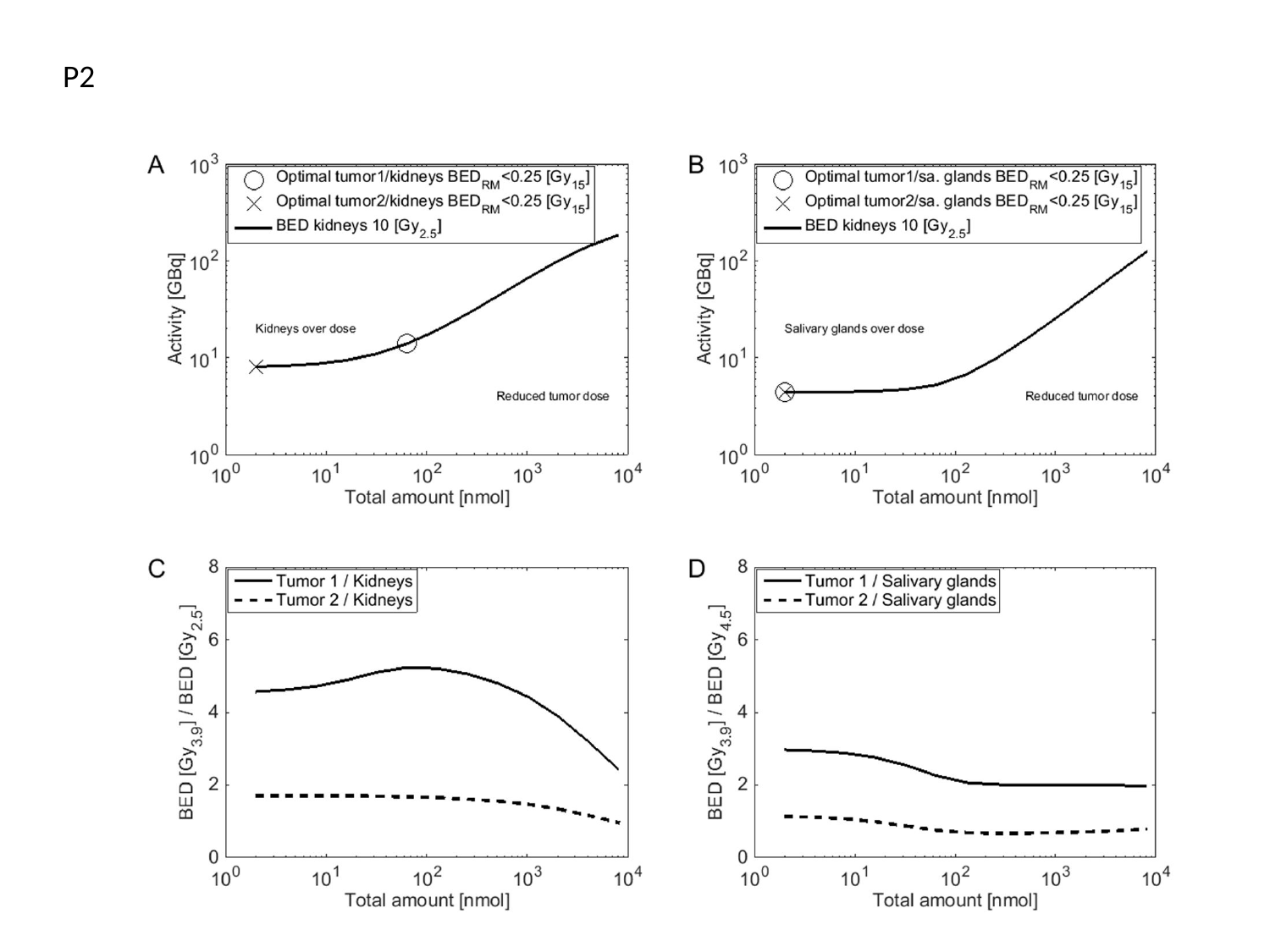

P2

## Slide 6
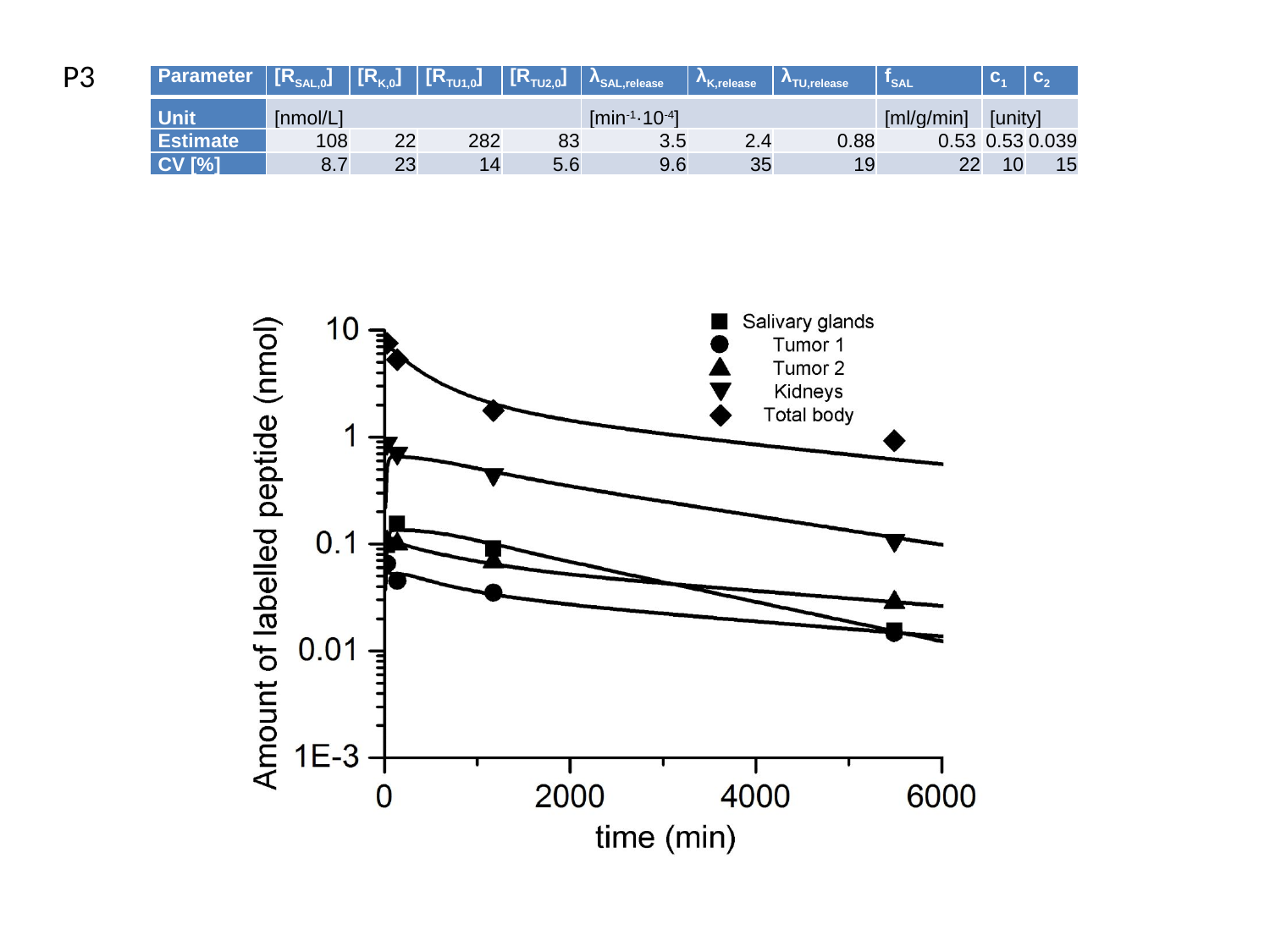

P3
| Parameter | [RSAL,0] | [RK,0] | [RTU1,0] | [RTU2,0] | λSAL,release | λK,release | λTU,release | fSAL | c1 | c2 |
| --- | --- | --- | --- | --- | --- | --- | --- | --- | --- | --- |
| Unit | [nmol/L] | | | | [min-1·10-4] | | | [ml/g/min] | [unity] | |
| Estimate | 108 | 22 | 282 | 83 | 3.5 | 2.4 | 0.88 | 0.53 | 0.53 | 0.039 |
| CV [%] | 8.7 | 23 | 14 | 5.6 | 9.6 | 35 | 19 | 22 | 10 | 15 |

## Slide 7
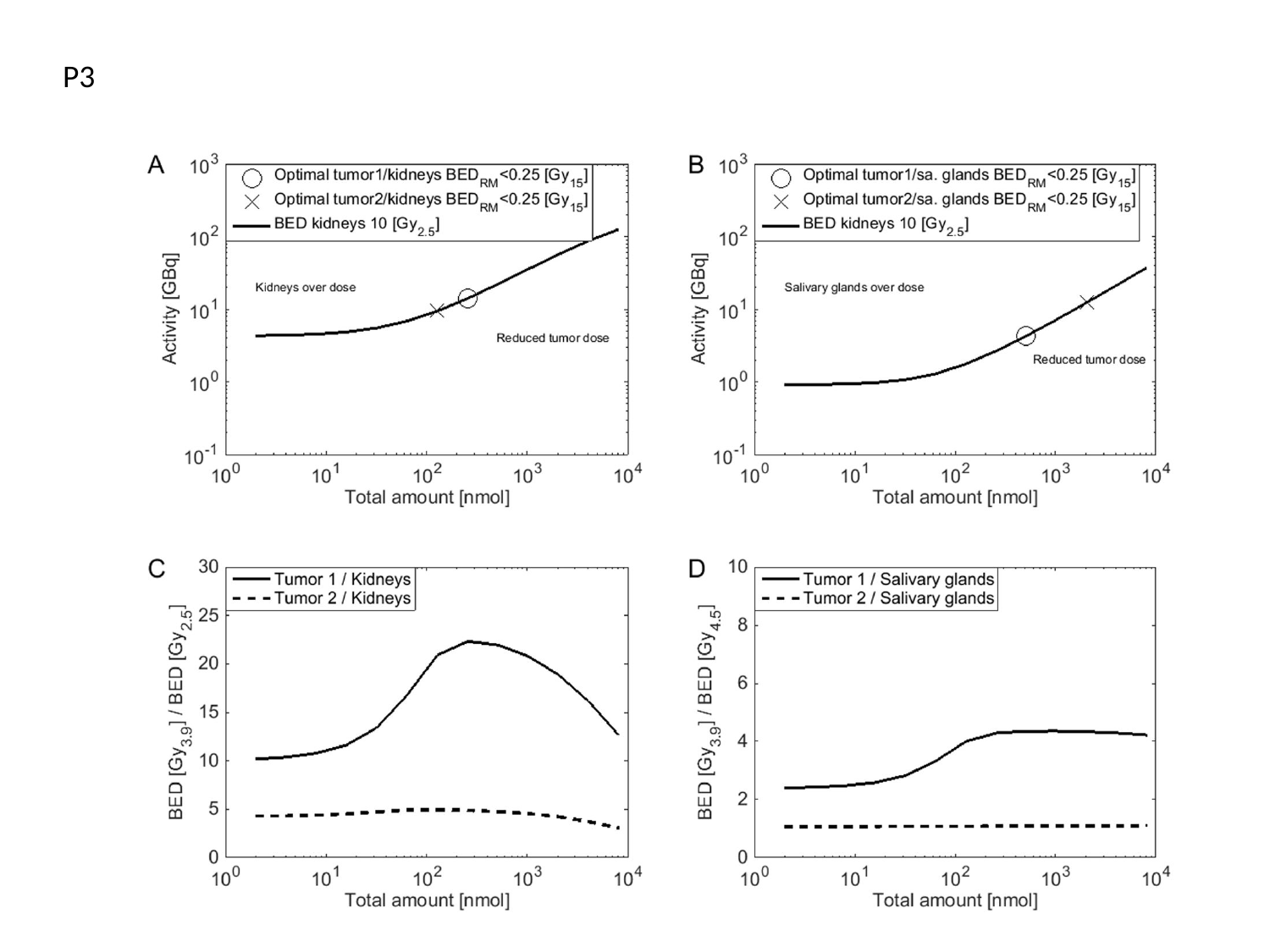

P3

## Slide 8
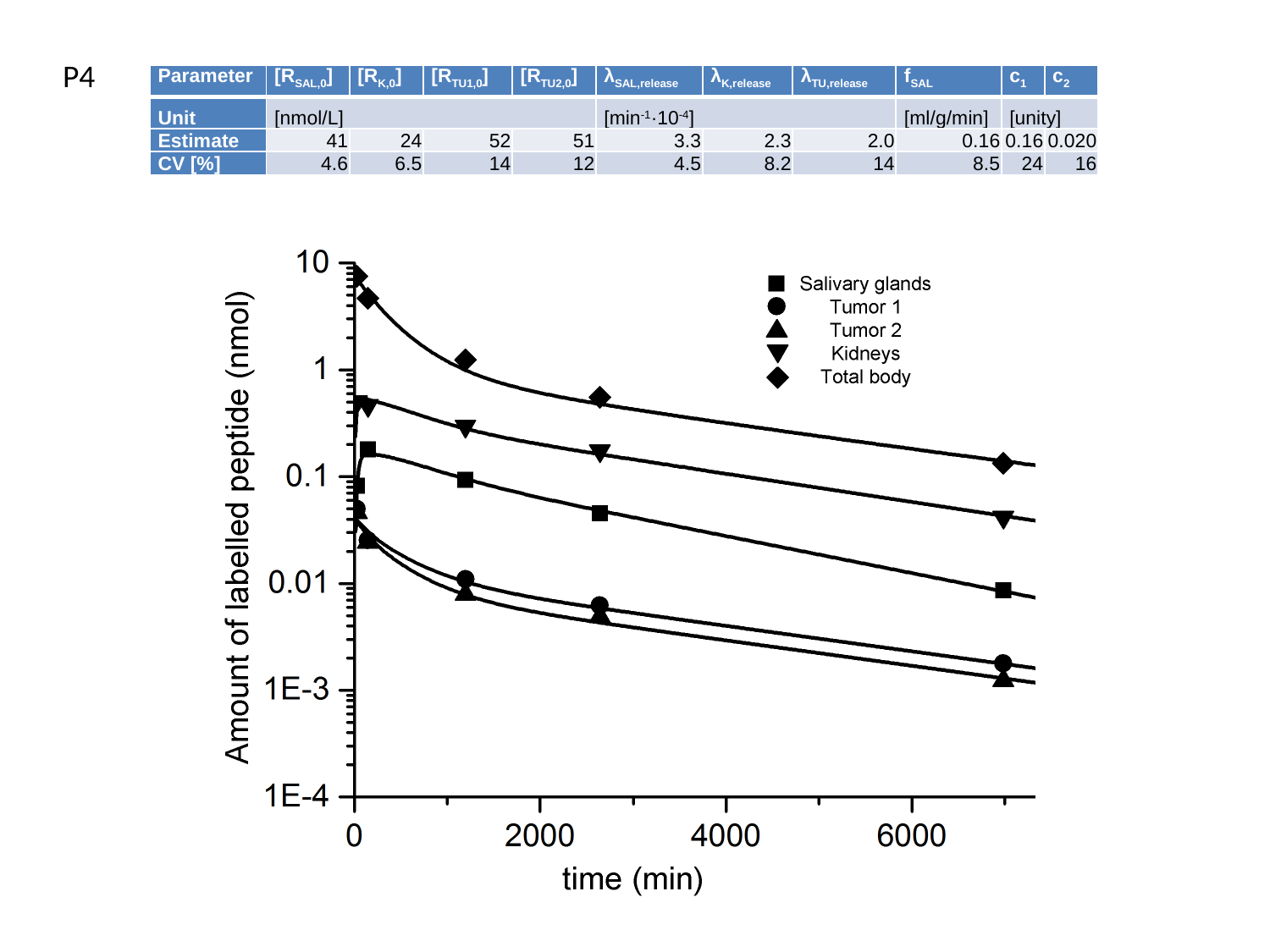

P4
| Parameter | [RSAL,0] | [RK,0] | [RTU1,0] | [RTU2,0] | λSAL,release | λK,release | λTU,release | fSAL | c1 | c2 |
| --- | --- | --- | --- | --- | --- | --- | --- | --- | --- | --- |
| Unit | [nmol/L] | | | | [min-1·10-4] | | | [ml/g/min] | [unity] | |
| Estimate | 41 | 24 | 52 | 51 | 3.3 | 2.3 | 2.0 | 0.16 | 0.16 | 0.020 |
| CV [%] | 4.6 | 6.5 | 14 | 12 | 4.5 | 8.2 | 14 | 8.5 | 24 | 16 |

## Slide 9
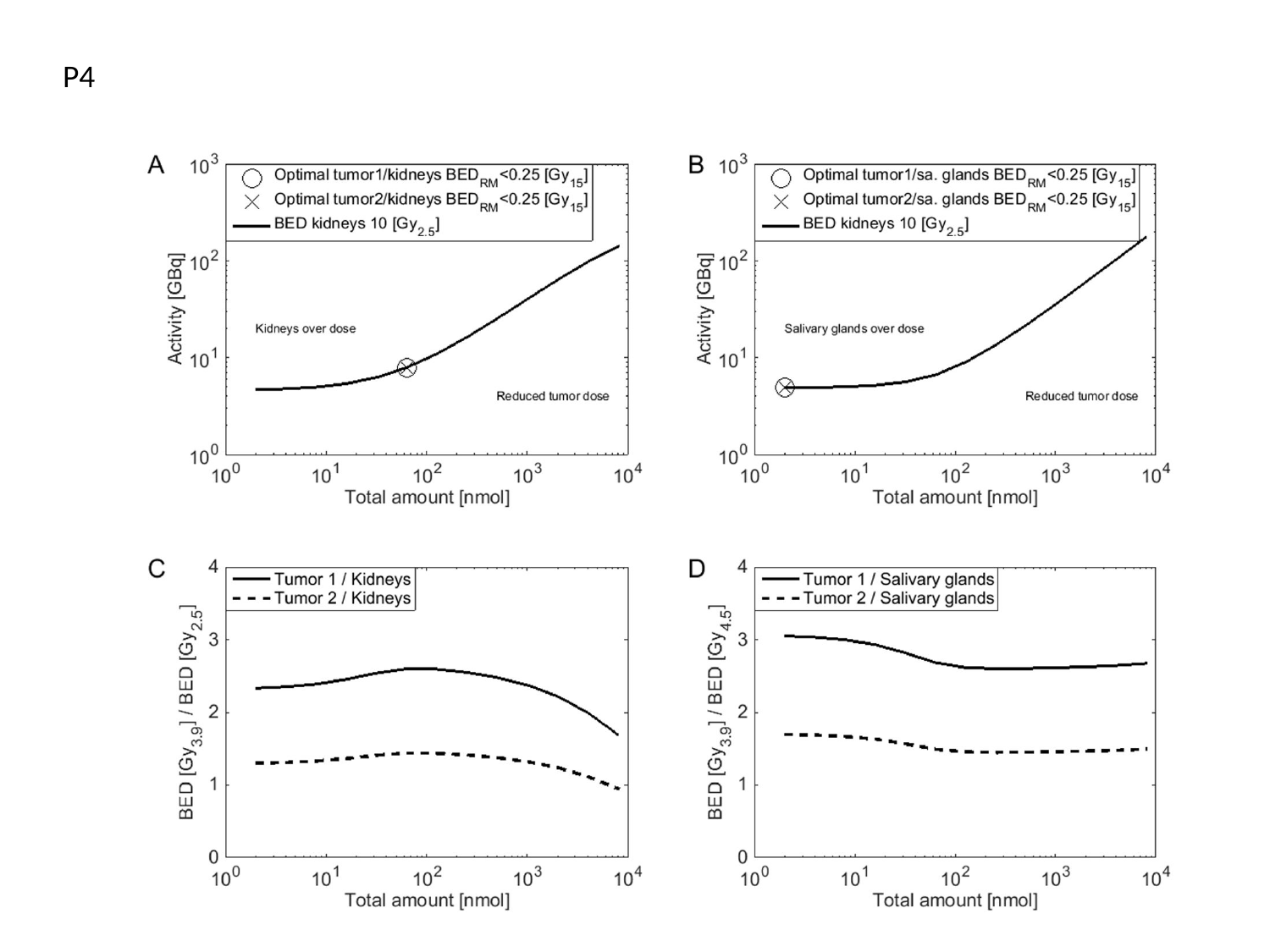

P4

## Slide 10
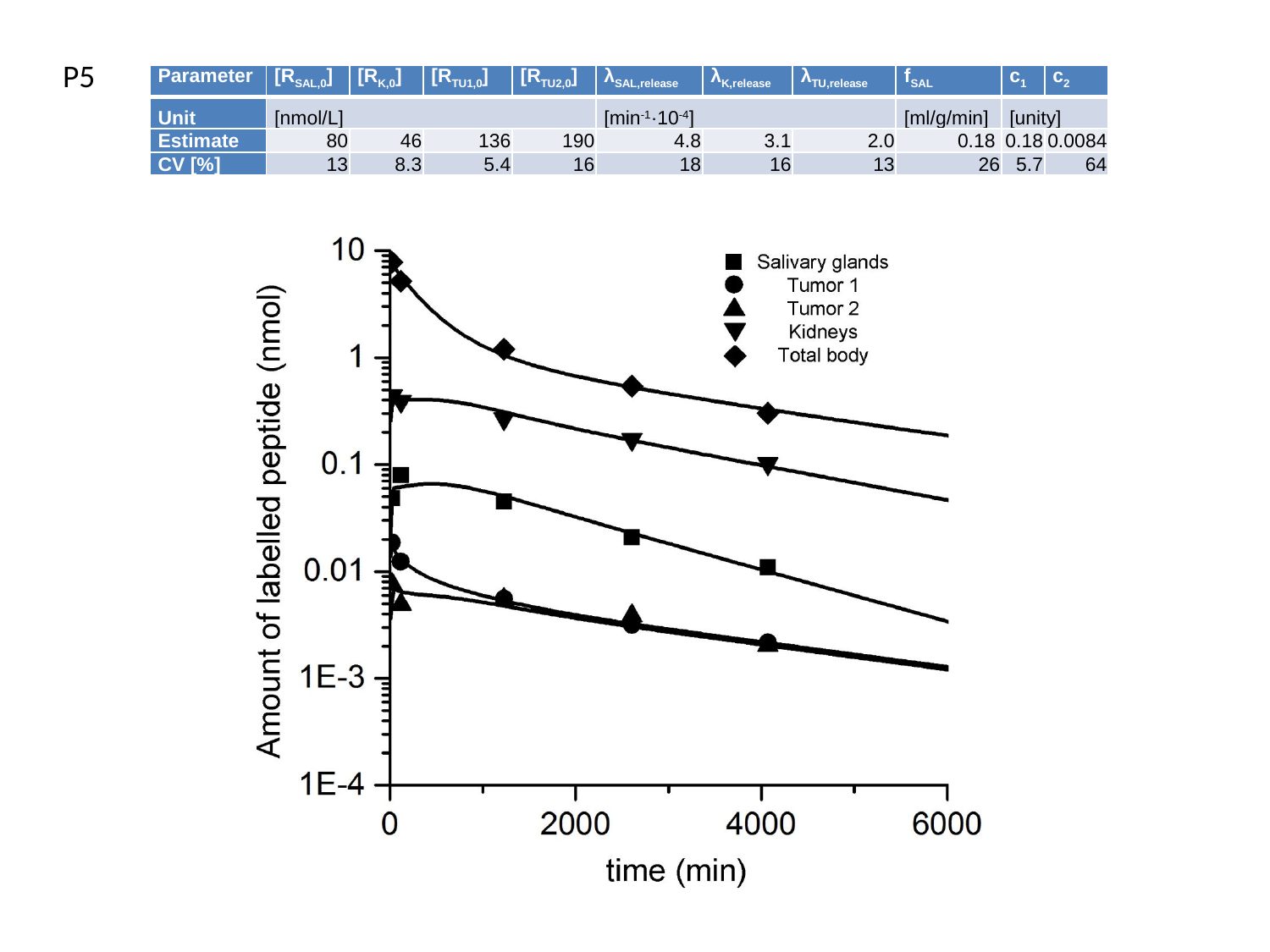

P5
| Parameter | [RSAL,0] | [RK,0] | [RTU1,0] | [RTU2,0] | λSAL,release | λK,release | λTU,release | fSAL | c1 | c2 |
| --- | --- | --- | --- | --- | --- | --- | --- | --- | --- | --- |
| Unit | [nmol/L] | | | | [min-1·10-4] | | | [ml/g/min] | [unity] | |
| Estimate | 80 | 46 | 136 | 190 | 4.8 | 3.1 | 2.0 | 0.18 | 0.18 | 0.0084 |
| CV [%] | 13 | 8.3 | 5.4 | 16 | 18 | 16 | 13 | 26 | 5.7 | 64 |

## Slide 11
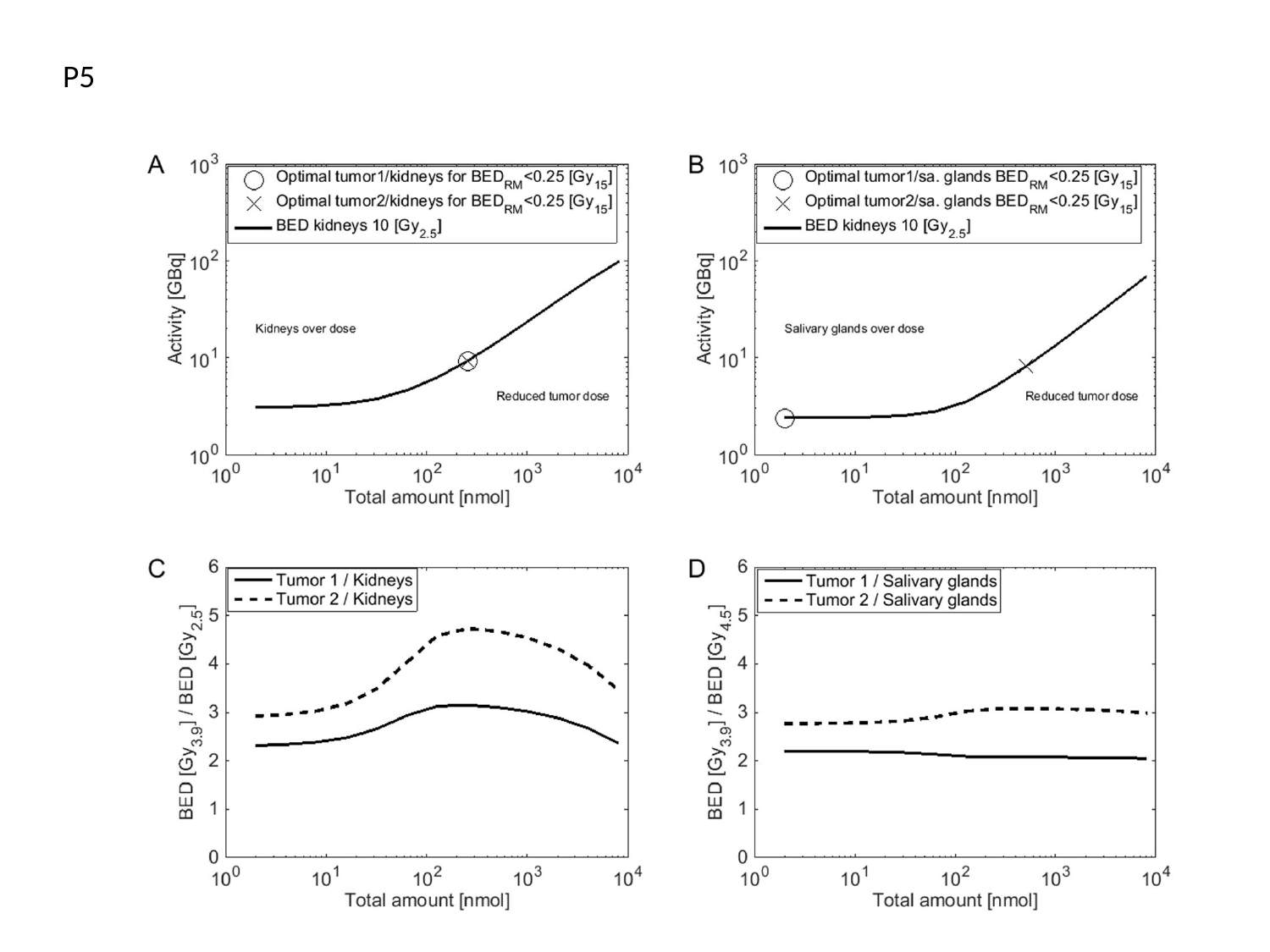

P5
