# Supplementary material for: Investigating the Effect of Ligand Amount and Injected Therapeutic Activity: A Simulation Study for 177Lu-Labeled PSMA-Targeting Peptides
Source: PLoS One. 2016 Sep 9;11(9):e0162303. doi: 10.1371/journal.pone.0162303 (PMC5017739; doi:10.1371/journal.pone.0162303)
Supplement: S3 File — BED simulations for all patients with varying blood flows to the tumor. (PPTX) [file pone.0162303.s003.pptx]

## Slide 1
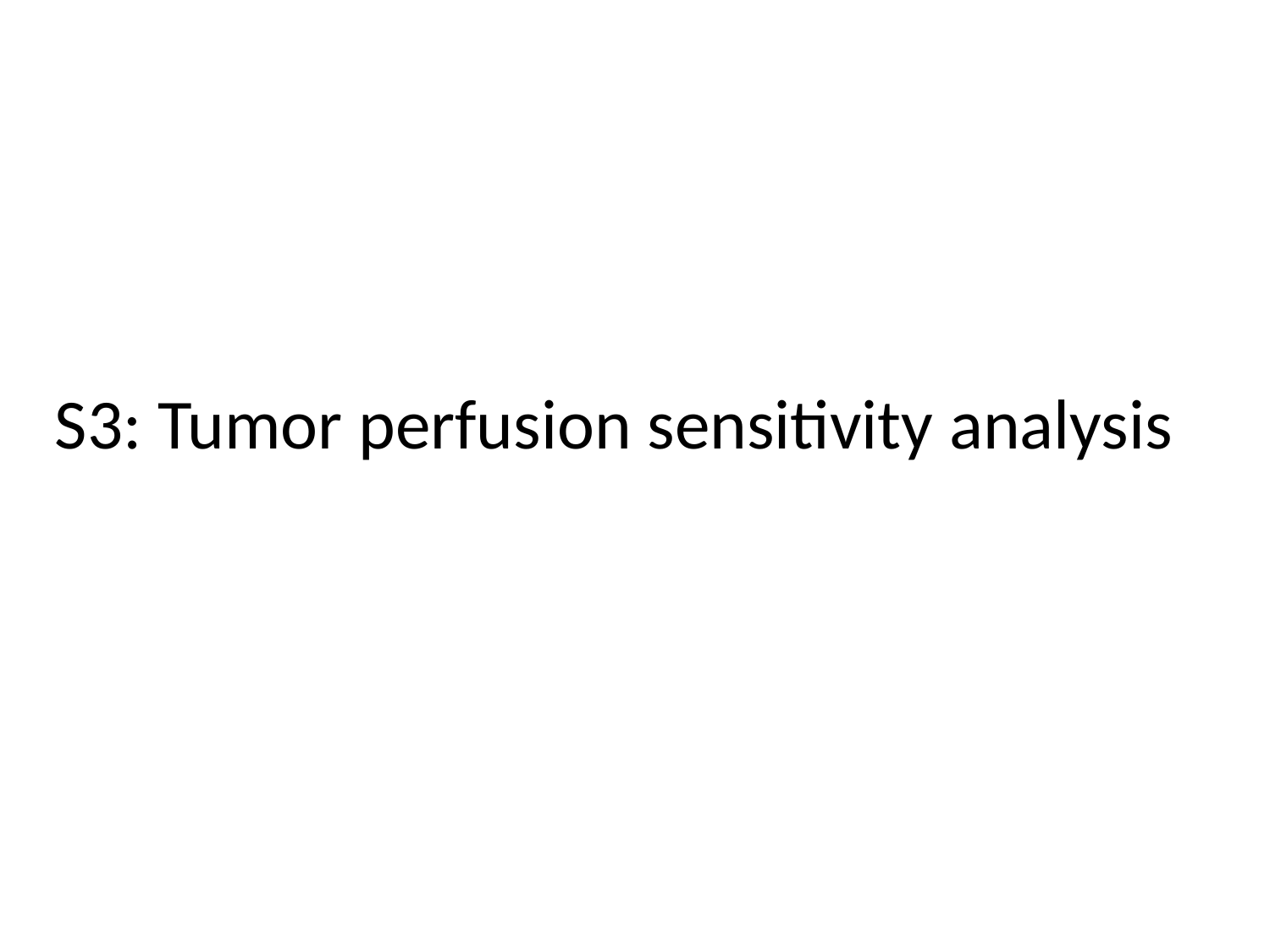

S3: Tumor perfusion sensitivity analysis

## Slide 2
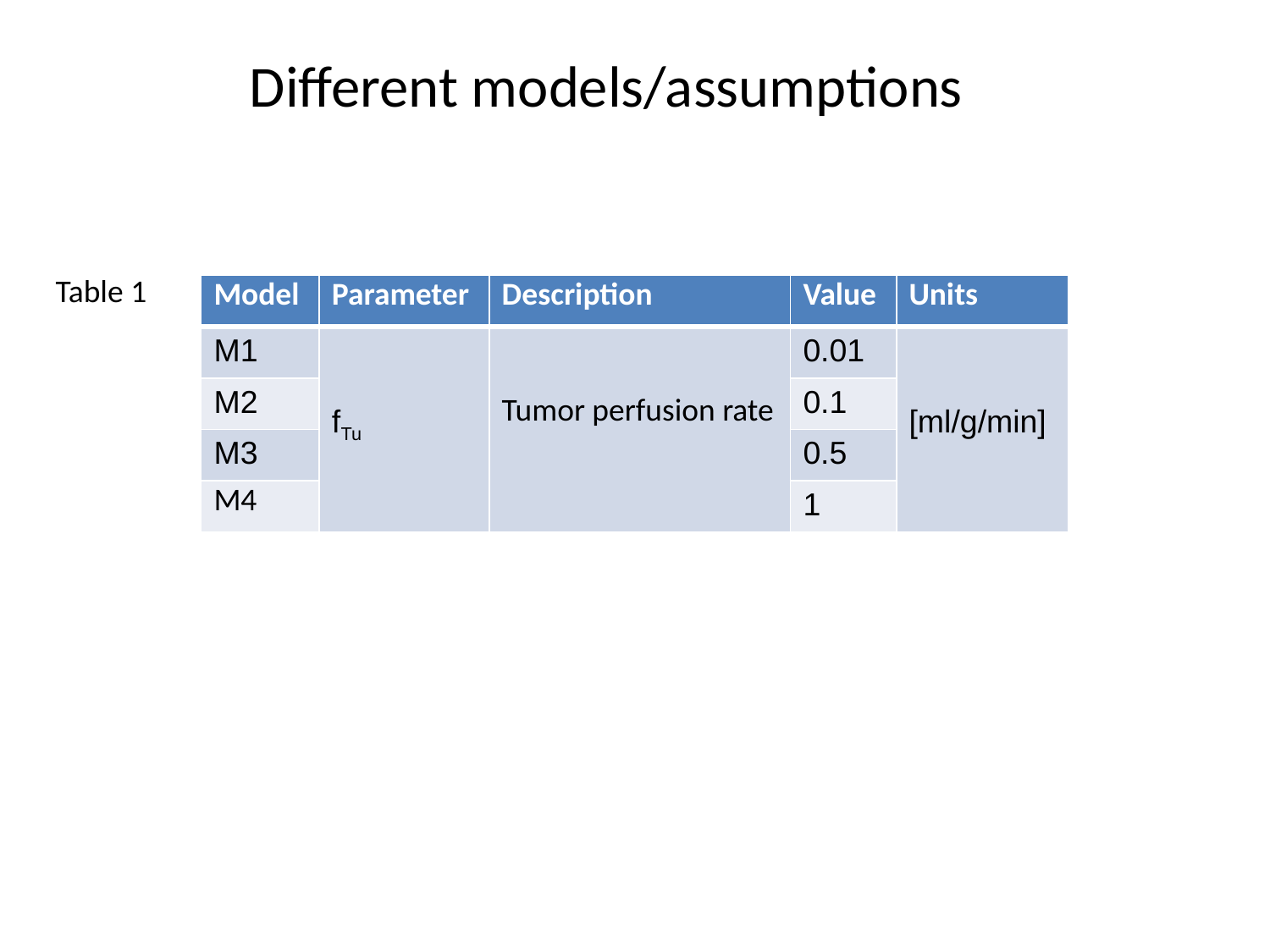

Different models/assumptions
Table 1
| Model | Parameter | Description | Value | Units |
| --- | --- | --- | --- | --- |
| M1 | fTu | Tumor perfusion rate | 0.01 | [ml/g/min] |
| M2 | | | 0.1 | |
| M3 | | | 0.5 | |
| M4 | | | 1 | |

## Slide 3
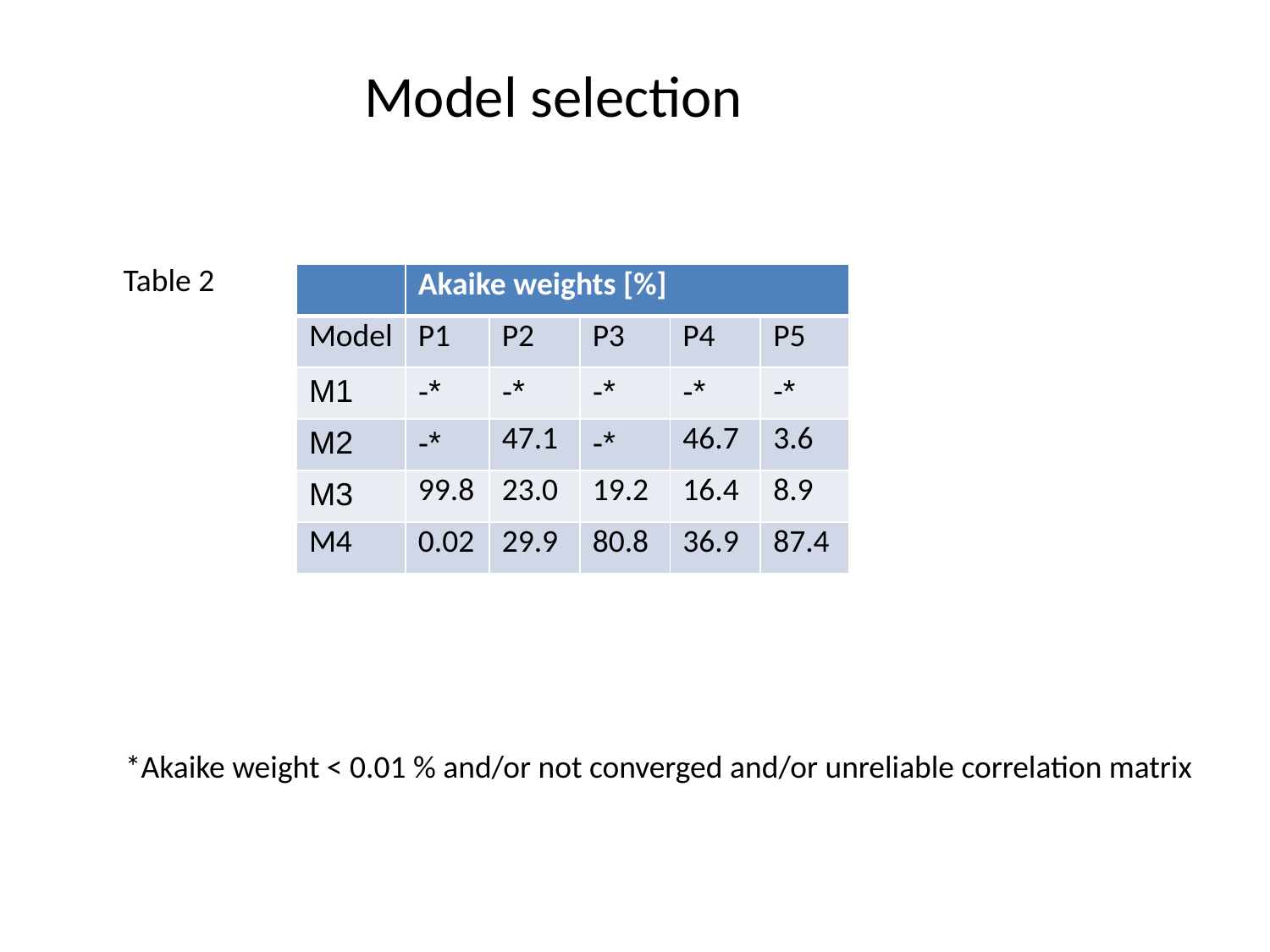

Model selection
Table 2
| | Akaike weights [%] | | | | |
| --- | --- | --- | --- | --- | --- |
| Model | P1 | P2 | P3 | P4 | P5 |
| M1 | -\* | -\* | -\* | -\* | -\* |
| M2 | -\* | 47.1 | -\* | 46.7 | 3.6 |
| M3 | 99.8 | 23.0 | 19.2 | 16.4 | 8.9 |
| M4 | 0.02 | 29.9 | 80.8 | 36.9 | 87.4 |
*Akaike weight < 0.01 % and/or not converged and/or unreliable correlation matrix

## Slide 4
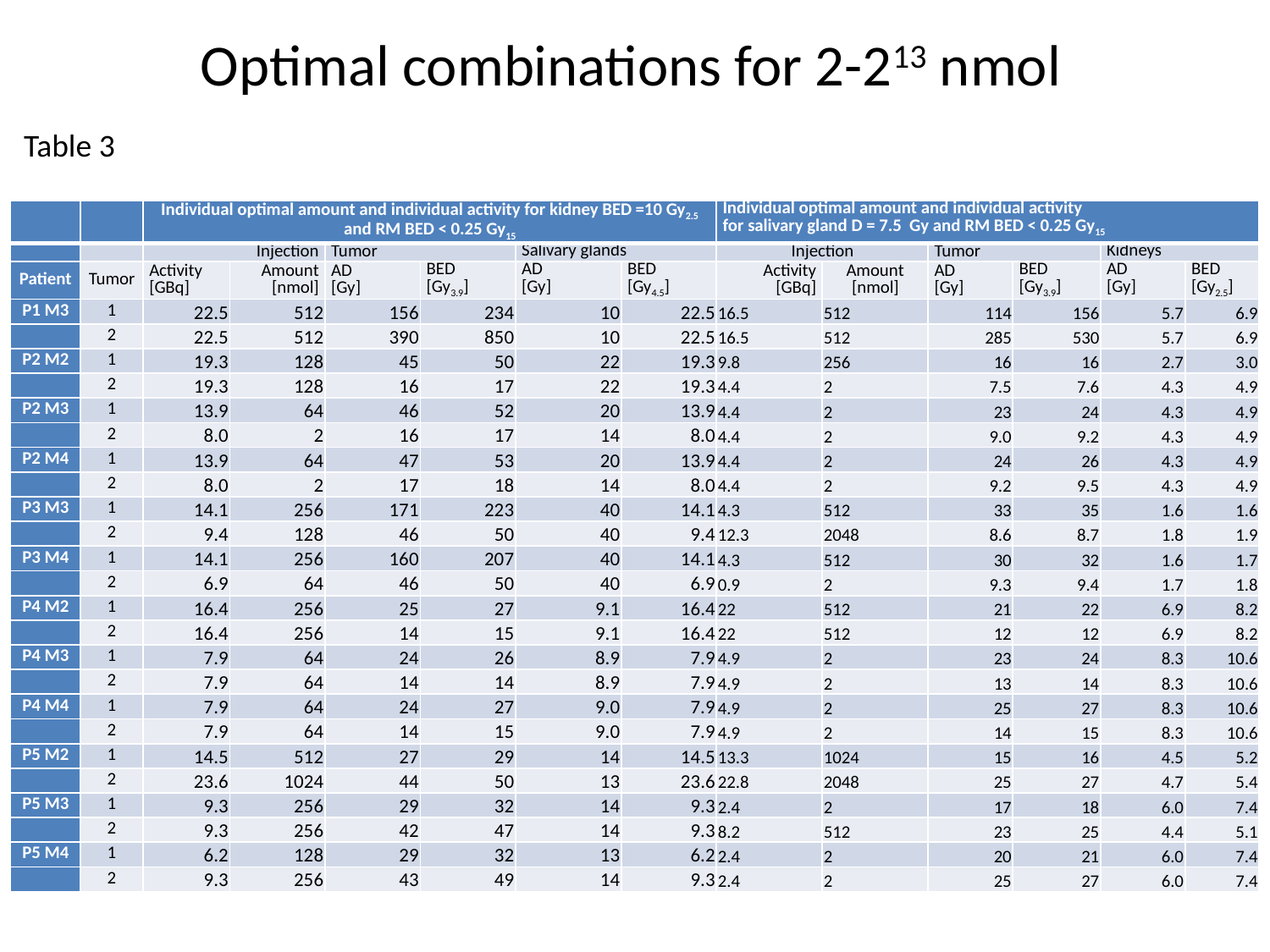

Optimal combinations for 2-213 nmol
Table 3
| | | Individual optimal amount and individual activity for kidney BED =10 Gy2.5 and RM BED < 0.25 Gy15 | | | | | | Individual optimal amount and individual activity for salivary gland D = 7.5 Gy and RM BED < 0.25 Gy15 | | | | | |
| --- | --- | --- | --- | --- | --- | --- | --- | --- | --- | --- | --- | --- | --- |
| | | Injection | | Tumor | | Salivary glands | | Injection | | Tumor | | Kidneys | |
| Patient | Tumor | Activity [GBq] | Amount [nmol] | AD [Gy] | BED [Gy3.9] | AD [Gy] | BED [Gy4.5] | Activity [GBq] | Amount [nmol] | AD [Gy] | BED [Gy3.9] | AD [Gy] | BED [Gy2.5] |
| P1 M3 | 1 | 22.5 | 512 | 156 | 234 | 10 | 22.5 | 16.5 | 512 | 114 | 156 | 5.7 | 6.9 |
| | 2 | 22.5 | 512 | 390 | 850 | 10 | 22.5 | 16.5 | 512 | 285 | 530 | 5.7 | 6.9 |
| P2 M2 | 1 | 19.3 | 128 | 45 | 50 | 22 | 19.3 | 9.8 | 256 | 16 | 16 | 2.7 | 3.0 |
| | 2 | 19.3 | 128 | 16 | 17 | 22 | 19.3 | 4.4 | 2 | 7.5 | 7.6 | 4.3 | 4.9 |
| P2 M3 | 1 | 13.9 | 64 | 46 | 52 | 20 | 13.9 | 4.4 | 2 | 23 | 24 | 4.3 | 4.9 |
| | 2 | 8.0 | 2 | 16 | 17 | 14 | 8.0 | 4.4 | 2 | 9.0 | 9.2 | 4.3 | 4.9 |
| P2 M4 | 1 | 13.9 | 64 | 47 | 53 | 20 | 13.9 | 4.4 | 2 | 24 | 26 | 4.3 | 4.9 |
| | 2 | 8.0 | 2 | 17 | 18 | 14 | 8.0 | 4.4 | 2 | 9.2 | 9.5 | 4.3 | 4.9 |
| P3 M3 | 1 | 14.1 | 256 | 171 | 223 | 40 | 14.1 | 4.3 | 512 | 33 | 35 | 1.6 | 1.6 |
| | 2 | 9.4 | 128 | 46 | 50 | 40 | 9.4 | 12.3 | 2048 | 8.6 | 8.7 | 1.8 | 1.9 |
| P3 M4 | 1 | 14.1 | 256 | 160 | 207 | 40 | 14.1 | 4.3 | 512 | 30 | 32 | 1.6 | 1.7 |
| | 2 | 6.9 | 64 | 46 | 50 | 40 | 6.9 | 0.9 | 2 | 9.3 | 9.4 | 1.7 | 1.8 |
| P4 M2 | 1 | 16.4 | 256 | 25 | 27 | 9.1 | 16.4 | 22 | 512 | 21 | 22 | 6.9 | 8.2 |
| | 2 | 16.4 | 256 | 14 | 15 | 9.1 | 16.4 | 22 | 512 | 12 | 12 | 6.9 | 8.2 |
| P4 M3 | 1 | 7.9 | 64 | 24 | 26 | 8.9 | 7.9 | 4.9 | 2 | 23 | 24 | 8.3 | 10.6 |
| | 2 | 7.9 | 64 | 14 | 14 | 8.9 | 7.9 | 4.9 | 2 | 13 | 14 | 8.3 | 10.6 |
| P4 M4 | 1 | 7.9 | 64 | 24 | 27 | 9.0 | 7.9 | 4.9 | 2 | 25 | 27 | 8.3 | 10.6 |
| | 2 | 7.9 | 64 | 14 | 15 | 9.0 | 7.9 | 4.9 | 2 | 14 | 15 | 8.3 | 10.6 |
| P5 M2 | 1 | 14.5 | 512 | 27 | 29 | 14 | 14.5 | 13.3 | 1024 | 15 | 16 | 4.5 | 5.2 |
| | 2 | 23.6 | 1024 | 44 | 50 | 13 | 23.6 | 22.8 | 2048 | 25 | 27 | 4.7 | 5.4 |
| P5 M3 | 1 | 9.3 | 256 | 29 | 32 | 14 | 9.3 | 2.4 | 2 | 17 | 18 | 6.0 | 7.4 |
| | 2 | 9.3 | 256 | 42 | 47 | 14 | 9.3 | 8.2 | 512 | 23 | 25 | 4.4 | 5.1 |
| P5 M4 | 1 | 6.2 | 128 | 29 | 32 | 13 | 6.2 | 2.4 | 2 | 20 | 21 | 6.0 | 7.4 |
| | 2 | 9.3 | 256 | 43 | 49 | 14 | 9.3 | 2.4 | 2 | 25 | 27 | 6.0 | 7.4 |

## Slide 5
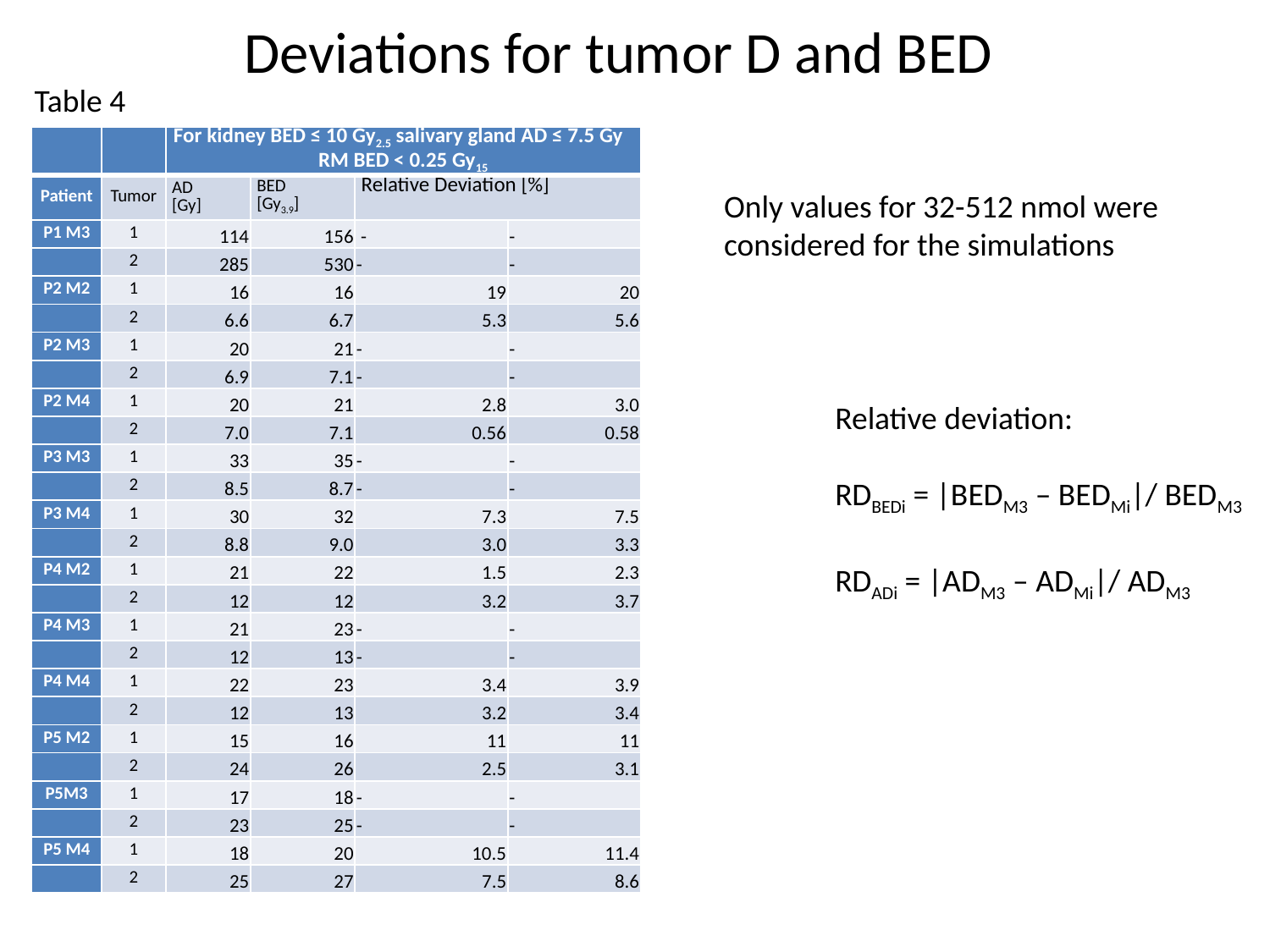

Deviations for tumor D and BED
Table 4
| | | For kidney BED ≤ 10 Gy2.5 salivary gland AD ≤ 7.5 Gy RM BED < 0.25 Gy15 | | | |
| --- | --- | --- | --- | --- | --- |
| Patient | Tumor | AD [Gy] | BED [Gy3.9] | Relative Deviation [%] | |
| P1 M3 | 1 | 114 | 156 | - | - |
| | 2 | 285 | 530 | - | - |
| P2 M2 | 1 | 16 | 16 | 19 | 20 |
| | 2 | 6.6 | 6.7 | 5.3 | 5.6 |
| P2 M3 | 1 | 20 | 21 | - | - |
| | 2 | 6.9 | 7.1 | - | - |
| P2 M4 | 1 | 20 | 21 | 2.8 | 3.0 |
| | 2 | 7.0 | 7.1 | 0.56 | 0.58 |
| P3 M3 | 1 | 33 | 35 | - | - |
| | 2 | 8.5 | 8.7 | - | - |
| P3 M4 | 1 | 30 | 32 | 7.3 | 7.5 |
| | 2 | 8.8 | 9.0 | 3.0 | 3.3 |
| P4 M2 | 1 | 21 | 22 | 1.5 | 2.3 |
| | 2 | 12 | 12 | 3.2 | 3.7 |
| P4 M3 | 1 | 21 | 23 | - | - |
| | 2 | 12 | 13 | - | - |
| P4 M4 | 1 | 22 | 23 | 3.4 | 3.9 |
| | 2 | 12 | 13 | 3.2 | 3.4 |
| P5 M2 | 1 | 15 | 16 | 11 | 11 |
| | 2 | 24 | 26 | 2.5 | 3.1 |
| P5M3 | 1 | 17 | 18 | - | - |
| | 2 | 23 | 25 | - | - |
| P5 M4 | 1 | 18 | 20 | 10.5 | 11.4 |
| | 2 | 25 | 27 | 7.5 | 8.6 |
Only values for 32-512 nmol were
considered for the simulations
Relative deviation:
RDBEDi = |BEDM3 – BEDMi|/ BEDM3
RDADi = |ADM3 – ADMi|/ ADM3

## Slide 6
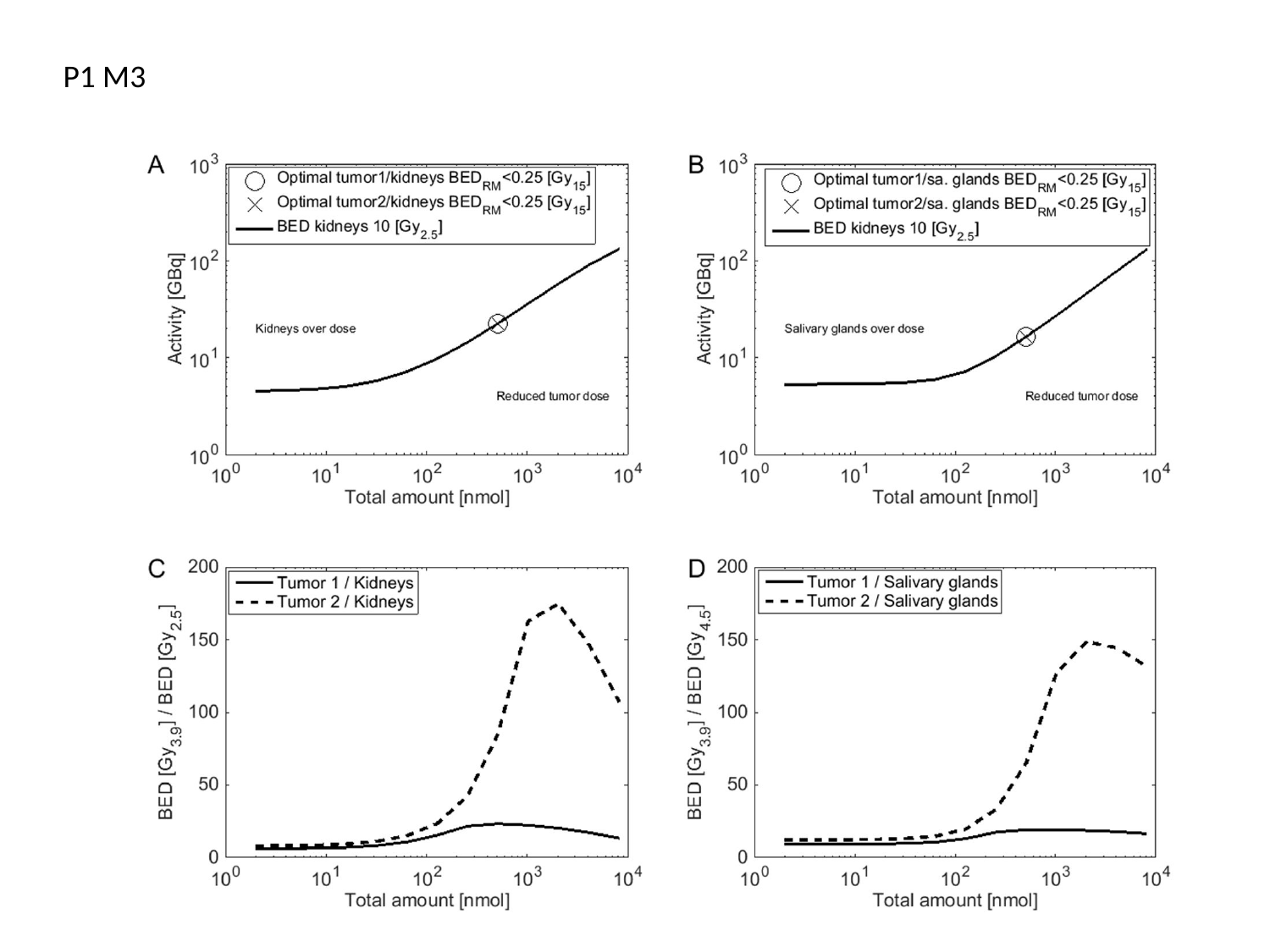

P1 M3

## Slide 7
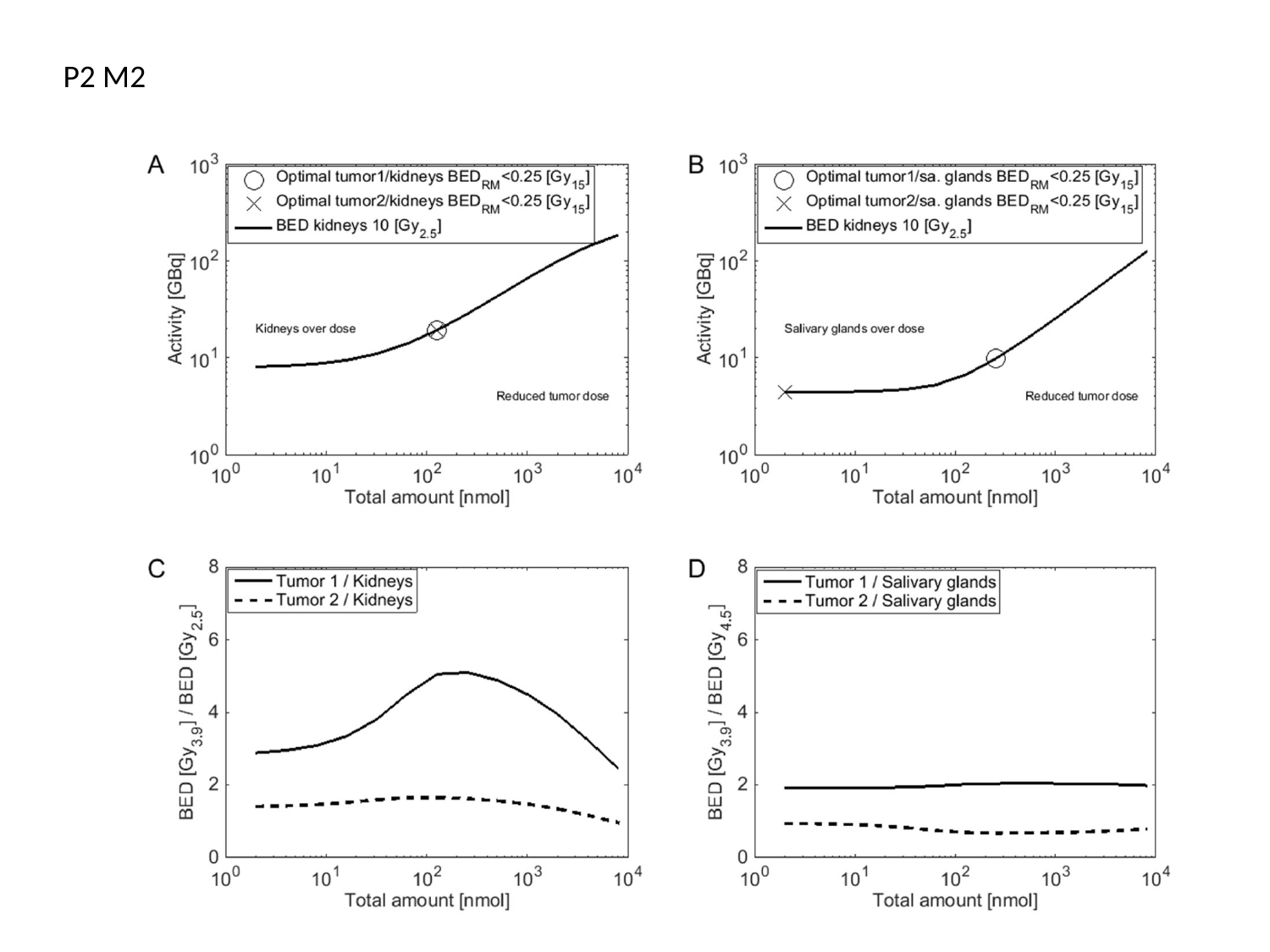

P2 M2

## Slide 8
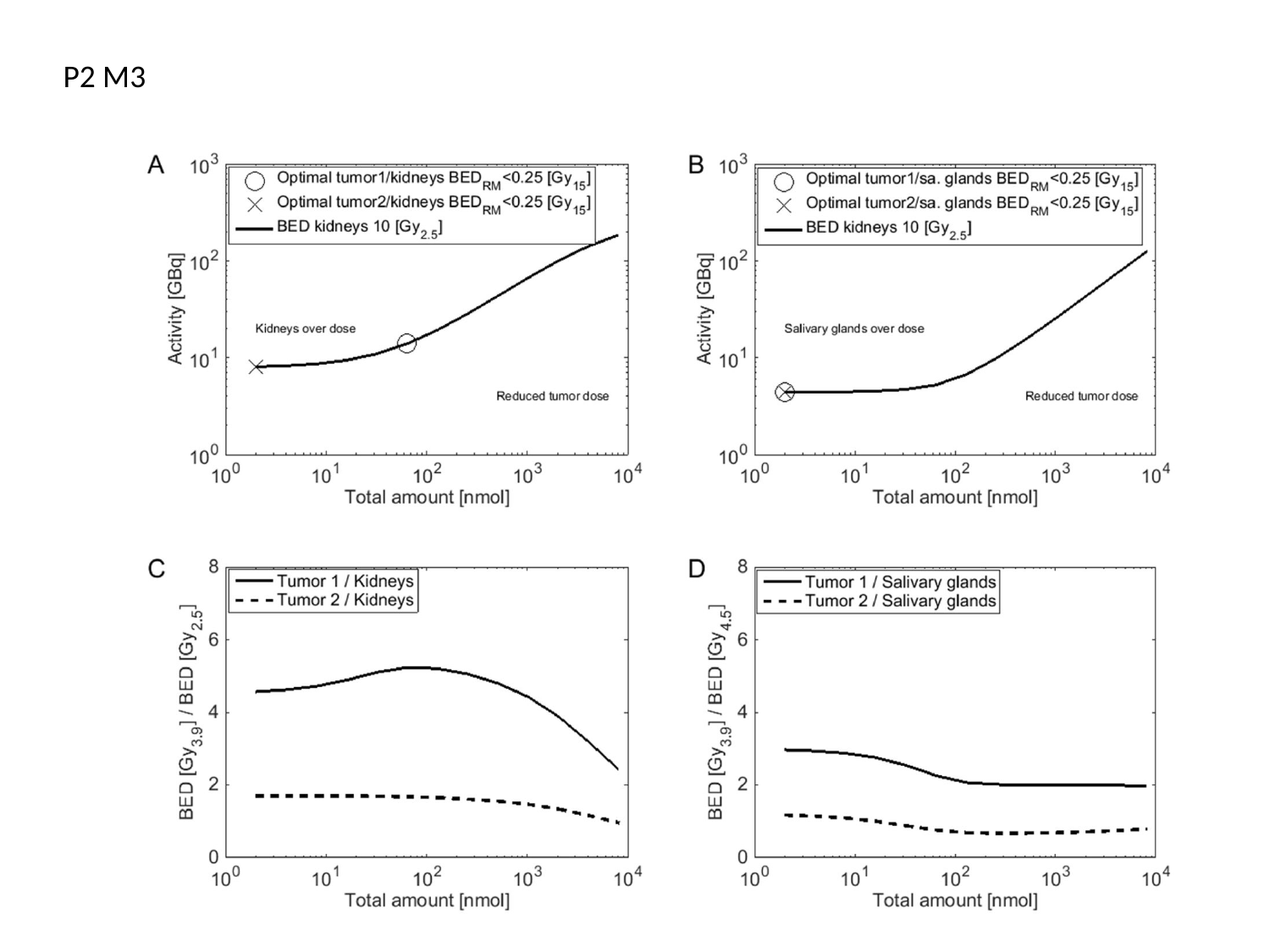

P2 M3

## Slide 9
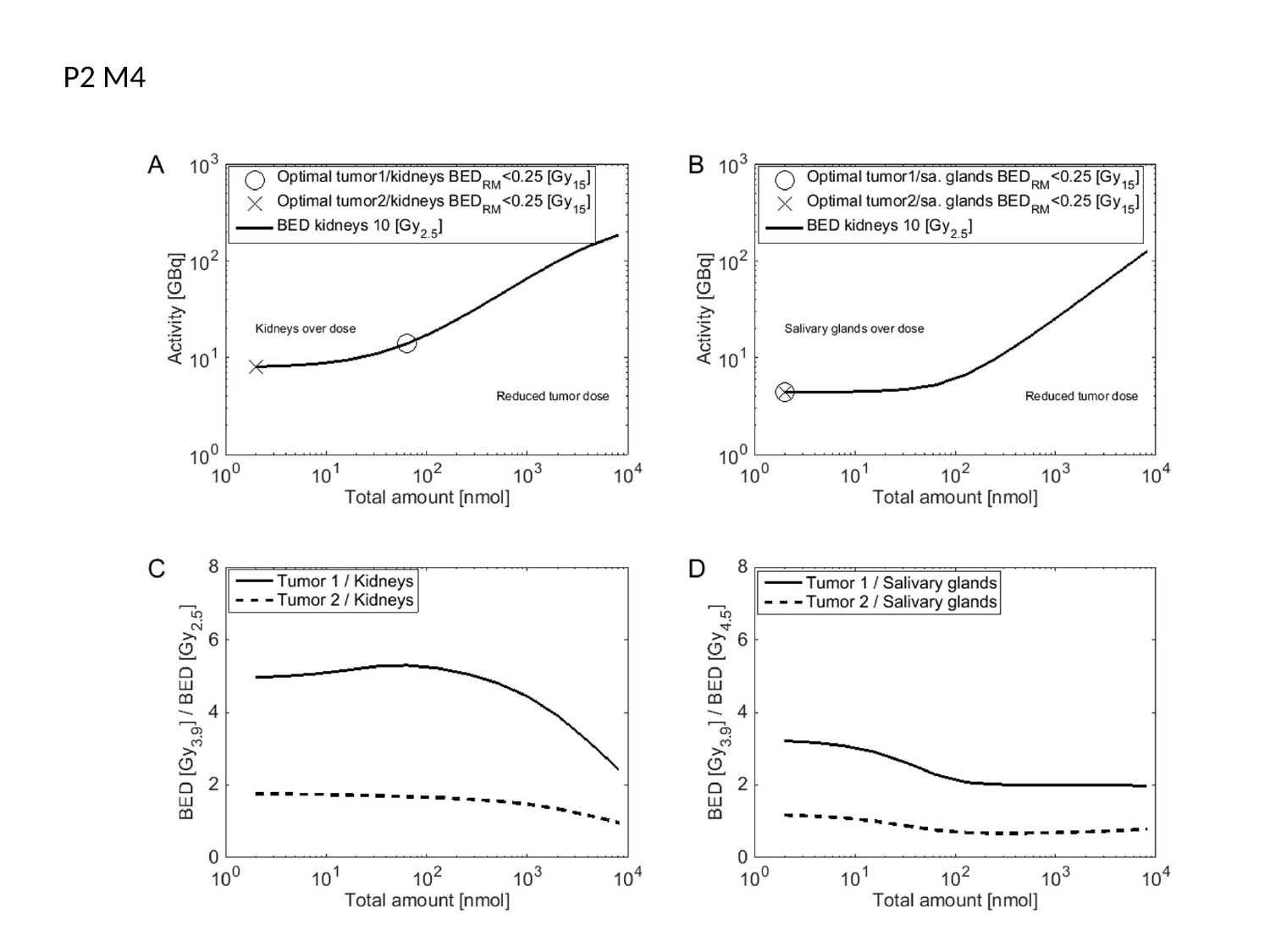

P2 M4

## Slide 10
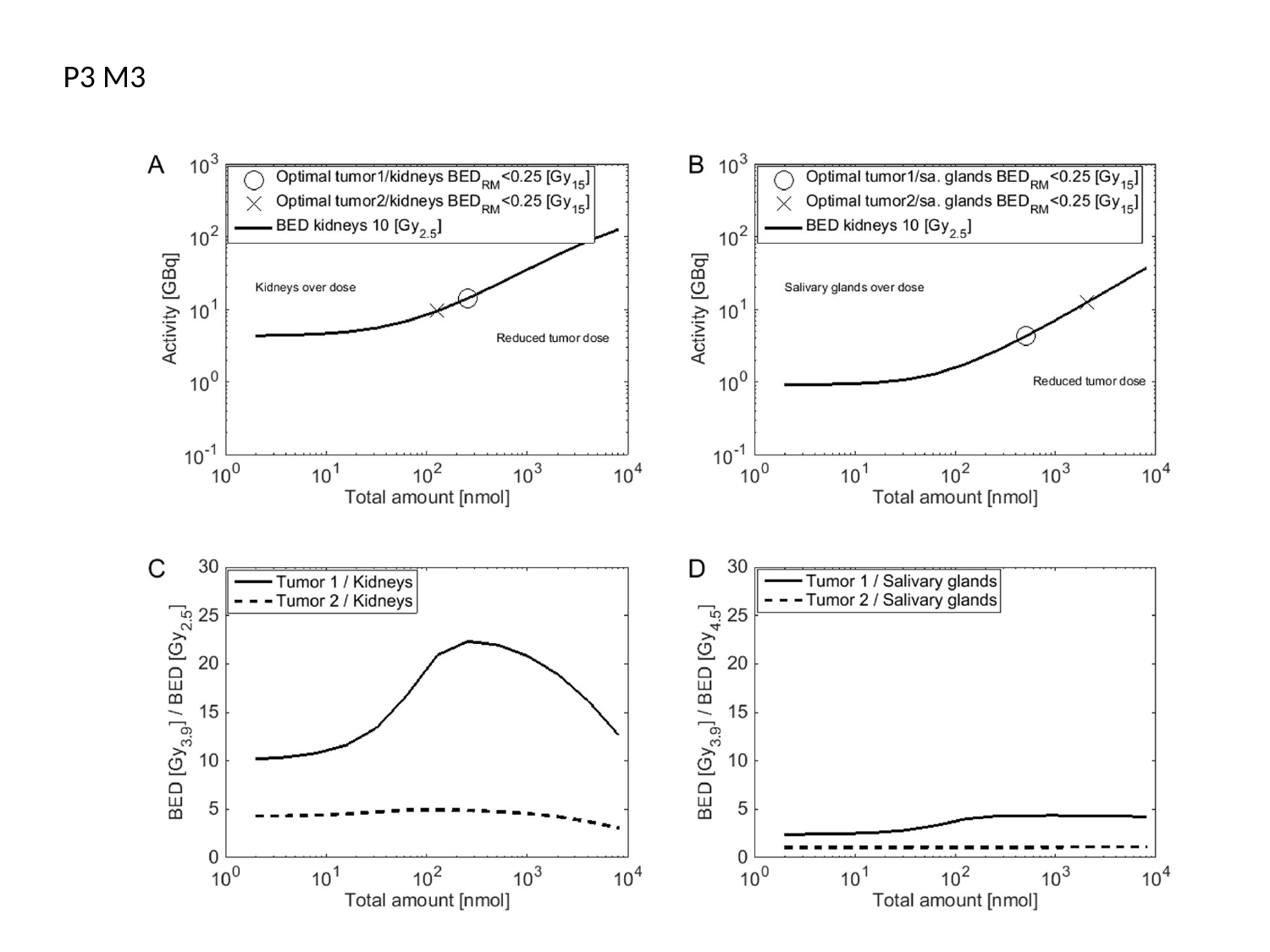

P3 M3

## Slide 11
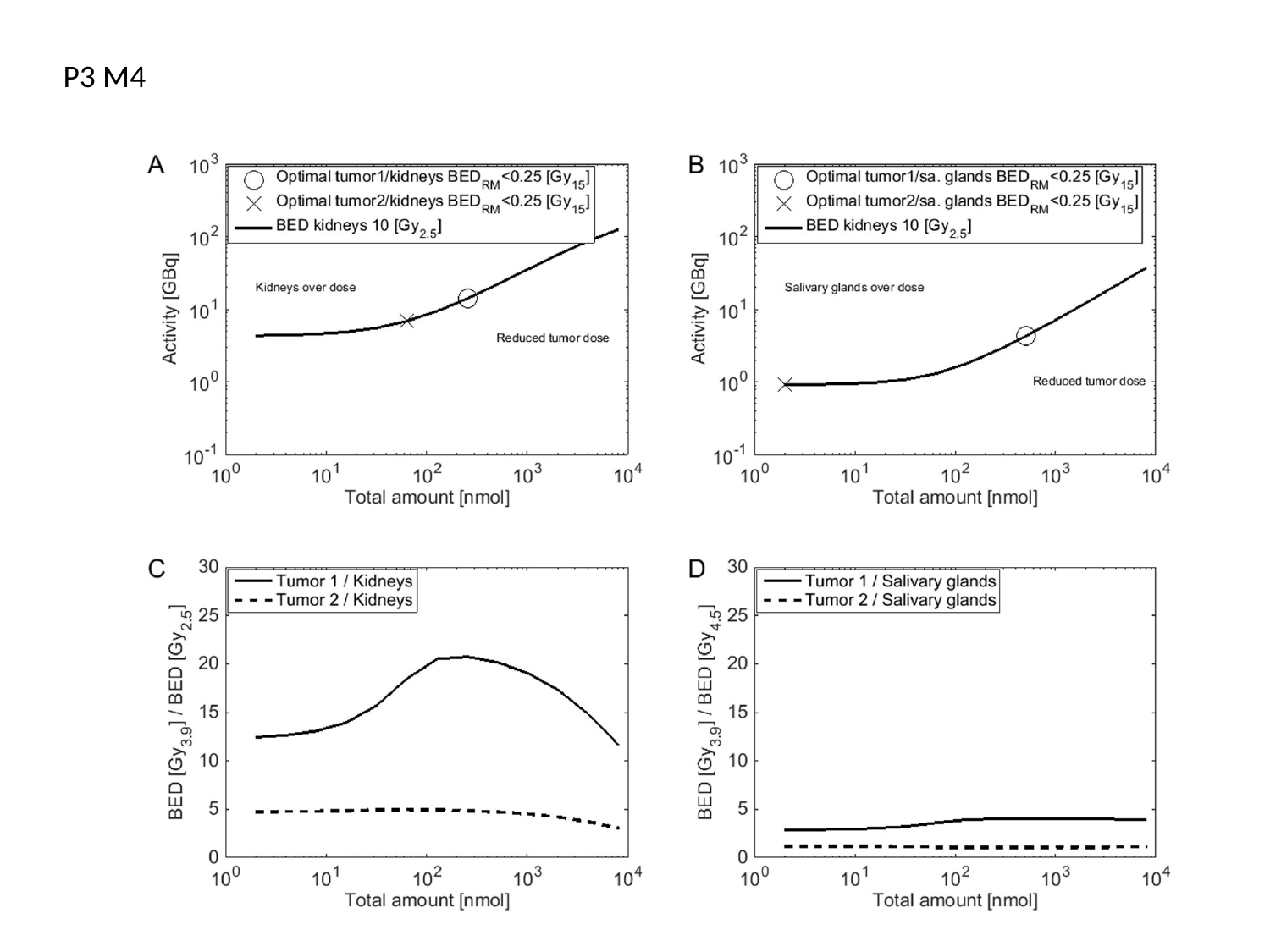

P3 M4

## Slide 12
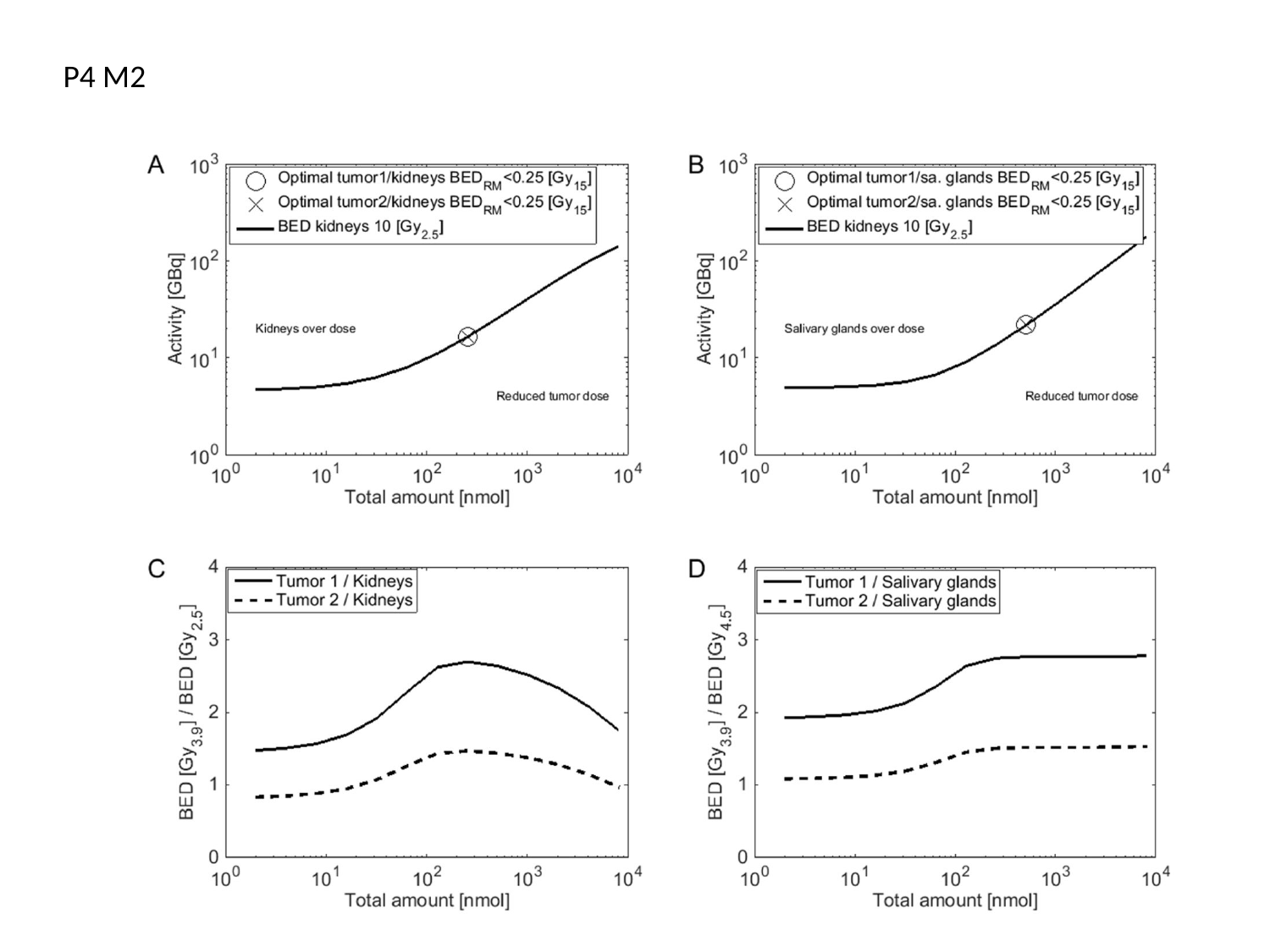

P4 M2

## Slide 13
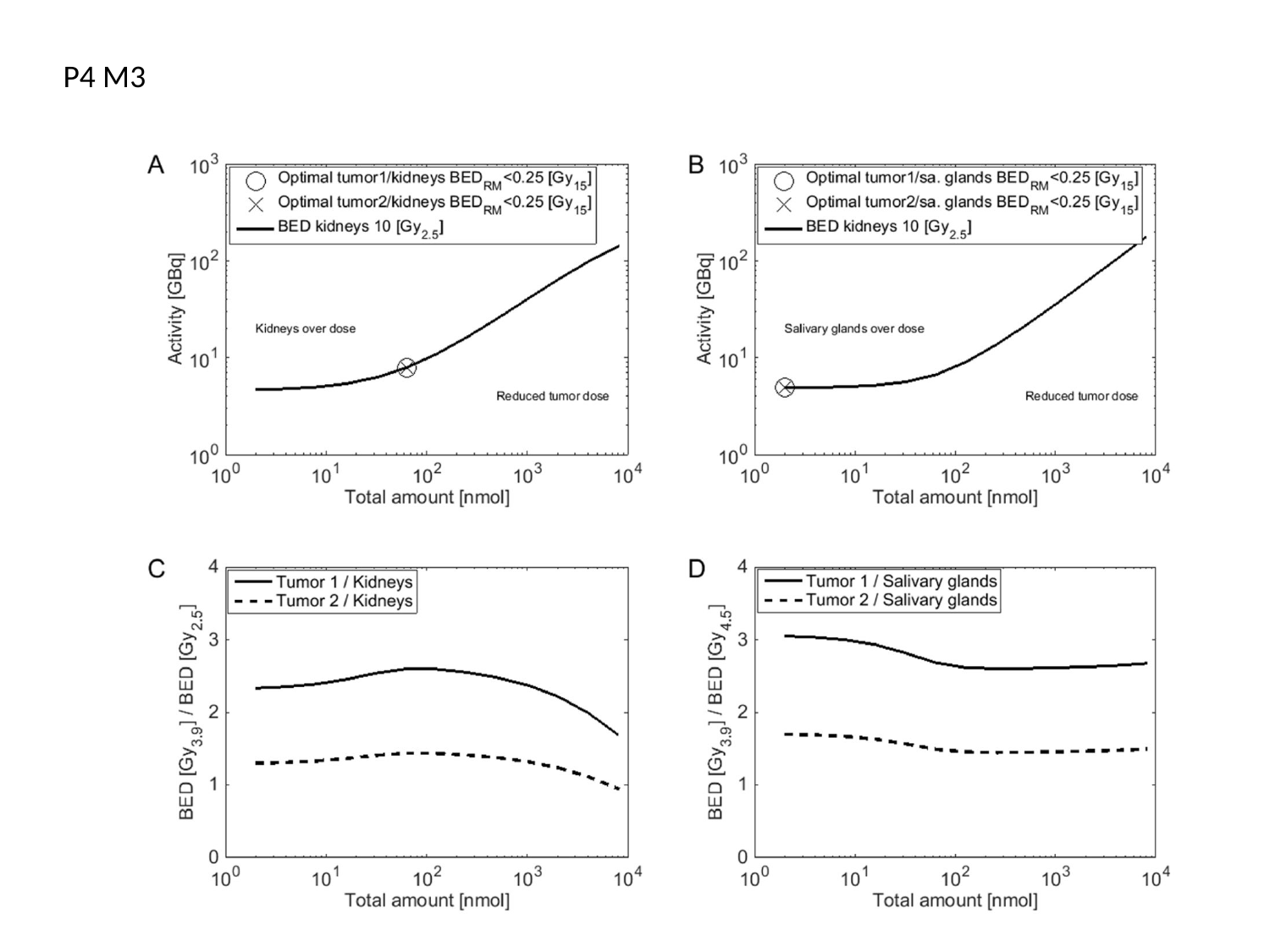

P4 M3

## Slide 14
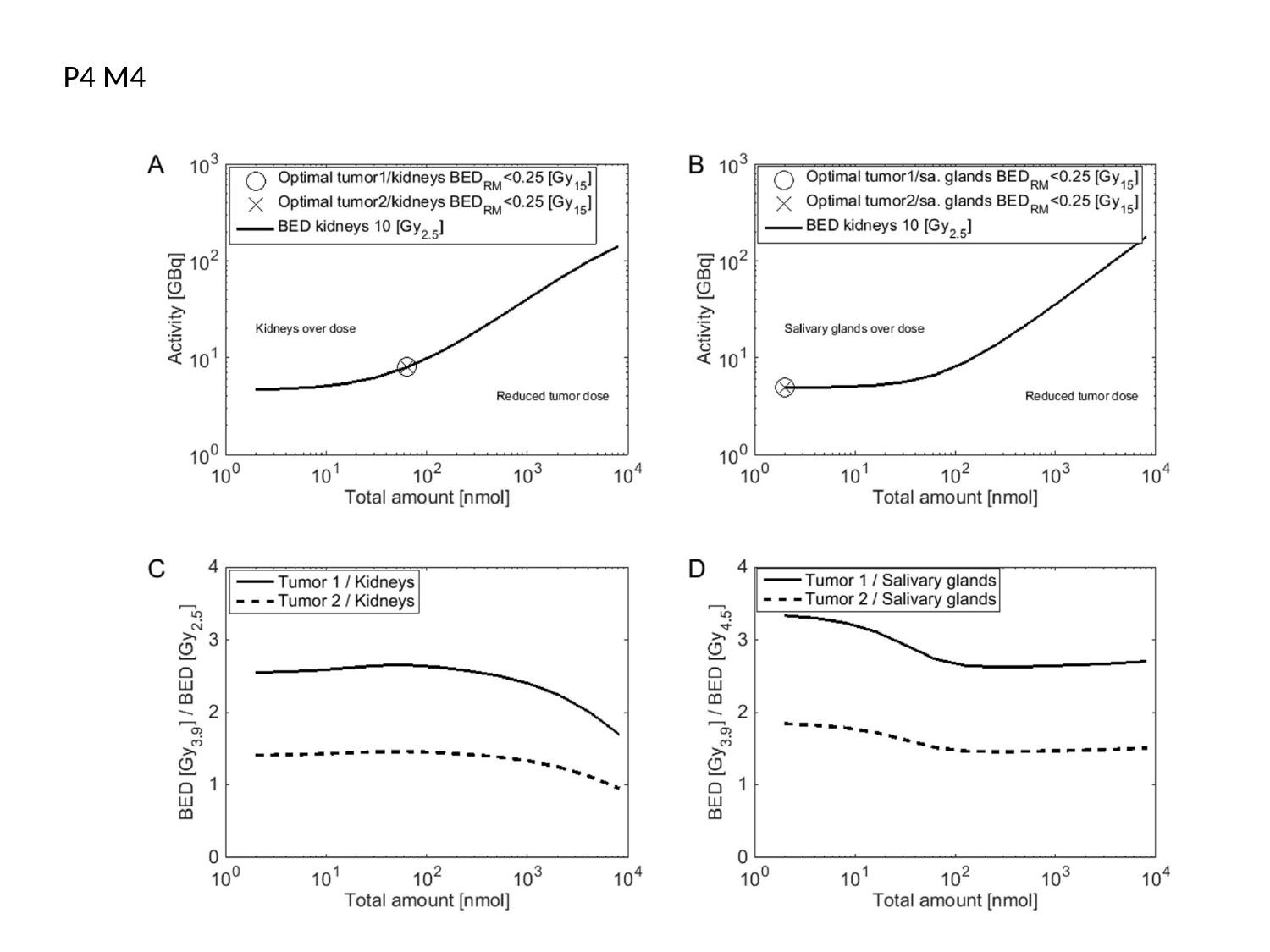

P4 M4

## Slide 15
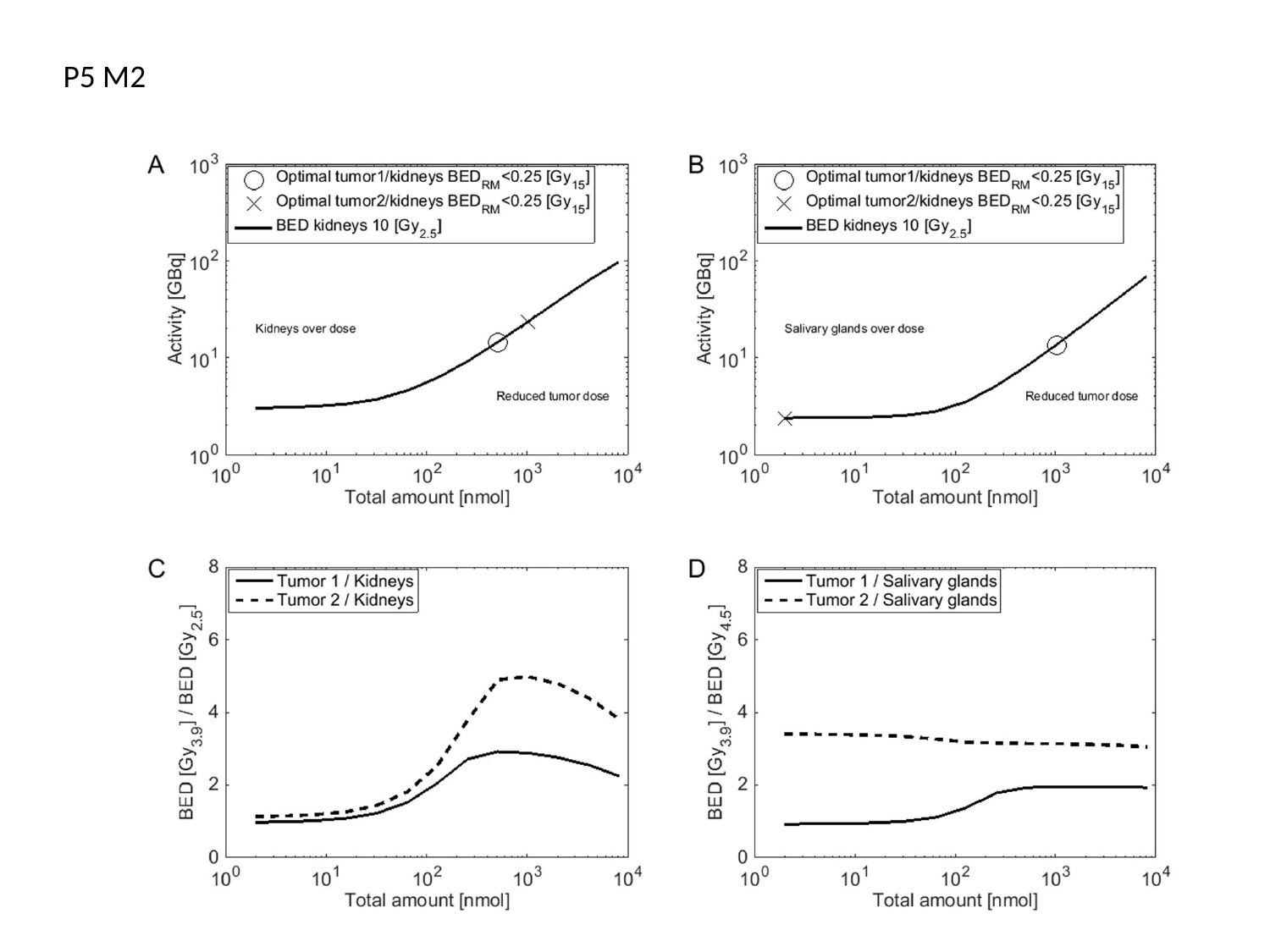

P5 M2

## Slide 16
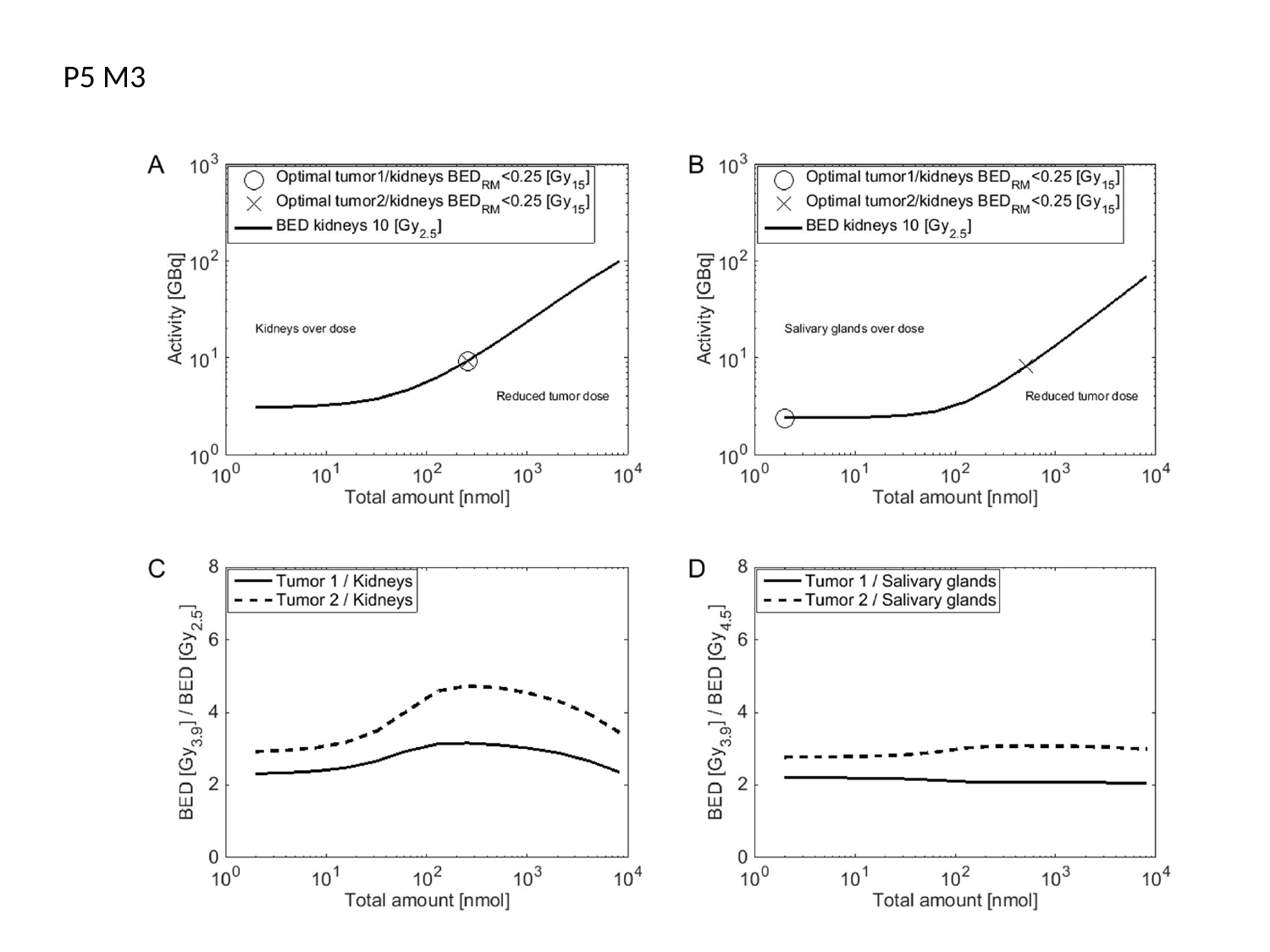

P5 M3

## Slide 17
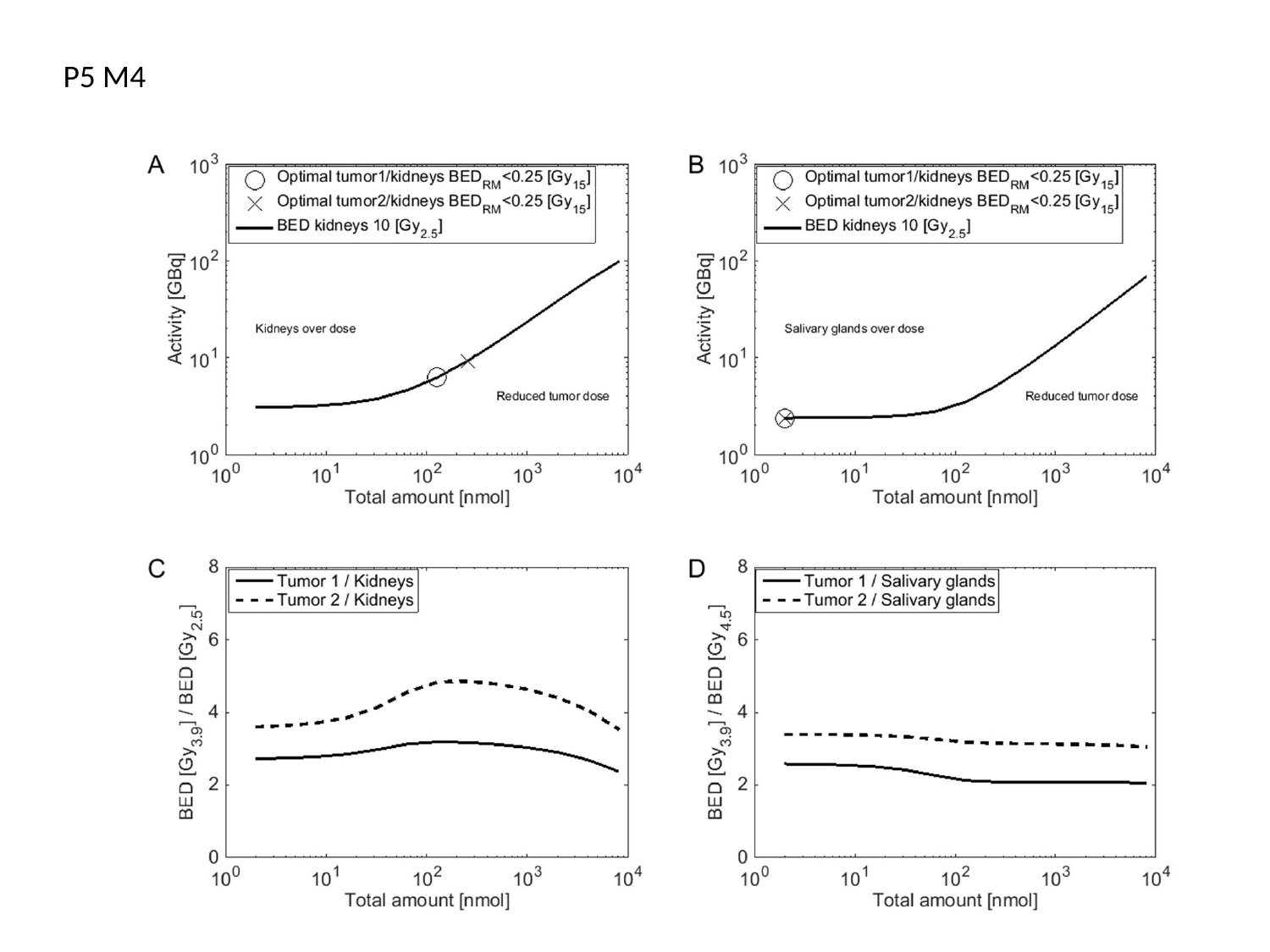

P5 M4
